# An optogenetic method for interrogating YAP1 and TAZ nuclear–cytoplasmic shuttling

**DOI:** 10.1242/jcs.253484

**Published:** 2021-07-09

**Authors:** Anna M. Dowbaj, Robert P. Jenkins, Daniel Williamson, John M. Heddleston, Alessandro Ciccarelli, Todd Fallesen, Klaus M. Hahn, Reuben D. O'Dea, John R. King, Marco Montagner, Erik Sahai

**Affiliations:** 1 Tumour Cell Biology Laboratory, The Francis Crick Institute, 1 Midland Road, London, NW1 1AT, UK; 2 School of Mathematical Sciences, University of Nottingham, Nottingham, NG7 2RD, UK; 3 Advanced Imaging Center, Janelia Research Campus, HHMI, Ashburn, VA 20147, USA; 4 Advanced Light Microscopy, The Francis Crick Institute, 1 Midland Road, NW1 1AT, London, UK; 5 Department of Pharmacology, University of North Carolina, Chapel Hill, NC 27599-7365, USA; 6 Department of Molecular Medicine, University of Padova, Viale G. Colombo 3, 35126 Padova, Italy

**Keywords:** YAP1, TAZ, Nuclear–cytoplasmic shuttling, Optogenetics

## Abstract

The shuttling of transcription factors and transcriptional regulators into and out of the nucleus is central to the regulation of many biological processes. Here we describe a new method for studying the rates of nuclear entry and exit of transcriptional regulators. A photo-responsive LOV (light–oxygen–voltage) domain from *Avena sativa* is used to sequester fluorescently labelled transcriptional regulators YAP1 and TAZ (also known as WWTR1) on the surface of mitochondria and to reversibly release them upon blue light illumination. After dissociation, fluorescent signals from the mitochondria, cytoplasm and nucleus are extracted by a bespoke app and used to generate rates of nuclear entry and exit. Using this method, we demonstrate that phosphorylation of YAP1 on canonical sites enhances its rate of nuclear export. Moreover, we provide evidence that, despite high intercellular variability, YAP1 import and export rates correlate within the same cell. By simultaneously releasing YAP1 and TAZ from sequestration, we show that their rates of entry and exit are correlated. Furthermore, combining the optogenetic release of YAP1 with lattice light-sheet microscopy reveals high heterogeneity of YAP1 dynamics within different cytoplasmic regions, demonstrating the utility and versatility of our tool to study protein dynamics.

This article has an associated First Person interview with Anna M. Dowbaj, joint first author of the paper.

## Abstract (precis)

**Summary:** We report a new method for quantification of nuclear import–export rates based on optogenetic reversible release of mitochondria-tethered proteins and validate the method using the transcription factors YAP1 and TAZ.

## INTRODUCTION

Rapid regulation of cellular processes in space and time is mostly achieved either by switching on inactive proteins in the correct location, or by recruiting proteins to the appropriate subcellular compartment at the right time. Shuttling of proteins between compartments is a key mechanism of regulating many processes, including the transcription of DNA in the nucleus (Fu et al., 2018; Xu and Massague, 2004; Di Ventura and Kuhlman, 2016). Transcription factors and transcriptional regulators are examples of proteins regulated by compartmentalisation, and their nuclear accumulation is often restricted in the absence of activating signals, either by cytoplasmic tethering or constitutive nuclear export (Xu and Massague, 2004; Fu et al., 2018; Di Ventura and Kuhlman, 2016). Importantly, the function of many transcription factors and regulators involves the integration of several signalling inputs. The specificity of the response is dictated by the modulation of the frequency, intensity and duration of nuclear–cytoplasmic (NC) shuttling rather than linear nuclear accumulation (Hao and O'Shea, 2011; Purvis and Lahav, 2013; Gao et al., 2018; Yosef and Regev, 2011; Behar and Hoffmann, 2010). This highlights the importance of dynamic, live, non-destructive techniques for studying NC shuttling.

YAP1 and TAZ (also known as WWTR1) transcriptional regulators are an example of such complexity. They serve as a hub for a wide range of stimuli, including biochemical (Hippo signalling) (Meng et al., 2016) and metabolic pathways (Sorrentino et al., 2014; Bertolio et al., 2019), mechanical inputs (rigidity, shape, stiffness, cell density and shear stress) (Wada et al., 2011; Calvo et al., 2013; Benham-Pyle et al., 2015; Aragona et al., 2013; Dupont et al., 2011; Chaudhuri et al., 2016; Nakajima et al., 2017; Elosegui-Artola et al., 2017), polarity and other signalling cascades (including those involving G-protein-coupled receptors, AKT and JNK) (Calvo et al., 2013; Codelia et al., 2014; Basu et al., 2003). YAP1 and TAZ are master regulators of cell proliferation, apoptosis and phenotypic plasticity (Zanconato et al., 2019; Shreberk-Shaked and Oren, 2019), and unsurprisingly they are heavily implicated in tumorigenesis (Zanconato et al., 2016). In the canonical view of the pathway, YAP1 and TAZ activity is inhibited by nuclear exclusion upon phosphorylation of LATS1/2 (LATS1 and LATS2; the main kinases of the Hippo pathway downstream of MST1 and MST2) on key serine residues, and subsequent binding to 14-3-3 proteins or proteasomal degradation (Fig. S1A). When LATS1/2-mediated phosphorylation is low, YAP1 and TAZ can accumulate in the nucleus and bind to their DNA-binding partners TEAD1–TEAD4 (Zhao et al., 2008), which also contributes to their nuclear accumulation. However, this simplistic model has been challenged by recent findings that LATS-phosphorylated YAP1 can be found in the nucleus and that nuclear accumulation is not sufficient for YAP1-driven transcriptional activity (Ege et al., 2018; Wada et al., 2011). Moreover, several LATS-independent mechanisms have been shown to affect YAP1 and TAZ NC shuttling and activity, such as nuclear deformation (Elosegui-Artola et al., 2017), the cell cycle (Manning et al., 2018), Src family kinases (Calvo et al., 2013; Ege et al., 2018; Elbediwy et al., 2016; Rosenbluh et al., 2012; Tamm et al., 2011) and, more recently, phase separation (Lu et al., 2020; Cai et al., 2019).

Historically NC shuttling has been investigated using static methods on fixed cells or subcellular fractions, with low temporal and spatial resolution, and by using chemicals such as the export inhibitor leptomycin B. More recently, measurement of protein shuttling has been revolutionised by the use of fluorescent dyes and proteins (Molenaar and Weeks, 2018; Di Ventura and Kuhlman, 2016). Without perturbation, the partitioning of fluorescent signal between compartments reflects the equilibrium position of the various shuttling processes influencing the fluorescently tagged protein. The application of light-driven perturbations, such as photobleaching, provides more information about rates of transit between compartments. However, one downside of photobleaching is that it is inherently destructive, which hampers making repeated measurements and typically generates only a single intensity decay curve, thereby limiting the effectiveness of mathematical fitting approaches to derive shuttling rate constants.

During the past decade, several optogenetic systems have been developed to control NC localisation of proteins within a few seconds upon illumination. Early examples of light-mediated NC shuttling (Engelke et al., 2014; Crefcoeur et al., 2013) led to irreversible nuclear accumulation, and they have been surpassed by reversible methods based on light-sensitive proteins. The common idea underlying optogenetic systems is the light-dependent conformational change of photoreceptors fused to the proteins of interest, which can be exploited to manipulate a wide range of cellular processes (de Mena et al., 2018). Control of protein localisation upon illumination has been achieved using two strategies so far: two-component systems, in which a bait protein is anchored to one compartment and the interaction with the prey protein is light-dependent (Guntas et al., 2015; Kennedy et al., 2010; Beyer et al., 2015; Strickland et al., 2012), and one-component systems, in which nuclear export signal (NES) or nuclear localisation signal (NLS) peptides are caged or exposed upon light illumination (Niopek et al., 2014, 2016; Yumerefendi et al., 2015, 2016). Interestingly, the former strategy has been recently used to develop light-induced control of cellular forces and, as a consequence, of YAP1 localisation (Valon et al., 2017). Recently, light-induced transcriptional activation of YAP1 has been achieved by uncaging an exogenous NLS (Illes et al., 2021). In this work, we develop an optogenetic tool, LOVTRAP (Wang and Hahn, 2016; Wang et al., 2016a), for studying NC shuttling based upon light-induced release and sequestration of the protein of interest. We measure the nuclear import and export rate constants of mCherry, YAP1, YAP1 mutants and TAZ. Of note, we find that, despite high intercellular variability, import and export rate constants are correlated within the same cell. We demonstrate that multiple proteins can be recorded simultaneously within the same cell, leading to the observation that YAP1 and TAZ rate constants are correlated. Taken together, this work provides detailed methods for the molecular biology, imaging and analysis required for the use of this tool.

## RESULTS

### An opto-release system for studying subcellular shuttling

**Fig. 1.**
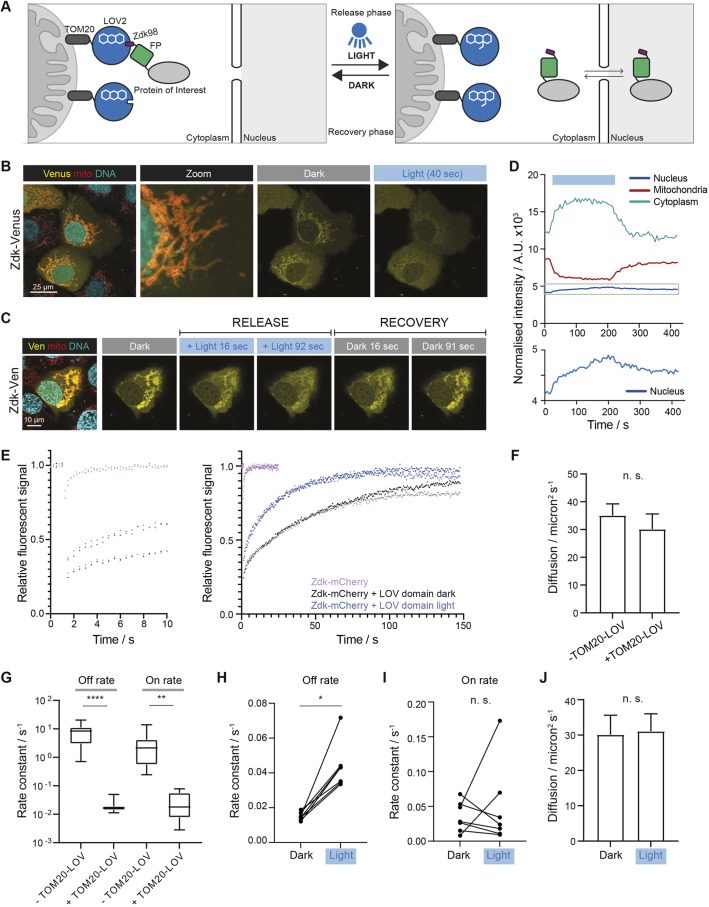
**Establishment and analysis of a system for light-dependent release of proteins from sequestration.** (A) Diagram shows the opto-release system design. In the dark, Zdk-tagged protein of interest is sequestered on the mitochondria through the interaction with the light-sensitive LOV domain. The LOV domain is tethered to the mitochondria through the TOM20 protein anchor. Upon blue light illumination, the LOV domain changes conformation and releases the Zdk-tagged protein. (B) Representative images of cells expressing the Zdk–Flag–Venus (yellow) construct without and with blue light illumination in the presence of sequestering TOM20–Flag–LOV (transient transfection in HaCaT cells). Mitochondria (mito; red) and DNA (cyan) are visualised in live cells using MitoTracker and DRAQ5, respectively. The zoomed-in image highlights Zdk–Flag–Venus localisation to the outer mitochondrial membrane. (C) Representative images from a movie of release (period of blue light illumination) followed by recovery (cessation of blue light illumination) of the Zdk–Flag–Venus (Ven; yellow) construct from TOM20–Flag–LOV in a transiently transfected HaCaT cell. Mitochondria (red) and DNA (cyan) are visualised in live cells using MitoTracker and DRAQ5, respectively. (D) Quantification of normalised intensity of mitochondria, cytoplasm and nucleus compartments corresponding to the optogenetic release and recovery experiment performed in C. Intensity is corrected for bleaching and background signal. Lower panel shows the nuclear compartment using an expanded *y*-axis scale (for the region highlighted by the grey box in the upper panel). The blue box indicates the period of blue light illumination. A.U., arbitrary units. Data are representative of 29 cells from six experiments, including control experiments using either Zdk–Venus or Zdk–mCherry. (E) Graph showing relative Zdk–Flag–mCherry fluorescence intensity in the bleached region following photobleaching in the absence of TOM20–Flag–LOV sequestration (purple and lilac, indicating data from two exemplar cells) or the presence of TOM20–Flag–LOV sequestration (black, grey, dark blue and light blue). Two exemplar cells are shown for each condition with distinct shading for each cell. The black and dark blue data points are from the same cell either in the dark (black) or under blue light (dark blue). Similarly, grey and light blue data points are from the same cell either in the dark (grey) or under blue light (light blue). The graph on the left represents the first 10 s of the experiment, while the graph on the right shows the full duration of the experiment. Data are representative of 22 cells from three experiments. (F) Bar graph (median+95% c.i.) of Zdk–Flag–mCherry diffusion rates in the presence or absence of TOM20–Flag–LOV sequestration; *n*≥13 cells from at least two experiments. (G) Boxplot of Zdk–Flag–mCherry off rate constants (left) and on rate constants (right) in the presence and absence of TOM20–Flag–LOV sequestration; *n*≥13 cells per condition from at least two experiments. Boxes represent the interquartile range, lines mark the median and whiskers show the range. (H) Paired values of mitochondria off rate constant of Zdk–Flag–mCherry release from the mitochondria in the dark or with blue light illumination in the same cell, derived using the FRAP methodology; *n*=7 cells. (I) Paired values of mitochondria on rate constant of Zdk–Flag–mCherry recovery to the mitochondria in the dark or with blue light illumination in the same cell, derived using the FRAP methodology; *n*=7 cells. (J) Bar graph (median+95% c.i.) of Zdk–Flag–mCherry diffusion rates in the dark or with blue light illumination; *n*≥13 cells per condition. All cells were co-transfected with TOM20–LOV. *P<0.05; **P<0.01; ****P<0.0001; n.s., not significant (Mann–Whitney test).

To establish an optogenetic system for protein shuttling studies, we adapted the LOVTRAP system (Wang and Hahn, 2016; Wang et al., 2016a). A light-responsive LOV (light–oxygen–voltage) domain was fused to the mitochondrial tether TOM20, and its synthetic peptide binding partner Zdk98 (referred to hereafter as Zdk) was fused to the protein of interest labelled with a fluorescent protein (FP) (Fig. 1A; Fig. S1B). We chose to fuse the Zdk peptide to the protein of interest because its smaller size, compared to that of the LOV domain, means that it is less likely to interfere with the dynamics of the protein being studied. Upon blue light illumination, LOV and Zdk dissociate, releasing the fusion protein into the cytoplasm. We selected the outer surface of mitochondria as the site of sequestration because the distinctive and discrete morphology of this organelle is well suited to image segmentation. Furthermore, the outer membrane is directly in contact with a large area of the cytoplasm. Yellow and red FPs – Venus and mCherry, respectively – were chosen to be fused to Zdk because their excitation wavelengths are long enough to exclude the possibility of triggering a conformational change in the LOV domain. Furthermore, their emission spectra are sufficiently different to enable simultaneous analysis of the distribution of both FPs, thereby raising the possibility of tracking the dynamics of two proteins simultaneously.

### Validation of opto-release of Zdk fusions from mitochondria and determination of rate constants

We began by testing whether a fusion of the Zdk peptide to mCherry was capable of localising an FP to mitochondria in the presence of the TOM20–LOV protein. Fig. 1B and Fig. S1C show that Zdk fusion constructs localised to mitochondria when co-transfected with TOM20–LOV, but not in the absence of TOM20–LOV. Staining using the Flag epitope tag enabled us to estimate that the TOM20–LOV fusion was expressed at roughly double the level of the Zdk–FP fusion (Fig. S1D). To visualise mitochondria and the nucleus concurrently with Zdk–Venus, we employed MitoTracker Red and DRAQ5. Fig. 1B shows that this approach labels the nucleus and mitochondria in the expected way. We next investigated the effect of 458 nm light illumination, which led to reduced mitochondrial localisation and increased Zdk–Venus in both the cytoplasm and nucleus (Fig. 1B). Upon cessation of illumination, the Zdk–Venus fusion exhibited a progressive transition back to the mitochondria (Fig. 1C; Movie 1). Quantification of the fluorescent signal in mitochondrial, cytoplasmic and nuclear compartments confirmed the release and re-binding of Zdk–Venus to the mitochondria (Fig. 1D). In the example shown, blue light illumination leads to a shift of approximately 15% of the total pool of Zdk–FP to transit from the mitochondria to the cytoplasm (Fig. 1D). Of note, the rate of fluorescence increase and decrease in the nucleus was much slower than that in the cytoplasm. This led to less visibly pronounced changes in fluorescence intensity in the nucleus; nonetheless, the increased nuclear fluorescence was readily quantifiable (Fig. 1D, lower panel). This is most likely related to the rate of nuclear entry and exit of Zdk–Venus being slower than the kinetics of release and re-binding of the Zdk peptide to the LOV domain. We reasoned that this difference could be exploited to gain quantitative information about the rates of nuclear import and export of proteins. To perform this analysis, an understanding of the dynamics of the interaction between the Zdk peptide and LOV domain in both dark and light conditions is required. Therefore, we implemented fluorescence recovery after photobleaching (FRAP) analysis of Zdk–FP fusions, employing a mathematical framework that was able to determine whether the Zdk–FP fusion was binding to an immobile partner, as well as the associated on and off rates (Fig. 1E; Williamson et al., 2021). Fig. 1F shows that Zdk–FP diffusion was estimated at ∼30 μm^2^ s^−1^ both in the presence and absence of TOM20–LOV, which is in close agreement with previous reports. In contrast, substantially different binding of Zdk–FP to an immobile partner was determined when the TOM20–LOV construct was expressed. In the presence of TOM20–LOV, the inferred off rate constants were ∼500 fold smaller (Fig. 1G), which is consistent with interaction of Zdk–FP with TOM20–LOV. The large difference also suggests minimal interaction of Zdk–FP with other cellular proteins in the absence of TOM20–LOV. The wide numerical range of on rate constants in the absence of TOM20–LOV is due to the effect of noise if there is no immobile partner present in the system. On and off rate constants for Zdk–FP binding to an immobile partner in the presence of TOM20–LOV were 0.027 s^−1^ and 0.019 s^−1^, respectively. The faster on rate indicates that the equilibrium position favours binding. The central premise of our experimental strategy is that blue light will release Zdk–FP fusion proteins from the mitochondria; therefore, we measured the on and off rate constants for the interaction between Zdk–FP and TOM20–LOV when cells were illuminated. Fig. 1H shows that the off rate constant increased when cells were illuminated with blue light. The on rate constant did not show a consistent change under blue light illumination (Fig. 1I). Diffusion was not affected by blue light (Fig. 1J).

### Supervised semi-automated segmentation of cellular compartments using a MATLAB app

**Fig. 2.**
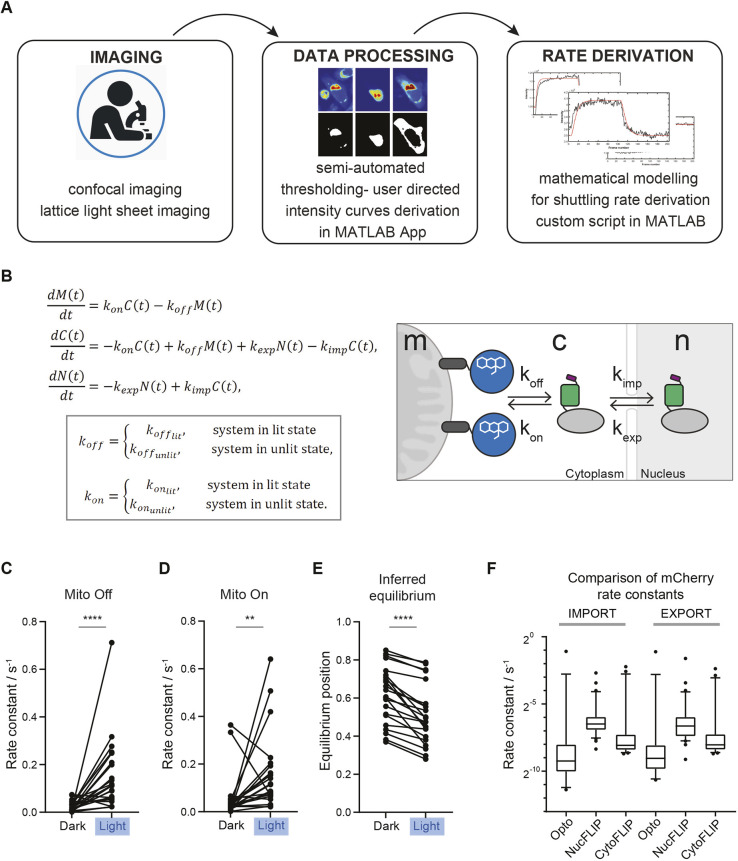
**Derivation of nucleocytoplasmic shuttling rate constants using opto-release and mathematical modelling.** (A) Experimental pipeline. Confocal imaging is followed by semi-automated intensity thresholding and shuttling rate analysis with mathematical modelling. (B) System of ordinary differential equations used to derive the shuttling rates of import, *k*_*imp*_; export, *k*_*exp*_; rate of mitochondrial release, *k*_*off*_; and rate of mitochondrial recovery, *k*_*on*_ for the nucleus, *N*(*t*), cytoplasm,*C*(*t*) and mitochondria, *M*(*t*) at time *t*. The box indicates that *k*_*off*_ and *k*_*on*_ can have different values in the dark and light. A schematic representation of shuttling is shown in the right (c, cytoplasm; m, mitochondrion; n, nucleus). (C) Paired values of mitochondria off rate constant of Zdk–Flag–mCherry release from the mitochondria without (dark) or with blue light illumination in the same cell, derived using opto-release methodology; *n*=22 cells from three experiments. (D) Paired values of mitochondria on rate constant of Zdk–Flag–mCherry recovery to the mitochondria without or with blue light illumination in the same cell, derived using opto-release methodology; *n*=22 cells from three experiments. (E) Paired values of mitochondria on rate constant divided by the sum of the on and off rate constants in the dark and light for the Zdk–Flag–mCherry construct, derived using opto-release methodology; *n*=22 cells. Higher values indicate an equilibrium position favouring mitochondrial binding. (F) Boxplot showing the nuclear import and export rate constants for Zdk–Flag–mCherry, derived using nuclear FLIP (NucFLIP), cytoplasmic FLIP (CytoFLIP) and opto-release (Opto) methodologies; *n*≥16 cells per condition from three experiments. Boxes represent the interquartile range, lines mark the median and whiskers show the 10–90th percentiles. **P<0.01; ****P<0.0001 (Mann–Whitney test).

To accurately extract quantitative information about the localisation of the fluorescent Zdk fusion, we developed a supervised MATLAB app for thresholding the different cellular compartments (Fig. 2A). Upon loading the imaging data and defining the appropriate channels, the region containing the cell of interest can be selected. Percentile intensity projections of all frames in each relevant channel are made at levels appropriate to bring out the features of each compartment whilst minimising noise and interference from neighbouring cells (Fig. S2A, panel i). The percentile projections then have thresholds applied to define appropriate compartment boundaries. The appropriate threshold for identification of the whole cell can then be set using a sliding tool that provides a real-time image of the result of the thresholding (Fig. S2A, panel ii). Once an appropriate value has been selected, the process is repeated to determine thresholds for segmentation of the mitochondria, and then the nucleus (Fig. S2A, panel iii). In the analysis presented here, nuclear segmentation was based on an FP–histone H2B fusion protein and mitochondrial segmentation was based on MitoTracker. Thresholding of the cytoplasmic compartment was used to define a region around the nucleus, with mitochondrial regions automatically excluded. Once the thresholds are set, the app generates both visual plots and a numerical data file to allow model fitting and the estimation of rates of binding and unbinding of the Zdk–FP fusion protein from mitochondria, as well as its rates of nuclear entry and exit (Fig. S2A, panel iv). To account for possible changes in the intensity of fluorescent cellular, nuclear and mitochondrial labels during imaging, the thresholding parameters can be redefined at various points during the time series. The result of this dynamic thresholding can then be compared with constant threshold values, and the method giving smaller errors in fitting can be selected for subsequent quantitative analysis (see Materials and Methods for more details).

### Determination of nuclear import and export rate constants and comparison with photobleaching methods

Having successfully established a tool for extracting numerical data of the localisation of the Zdk fusion protein of interest over time, we then sought to perform quantitative analysis. We used ordinary differential equations (ODEs) to model our experimental setup as a system with three key variables: the amount of Zdk fusion protein bound to mitochondria, the amount in the cytoplasm and the amount in the nucleus (Fig. 2B). Transitions were permitted from the mitochondria to the cytoplasm (and vice versa) and from the cytoplasm to the nucleus (and vice versa). This system of equations assumes that diffusion rates are sufficiently fast relative to transitions on and off the mitochondria and between the cytoplasm and nucleus. The measurement of 30 μm s^−2^ for Zdk–FP diffusion compared to mitochondrial binding and unbinding rate constants in the range 0.01–0.05 s^−1^ indicates that this assumption is justified (a detailed mathematical justification of this is provided in the Materials and Methods section ‘Mathematical derivation of the FRAP and optogenetic models’). The rate of transition on and off mitochondria, in both dark and light conditions, as well as nuclear import and export rate constants, were fitted simultaneously from the system of ODEs. To account for any photobleaching, we applied a normalisation method to ensure that the total level of fluorescence was constant over time (described in more detail in the Materials and Methods).

Fig. 2C–F show the mitochondrial on and off rate constants and the nuclear import and export rate constants as inferred from fitting to the levels of Zdk–FP fusion on mitochondria and in the cytoplasm and nucleus. As expected, the rates of transit on and off the mitochondria were considerably faster than the nuclear transit rates. Reassuringly, the values for mitochondrial on and off rate constants measured by our optogenetic tool were in agreement with those measured using FRAP (Fig. 1). We also noted that the on rate constant was somewhat higher under blue light illumination (Fig. 2C,D); however, in all cases, blue light shifted the ratio of on and off rate constants in favour of the off rate constant (Fig. 2E). Importantly, the inferred rates showed no correlation with either the total or mean fluorescence intensity of each cell (Fig. S2B). This indicates that our method is robust to variations in the expression level of the Zdk–FP fusion protein.

Unlike destructive FRAP and fluorescence loss in photobleaching (FLIP) measurements, our method is reversible, and this enables richer datasets to be generated and allows repeated measurements to be made in the same cell. We exploited these features to extract information about the changing localisation of Zdk–FP fusions during both the phase of the protein being released from mitochondria (blue light illumination) and the phase of re-binding to mitochondria (after cessation of blue light illumination). Fig. S2C shows the advantage that this confers over analysing only the release phase or only the re-binding phase, with fourfold and eightfold reductions in the intervals of plausible fitted parameter values when both release and re-binding phases were used, compared to those calculated using only the release or re-binding phase, respectively. We also independently measured the rates of nuclear entry and exit using a conventional FLIP method. In this method, we continuously depleted the Zdk–FP fluorescence at a location either in the cytoplasm (cytoFLIP) or the nucleus (nucFLIP) and recorded the diffusion of bleached fluorophore in the other compartment. Fig. 2F shows good concordance between the rates measured using our opto-release method and those measured using cytoFLIP. Of note, when we performed nucFLIP with continuous bleaching at a point in the nucleus, then the rates of nuclear entry and exit diverged from those measured using both cytoFLIP and opto-release. More specifically, the inferred rates were faster. This is most probably due to direct photobleaching of the fluorophore in the cytoplasm above and below the nucleus. This highlights an important caveat of FLIP experiments – namely the choice of photobleaching location.

### Application of the opto-release methodology to YAP1 and TAZ

**Fig. 3.**
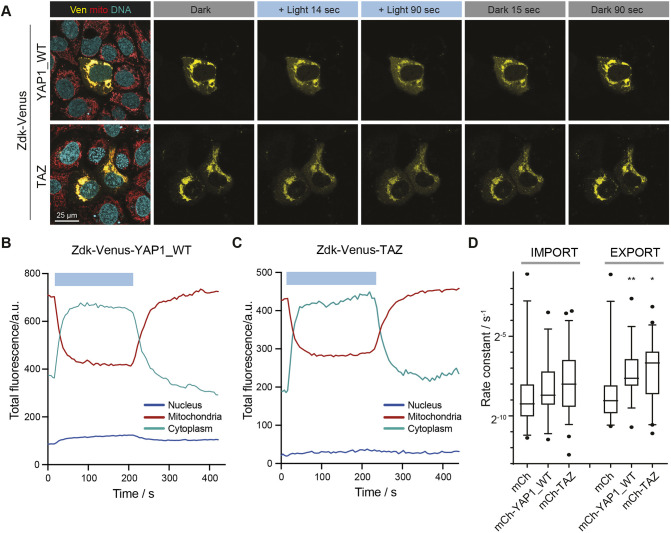
**Rates of YAP1 and TAZ nucleocytoplasmic shuttling in the HaCaT epithelial cell line.** (A) Representative images from a movie of release (period of blue light illumination) followed by recovery (cessation of blue light illumination) of the Zdk–Flag–Venus–YAP1-WT or Zdk–Flag–Venus–TAZ (Ven; yellow) construct from TOM20–Flag–LOV in transiently transfected HaCaT cells. Mitochondria (mito; red) and DNA (cyan) are visualised in live cells using MitoTracker and DRAQ5, respectively. (B,C) Quantification of normalized fluorescence intensity (a.u., arbitrary units) of mitochondria, cytoplasm and nucleus compartments corresponding to the optogenetic release and recovery experiment performed in A for (B) a Zdk–Flag–Venus–YAP1-WT cell and (C) a Zdk–Flag–Venus–TAZ cell. Intensity is corrected for bleaching and background signal. Blue boxes indicate the duration of blue light illumination. Data are representative of 44 cells for YAP1 and 32 cells for TAZ from four and six experiments, respectively. (D) Boxplot of the nuclear import and export rate constants for Zdk–Flag–mCherry (mCh), Zdk–Flag–mCherry–YAP1-WT (mCh–YAP1_WT) and Zdk–Flag–mCherry–TAZ (mCh–TAZ), derived using opto-release methodology. *n*≥15 cells per condition from three experiments. Boxes show the interquartile range, lines indicate the median and whiskers show the 10–90th percentiles. Asterisks indicate significant differences from Zdk–Flag–mCherry export. *P<0.05; **P<0.01 (Mann–Whitney test). The Zdk–Flag–mCherry data are re-plotted from Fig. 2F.

Having established that our method involving light-dependent release of a molecule from sequestration on the outer surface of mitochondria was capable of measuring nuclear entry and exit rates, we applied our method to measure the nuclear import and export rates of the transcriptional regulators YAP1 and TAZ (Fig. S1A). YAP1 and TAZ were fused to the Zdk peptide and either Venus or mCherry. Fig. S3A demonstrates that Zdk–FP–YAP1 was recruited to mitochondria in the presence of TOM20, whereas an FP–YAP1 fusion lacking the Zdk sequence was not recruited to mitochondria by TOM20–LOV. Immunostaining revealed that Zdk–FP–YAP1 typically had expression levels that were double that of the endogenous protein (Fig. S3B, with Fig. S3C confirming that the Zdk–FP fusion proteins had the expected size). Moreover, Fig. S3D confirms that these chimeric proteins retained their expected ability to drive transcription from a TEAD-dependent promoter, indicating that fusion with the fluorophore and Zdk peptide does not prevent the functionality of YAP1 and TAZ. Blockade of Crm1 (XPO1)-mediated nuclear export increased the nuclear localisation of Zdk–FP–YAP1, which further indicates that the fusion protein is subject to known regulatory mechanisms (Fig. S3E) (Ege et al., 2018). As before, blue light was used to release YAP1 and TAZ from sequestration, and their redistribution into the cytoplasm and nucleus was tracked. After 600 s, the blue light illumination was stopped, and the return of signal to the mitochondria was monitored. Fig. 3A–C and Movies 2 and 3 illustrate that both YAP1 and TAZ could be released from sequestration in the expected way, and that they re-bound upon cessation of blue light illumination. In line with expectations, the exit of YAP1 from the nucleus following the cessation of blue light illumination was reduced when Crm1 was inhibited (Fig. S3F). The app interface described above was then used to extract quantitative data that were then fitted to the model. These analyses revealed that both YAP1 and TAZ had marginally elevated nuclear import rates compared to mCherry, with TAZ rates being slightly faster than the rates of transit across the nuclear envelope for YAP1 (Fig. 3D). Consistent with expectations and with the data shown in Fig. 2, both YAP1 and TAZ showed faster mitochondrial off rate constants under blue light (Fig. S3G). Of note, both YAP1 and TAZ had significantly faster rates of nuclear export than mCherry (Fig. 3D). Once again, we confirmed that measurements obtained using optogenetic release and re-sequestration of YAP1 agreed with data obtained using more conventional FLIP methodology (Fig. S3H, note the similar position of black and red data points) and that there was no relationship between the rate constants and the expression level of the Zdk fusion protein (Fig. S2B).

### Active YAP1 is subject to reduced rates of nuclear export

**Fig. 4.**
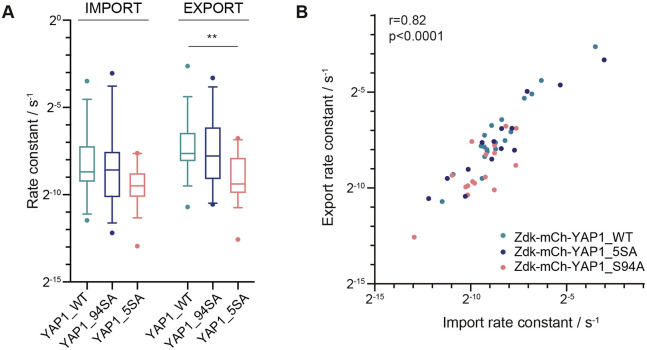
**Mutation of LATS phosphorylation sites reduces the rate of YAP1 nuclear export.** (A) Boxplot of the nuclear import and export rate constants for Zdk–Flag–mCherry–YAP1-WT, Zdk–Flag–mCherry–YAP1-5SA and Zdk–Flag–mCherry–YAP1-S94A, derived using the opto-release methodology. *n*≥15 cells per condition from three experiments. Boxes show the interquartile range, lines indicate the median and whiskers show the 10–90th percentiles. **P<0.01 (Mann–Whitney test). (B) Scatterplot of import versus export rate constants for Zdk–Flag–mCherry–YAP1-WT, Zdk–Flag–mCherry–YAP1-5SA, Zdk–Flag–mCherry–YAP1-S94A. *n*=49 from >3 experiments. Spearman's correlation coefficient (r) and significance are shown.

Compartmentalisation of YAP1 and TAZ is regulated by different post-translational modifications and, despite recent evidence for the importance of methylation and acetylation, the best characterised of these is phosphorylation. YAP1 and TAZ are negatively regulated by LATS1/2-dependent phosphorylation, downstream of the Hippo pathway, and conversion of these residues in YAP1 from serine to alanine generates a LATS1/2-insensitive YAP1-5SA mutant that shows higher transcriptional activity (Fig. S3D and Fig. S4A). In contrast, the YAP1-S94A mutant, which is unable to bind to TEAD transcription factors, is transcriptionally inactive and accumulates in the cytoplasm (Fig. S3D and Fig. S4A). We applied our optogenetic method to investigate whether YAP1-5SA and YAP1-S94A mutants exhibited different nuclear import and export rates. The YAP1 mutants exhibited the expected changes in mitochondrial binding and unbinding upon blue light illumination, with larger fold increases in unbinding rate constants, compared to binding rate constants, under blue light (Fig. S4B). Fig. 4A shows that the most prominent effect of the 5SA mutations was reduced nuclear export compared to that of the wild-type isoform (YAP1-WT). The YAP1-S94A mutant exhibited nuclear import and export rate constants comparable to the wild-type isoform. These data indicate that LATS1/2-dependent phosphorylation is required for effective nuclear export.

### Import and export rates are correlated

We were intrigued that the variance in import and export rates spanned more than an order of magnitude, and we investigated whether this variation might correlate with differences in cell or nuclear morphology, as has been suggested previously for nuclear import (Elosegui-Artola et al., 2017). Fig. S4C shows correlation analysis of import and export rates with a range of nuclear morphology parameters. No single morphological metric correlated clearly with either import or export. However, we noted a correlation between the relative size of the nucleus (Nuc/Cyto area) and the relative import and export rate constants (import/export ratio). The system of differential equations that we used to determine the import and export rates dictates that the ratio of import and export rates is directly related to the ratio of total nuclear to total cytoplasmic protein. However, this relationship does not require that the import/export ratio is correlated with the Nuc/Cyto area, as it could be satisfied if the protein concentration in the nucleus and cytoplasm fluctuated depending on the Nuc/Cyto area. The experimental observation of a correlation between Nuc/Cyto area and import/export ratio (Fig. S4D) suggests a homeostatic mechanism that increases nuclear import as the nucleus gets larger, thereby helping to maintain the nuclear concentration of proteins. We additionally observed that rates of nuclear import and export were consistently correlated. This is shown for YAP1 and the YAP1 mutants in Fig. 4B. Cells with high rates of YAP1 nuclear import also had high rates of nuclear export. These data suggest that the energetic cost of transit across the nuclear membrane might be highly variable between cells, but that this variation in cost applies to both nuclear entry and exit. Furthermore, even though there is considerable intercellular variation in the rates, the process remains subject to regulation, as YAP1-5SA had slower import and export rates than wild-type and TEAD binding-defective YAP1 (Fig. 4A).

### Implementation of the opto-release methodology on a lattice light-sheet platform

**Fig. 5.**
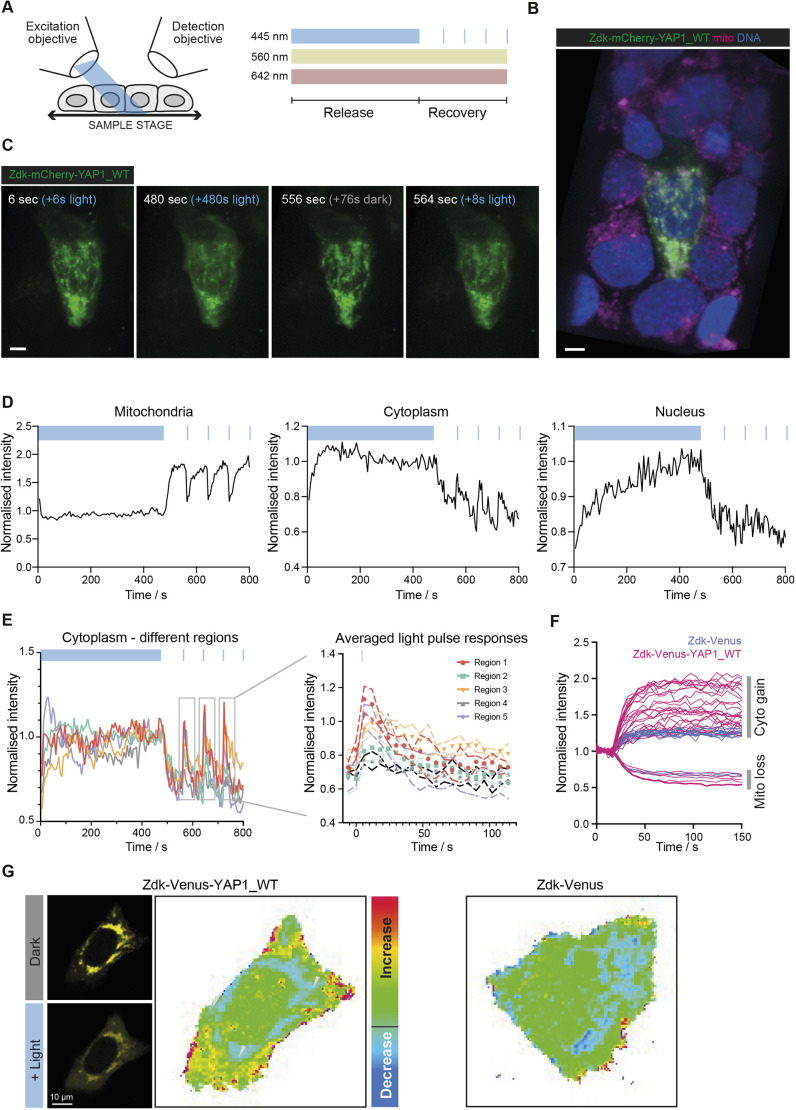
**Implementation of YAP1 dynamics on a lattice light-sheet microscope reveals heterogeneous cytoplasmic dynamics.** (A) Schematic diagram of the lattice light-sheet imaging experimental design. (B) Representative image of a HaCaT cell expressing Zdk–Flag–mCherry–YAP1-WT imaged on lattice light-sheet microscope. Mitochondria (mito; red) were stained using MitoTracker Deep Red, and DNA (blue) was visualised using stable expression of an H2B–FP fusion. Scale bar: 5 μm. (C) Release and recovery experiment of Zdk–Flag–mCherry–YAP1-WT in 3D. Scale bar: 5 μm. (D) Quantification of fluorescence intensity during an optogenetic release and recovery experiment performed in a HaCaT cell expressing Zdk–Flag–mCherry–YAP1-WT. The curves show exemplar intensities from regions of the mitochondria, the cytoplasm and the nucleus. Blue boxes indicate periods of blue light illumination. (E) Quantification of intensities corresponding to five different cytoplasmic regions during the 3D optogenetic experiment in a HaCaT cell expressing Zdk–Flag–mCherry–YAP1-WT; right panel shows average post-blue light spike intensity profiles for different cytoplasmic regions combining the three technical replicate spikes. Symbols show the mean of the three temporal replicates and dashed lines show the s.d. (black dashed lines correspond to the grey triangles) for the different cytoplasm regions of one representative cell. Data in D and E are representative of *n*=36 cells from seven independent experiments for YAP1-WT. (F) Analysis of different cytoplasmic regions in HaCaT cells transiently transfected with Zdk–Flag–Venus (lilac) or Zdk–Flag–Venus–YAP1-WT (magenta) plotted as normalised fluorescence intensity. Each line represents a different region of a cell, with upward lines corresponding to cytoplasm regions of interest and downward lines corresponding to mitochondrial regions. Data derived from ≥14 cytoplasmic regions of three cells for each condition. (G) Heatmap and representative images showing the proportionate gain in fluorescence after blue light illumination of HaCaT cells transiently transfected with Zdk–Flag–Venus or Zdk–Flag–Venus–YAP1-WT; refer to Fig. 1C for images corresponding to the Zdk–Flag–Venus heatmap. Arrowheads indicate regions of high fluorescence signal gain in Zdk–Flag–Venus–YAP1-WT.

YAP1 and TAZ can be associated with a wide array of binding partners in diverse subcellular compartments. Confocal analysis of a single optical section is not suited for analysis of the distribution of molecules throughout the cell. Therefore, we sought to implement our opto-release method on a lattice light-sheet microscope. The experimental design was adapted from the simple release and re-binding protocol, with additional single frames of blue light included at 80 s intervals in the re-binding phase (Fig. 5A). The additional blue light pulses were used to explore whether events with different kinetics might also be observed when the whole cell volume was acquired with high spatial resolution (104 nm *x*-*y* and 211 nm *z* resolution). Fig. 5B shows a 3D image of confluent HaCaT cells, with one cell expressing the Zdk–mCherry–YAP1 construct. As expected, the Zdk–FP–YAP1 fusion exhibited efficient localisation to the mitochondrial network in frame 1, but was widely distributed throughout the cell by frame 80 (480 s; a 3D view is shown for Zdk–FP–YAP1 in Fig. 5C and for Zdk–FP in Fig. S5A). Following the end of the long phase of blue light illumination, the YAP1 fusion returned to the mitochondria (Fig. 5C and Movie 4, 556 s; Movie 5 shows a control experiment with Zdk–mCherry). As expected, this localisation was diminished immediately following the pulses of blue light at 560, 640 and 720 s (Fig. 5D, left-hand panel). Quantification of the Zdk–FP–YAP1 signal from representative areas of the cytoplasm and nucleus is shown in Fig. 5D. These traces clearly show the pulsatile release of the YAP1 fusion into the cytoplasm, but this does not translate into changes in nuclear signal.

The purpose of performing high-resolution lattice light-sheet imaging was to determine whether the dynamics of the YAP1 fusion protein might vary depending on the precise subcellular localisation. We therefore quantified multiple cytoplasmic and nuclear regions. Despite the whole cell being illuminated with short pulses of blue light, the extent of gain in YAP1 signal and its duration appeared to vary from region to region in the cytoplasm. Some areas had a pronounced increase that decayed sharply (Fig. 5E, regions 1 and 5; the position of the regions is shown in Fig. S5B), whereas in other parts of the cytoplasm the increase was less pronounced but more durable (regions 2 and 3), or barely detectable at all (Fig. 5E; similar data for the cytoplasm of a different cell is shown in Fig. S5C). The changes in nuclear intensity profiles were noisier, which precluded more detailed analysis of differences between regions (Fig. S5D). To explore the differences between cytoplasmic regions more rigorously, we exploited the fact that there were three equal duration pulses in the time-series data. Therefore, we considered each pulse to be a ‘technical replicate’ and derived the average response for the different cytoplasmic regions. Fig. 5E shows the difference in the average responses, with statistically significant differences in peak intensity and the rate of signal decline after the peak shown in Fig. S5E. Furthermore, a low maximal intensity post-blue light pulse correlated with a slower decay of signal; these data are consistent with slower movement of YAP1 both into and out of these regions. These data suggest that YAP1 dynamics vary between different regions of the cytoplasm and illustrate the utility of combined optogenetic manipulation and lattice light-sheet imaging for studying subcellular variation in protein dynamics.

To validate the observation described above that YAP1 has localised variation in its behaviour in the cytoplasm, we returned to our confocal methodology. Specifically, we determined whether the maximal relative increase in YAP1 varied between different regions in the same cell. Fig. 5F shows the relative increase in cytoplasmic fluorescence for 40 different cytoplasmic regions of roughly 10 μm^2^ selected from multiple cells expressing Zdk–FP–YAP1. We observed considerable variation in dynamics in both the initial rate and maximum gain in fluorescence between different regions, which supports the light-sheet observations. In contrast, Zdk–FP behaved much more consistently across different regions. To determine the location of the regions with different YAP1 dynamics, we generated maps showing the proportionate gain in Zdk–FP–YAP1 fluorescence (Fig. 5G). These revealed a complex pattern that could not be explained simply by proximity to the nucleus or cell periphery. Once again, Zdk–FP showed little variation across different cytoplasm regions.

The low level of variation in Zdk–FP behaviour argues against cytoplasmic crowding being the source of variation in Zdk–FP–YAP1 behaviour. The local differences in dynamics could be due to subcellular differences in YAP1 diffusion and/or interaction with unknown partners. To address this, we returned to our FRAP-based analysis of diffusion and rate constants for binding and unbinding to an immobile partner protein. In particular, we analysed the variation in diffusion and binding rate constants between Zdk–FP and Zdk–FP–YAP1 across all the cytoplasmic regions considered. Fig. S5F shows that there was little difference in the measurements of diffusion between Zdk–FP and Zdk–FP–YAP1. In contrast, the unbinding rate constant derived for Zdk–FP–YAP1 was significantly different from that for Zdk–FP and exhibited a large variance between different regions (Fig. S5G; compare with Fig. 1G). Taken together, these data argue that the localised differences in YAP1 cytoplasmic dynamics result from interaction with a partner protein, not diffusion or cytoplasmic crowding.

### Simultaneous measurement of YAP1 and TAZ nuclear transit rates reveals that they are correlated

**Fig. 6.**
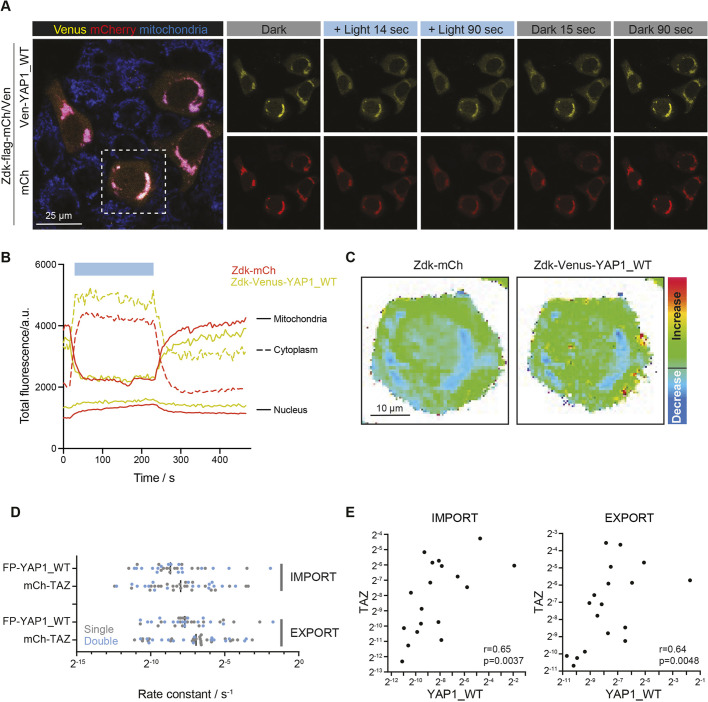
**Simultaneous measurements of YAP1 and TAZ shuttling reveal correlated nuclear import and export.** (A) Representative images from a movie of release and recovery of the Zdk–Flag–Venus–YAP1-WT (yellow) and Zdk–Flag–mCherry (red) constructs from TOM20–Flag–LOV in transiently transfected HaCaT cells. Mitochondria (blue) are visualised in live cells using MitoTracker. Dashed box indicates the cell shown in the heatmap in C. (B) Quantification of normalised intensity (a.u., arbitrary units) of mitochondria, cytoplasm and nuclear compartments corresponding to the optogenetic release and recovery experiment performed in A. Intensity has been corrected for bleaching and background signal. Blue box indicates the period of blue light illumination. Data are representative of 17 cells from two experiments. (C) Heatmap of Zdk–Flag–Venus–YAP1-WT and Zdk–Flag–mCherry (Zdk–mCh) construct fluorescence intensities in the same cell, as specified in A. (D) YAP1 and TAZ nuclear import and export rates measured either individually (single, grey) or simultaneously (double, light blue). Black bar indicates the median. (E) Scatterplot of YAP1 versus TAZ import (left) or export (right) rate constants in the same cell. *n*=18 from three experiments. Spearman's correlation coefficient (r) and significance are shown.

A particular benefit of the opto-release methodology described here is the relative ease with which two different proteins might be studied simultaneously in the same cell. To explore this, and to seek to confirm the differential cytoplasmic behaviour of Zdk–FP–YAP1 and Zdk–FP, we analysed cells expressing both Zdk–mCherry and Zdk–Venus–YAP1. Fig. 6A,B and Movie 6 show that both Zdk–mCherry and Zdk–Venus–YAP1 transition from mitochondria to the cytoplasm and nucleus under blue light illumination. This experimental configuration enabled us to perform spatial analysis of the proportionate gain for two different Zdk–FP proteins in the same cell (Fig. 6B). Once again, regions of high increase in YAP1 could be observed (Fig. 6C).

Having confirmed the feasibility of tracking two proteins in the same cell, we investigated whether the transit rates of different proteins across the nuclear envelope were correlated in the same cell. We applied our analytical app to Zdk–Venus–YAP1-WT and Zdk–mCherry–TAZ images from the same cell to extract nuclear import and export rate constants. Blue light illumination led to changes in mitochondrial off and on rate constants that were consistent with our previous analyses (Fig. S6). Moreover, the nuclear import and export rate constants measured using this ‘double’ system were overlapping with those measured using single fluorophore opto-release and cytoplasmic FLIP experiments (Fig. 6D; compare with Fig. S3H). Importantly, both export and import rates of YAP1 significantly correlated with those of TAZ, suggesting an overlap between the mechanisms regulating NC shuttling of the two proteins (Fig. 6E). Taken together, these experiments describe and demonstrate the utility of using light-dependent release and sequestration for studying the dynamics of proteins of interest. Using YAP1 and TAZ as exemplars, we have shown that there is regional variation in the cytoplasmic dynamics of YAP1, that phosphorylation of YAP1 is required for effective nuclear export, and that nuclear export and import of YAP1 and TAZ are highly correlated. We propose that the tools documented here will prove to be valuable for researchers studying the dynamics of subcellular protein distribution.

## DISCUSSION

Decoding the complex regulatory inputs governing the nuclear and cytoplasmic distributions of proteins requires accurate measurement of rates of nuclear entry and exit. During the past decade, several works have shown that an incredibly large number of different signals, from cellular architecture and microenvironment geometry to metabolic and biochemical pathways, converge on YAP1 and TAZ (Pocaterra et al., 2020). Here, we implement an optogenetic system to track the shuttling of fluorescently labelled YAP1 and TAZ proteins, which we have coupled to mathematical modelling to derive nuclear import and export rates. This system builds upon the previously reported LOVTRAP optogenetic tool, which allows protein dissociation within a fraction of a second upon illumination with blue light (450–490 nm). In our system, which we term ‘opto-release’, we exploit the light-induced dissociation of the LOV domain tethered to the mitochondrial surface from a previously identified synthetic peptide (Zdk) that is fused to fluorescently labelled YAP1 or TAZ, and we then monitor their subcellular distribution over time. FRAP analysis confirmed that blue light increases the unbinding rate of the Zdk fusion protein from the mitochondria (Fig. 1). Using this system, we were able to rapidly generate a pool of fluorescently labelled cytoplasmic YAP1 and TAZ that could be tracked in the cell after release from mitochondria (in blue light) and during recovery (in the dark). The LOVTRAP system has several advantages over other optogenetic systems for this purpose: (1) Zdk is a small peptide and does not interfere with activity of YAP1 and TAZ (Fig. S3D); (2) the LOV domain utilises an endogenous chromophore (a flavin mononucleotide), reducing external intervention during analysis; (3) the system allows the study of multiple fluorescently labelled proteins with sufficient spectral separation, such as mCherry and Venus (Fig. 6); and (4) it allows rapid, reversible and non-destructive analysis, unlike FLIP. We have exploited this last point to obtain more accurate measurements, either by fitting to both the release and sequestration phases of experiments or by performing repeated cycles of light pulses.

Using our opto-release system, we have measured YAP1 and TAZ shuttling rates. Despite being larger than mCherry, both YAP1 and TAZ shuttled out of the nucleus faster (Fig. 3D). This indicates active mechanisms of export. The rate of YAP1 transit that we measured in epithelial HaCaT cells was slightly slower than that which we previously reported for YAP1 in fibroblasts using FRAP and FLIP methodology (Ege et al., 2018). Direct comparison of opto-release with FLIP methods in HaCaT cells showed good concordance when the photobleaching was performed in the cytoplasm. The discordant results obtained for nuclear photobleaching are probably due to direct photobleaching of the fluorophore in the cytoplasmic regions of the cell directly above and below the nucleus, which is likely to be a widespread issue when using cuboidal epithelial cells, such as HaCaT.

In our analysis, mutation of LATS phosphorylation target sites on YAP1 led to reduced nuclear export that can explain YAP1-5SA nuclear accumulation (Fig. 4A). YAP1 lacks a canonical NLS (Wang et al., 2016b), and we observed less clear-cut changes in nuclear import rate constants, either between mCherry and YAP1 or between YAP1 and its mutants. Intriguingly, the YAP1-S94A mutant, which is unable to bind TEAD, is localised in the cytoplasm at steady state, and in our analysis its import and export rate constants were close to those calculated for YAP1-WT, even though the mutant was observed to be more cytoplasmic than the wild-type YAP1 (compare Fig. 4A and Fig. S4A). This could be explained by the existence of a pool of wild-type YAP1 that remains sequestered in the nucleus with binding and unbinding rates that are too slow to measure in the few minutes duration of our assays. Alternative explanations could also involve cytoplasmic partners for YAP1 (Kanai et al., 2000; Azzolin et al., 2014; Varelas et al., 2010; Zhao et al., 2010). Indeed, both light-sheet and confocal analyses indicated that YAP1 exhibits heterogeneous dynamics in different cytoplasmic areas. The ability to implement opto-release methodology on light-sheet platforms offers significant scope for more complex and comprehensive analysis of protein dynamics using high-resolution volumetric data from whole cells. The mathematical modelling of spatial information in addition to temporal information using partial differential equations, as in Ege et al. (2018), will also allow us to separate import and export from motility within each compartment. Solving the issue of heterogenous YAP1 dynamics in the cytoplasm will require a more complex model with a larger number of compartments and more spatial features. It will also require implementation of 3D segmentation tools for different cellular compartments. It will be interesting to perform longer release-phase experiments, allowing the released YAP1 to reach equilibrium distribution in all subcellular compartments and analysing both rapid and slow rates of transit between different compartments. The reversibility of the opto-release tools means that different lengths and sequences of blue light illumination can be used in a single experiment to obtain information relevant to different timescales.

Interestingly, we confirmed that nuclear import and export rates are correlated within the same cell but show high intercellular variability, as has been observed in *Drosophila* (Manning et al., 2018). Our conclusion that nuclear export is the main mechanism for the regulation of YAP1 wild-type activity differs from evidence in *Drosophila*, where Yorkie (the *Drosophila* homologue of YAP1) is mainly regulated by tuning nuclear import (Manning et al., 2018). A possible explanation for this discrepancy is that Yorkie lacks the PDZ-binding motif that is required for the nuclear accumulation of YAP1 in mammals (Wang et al., 2016b; Oka and Sudol, 2009). By releasing two proteins simultaneously, we could also show that YAP1 and TAZ import and export rates co-varied, suggesting that they may share some regulatory inputs, with LATS-mediated phosphorylation being an obvious candidate. The import/export rate constant ratio correlated with Nuc/Cyto area for all proteins tested, suggesting that cells have a mechanism for maintaining the relative concentration of proteins in the nucleus and cytoplasm even if the relative size of the two compartments varies. More specifically, our analyses suggest that increasing cytoplasmic area shifts the balance towards lower nuclear import. We did not observe a link between nuclear import and nuclear shape factors, as suggested by Elosegui-Artola et al. (2017). However, we did not apply any direct or indirect perturbations to the nuclear envelope.

In this study, we report the use of a reversible optogenetic system based upon the LOVTRAP system (Wang and Hahn, 2016; Wang et al., 2016a). This methodology allows controlled release of fluorescent proteins that can be tracked over time in the different cell compartments. The reversibility of the optical release enables more complex experimental design than is possible with conventional FRAP and FLIP analyses. In particular, by varying the timing and length of release and re-binding phases it is possible to acquire data about processes happening with different timescales in the same experiment. Alternatively, repetitive use of a signal illumination pulse or sequence can effectively generate ‘technical replicates’ in a single experiment, leading to more accurate measurements. We generated a MATLAB app for thresholding of different cellular compartments and compensation for cell motion. This tool can extract numerical values for NC localisation that are used by differential equations to generate import and export rates. While we have used this system to gain further insights into YAP1 and TAZ NC shuttling, the modular nature of the constructs will make it easy to adapt to a range of transcription factors and regulators, including SMADs, NF-kB, MRTF, IRFs, and STATs. We anticipate that opto-release systems based on this framework will facilitate the understanding of how diverse inputs regulate the subcellular localisation of proteins and will be of wide utility to cell biologists.

## MATERIALS AND METHODS

### Cell lines and cell culture

HaCaT immortal keratinocyte cells were acquired from the Cell Services Facility at the Francis Crick Institute. Histone H2B–mTurquoise2 was introduced to cells using the PiggyBac transposon system, where the plasmid of interest is transfected together with a transposase plasmid PBase, as described previously (Ege et al., 2018). Cells expressing the construct were selected using puromycin. All the other plasmids were introduced transiently. Cells were cultured in DMEM (Gibco) with 10% foetal bovine serum (FBS; PAA Labs) and 1% penicillin-streptomycin (Pen-Strep; Gibco) at 37°C and 5% CO_2_.

### Plasmids

All the plasmids generated for the opto-release system were cloned using the Gibson Assembly System (NEB) by combining pRK5.1 backbone (Addgene) with the desired PCR fragments according to the manufacturer's instructions. The opto-release system was based on the previously published LOVTRAP system (Wang and Hahn, 2016; Wang et al., 2016a), but with the bulkier LOV domain tethered to mitochondria. The photo-switchable modified common oat (*Avena sativa*) light-oxygen-voltage (LOV) 2 domain from phototropin 1 protein was fused to a TOM20 protein fragment (amino acids 1–35), which anchors it to the outer mitochondrial membrane. On the other side of the system was the YAP1 or TAZ proteins, which were joined to Zdk, an engineered small protein domain based on the Z domain described previously (Wang and Hahn, 2016; Wang et al., 2016a), and fluorescent proteins (mCherry or Venus, both photostable in this system over the timescale of our experiments). YAP1 corresponds to the human YAP1-2γ isoform, which is 504 amino acids long (Sudol, 2013) and the YAP1 mutants were described previously (Ege et al., 2018). The five serines mutated for the YAP1-5SA mutant correspond to serines 61, 109, 127, 164 and 397 (serine 397 corresponds to serine 318 in other YAP1 splice isoforms) for the isoform used in this study. The two plasmids used for the luciferase experiments, pGL3-49 and pGL3-5×MCAT-49, were a gift from Nic Tapon, Francis Crick Institute, London, UK. The pPB-puro-H2B-mTurquoise2 was generated in the Sahai lab (Ege et al., 2018). Details of all plasmids used and their sources are provided in Table S1.

### DNA transfection

For transient transfection with plasmid DNA, cells were transfected with Lipofectamine LTX and Plus reagents in combination (15338100; Thermo Fisher Scientific) according to the manufacturer's instructions. One day prior to the transfection, cells were seeded at 0.5×10^6^–1×10^6^ cells per well for western blotting or luciferase transfection in a 6 well plate, 3×10^5^ cells per dish for imaging in a 35 mm MatTek dish (MatTek Co., Ashland, MA, USA), or 0.5×10^5^–1.25×10^5^ cells per well for live imaging and immunofluorescence in MatTek 24-well plates. Prior to transfection, the medium was changed to either Pen-Strep-free DMEM with 10% FBS, or OptiMEM. On the day of transfection, two tubes were prepared (using the proportions given for a 24-well plate). Tube 1 contained 100 μl OptiMEM, 1–2 μg DNA and 2 μl Plus reagent. Tube 2 contained 100 μl OptiMEM and 4 μl Lipofectamine LTX. The contents of each of the two tubes were mixed and incubated for 5 min separately, and then were mixed together and incubated for a further 5 min. The transfection mix was added dropwise to the cells and incubated for 4–6 h at 37°C. Subsequently, the transfection mix was removed, and DMEM containing 10% FBS and Pen-Strep was added. The ratio of plasmids for the expression of Zdk–FP and TOM20–LOV was either 1:1 or 2:1. Furthermore, prior to imaging, cells were visually selected to exclude those expressing very high levels of Zdk–FP or low/no expression of TOM20–LOV.

### Immunofluorescence

All immunofluorescence experiments were performed on cells seeded on glass in MatTek plates. Cells were fixed in 4% paraformaldehyde, washed with phosphate-buffered saline (PBS) containing 0.01% Triton X-100, and permeabilised by incubation in PBS containing 0.2% Triton X-100 for 20 min at room temperature. Samples were subsequently blocked for 1 h with PBS containing 5% BSA and 0.01% Triton X-100 before incubation with Phalloidin–Atto633 (Sigma; 68825-10NMOL) and DAPI (Sigma; d9542-5 mg) in PBS containing 5% BSA and 0.01% Triton X-100 for 1 h at room temperature. Primary antibodies used were anti-YAP1 (D8H1X; 14074; Cell Signaling Technology; 1:500), and anti-Flag (F1804; Sigma; 1:100), and were incubated overnight at 4°C. Primary antibody was washed off in three washes of 5 min using PBS containing 0.01% Triton X-100. Secondary antibodies used were donkey anti-rabbit IgG Alexa Fluor 488 (Thermo Fisher Scientific; A21206; 1:200) and donkey anti-mouse IgG Alexa Fluor 555 (Thermo Fisher Scientific; A31570; 1:200). Samples were retained in PBS until imaging.

### Luciferase assay

Luciferase assays were performed using a dual luciferase assay kit (Promega). Cells were lysed using passive lysis buffer (Promega). Lysates were placed into a white 96-well plate (Perkin Elmer) to assess firefly luciferase and *Renilla* luciferase activities using an Envision Multilabel plate reader (Perkin Elmer). To normalise, the measurements of firefly luciferase activity were normalised to the *Renilla* luciferase activity of the same sample.

### Western blotting

For protein analysis, cells were lysed with 1×SDS sample buffer (0.32 M Tris-HCl pH 6.8, 10% SDS, 50% glycerol, 3 M β-mercaptoethanol and 0.05% Bromophenol Blue) added directly to wells, and then cells were scraped and collected. Each sample was sonicated and then boiled at 95°C for 5 min before being used for western blotting. Samples were separated on SDS–PAGE gels (Bio-Rad). A pre-stained Dual Colour protein ladder (Bio-Rad; 1610374) was run with the samples. The proteins in the gel were transferred to polyvinylidene fluoride (PVDF) membrane (GE Healthcare Life Sciences). The membranes were blocked with 2% milk (Marvel) or 2% BSA in TBST (137 mM NaCl, 2.7 mM KCl, 20 mM Tris-HCl, pH 7.4 and 0.01% Tween 20) for 1 h at room temperature, and then incubated with primary antibody at 4°C overnight in TBST containing 0.1% milk or BSA. After three washes with TBST, membranes were incubated for 45 min at room temperature with HRP-labelled secondary antibody (1:50,000; anti-mouse IgG, 31430; anti-rat IgG, 31470; Thermo Fisher Scientific) in TBST containing 0.5% milk. The blot was then developed by rinsing for 1 min in Luminata WesternHRP Substrate (Millipore) before being exposed in an ImageQuant600RGB machine (GE Healthcare Life Sciences). The following primary antibodies were used: anti-YAP1 (D8H1X; 14074; Cell Signaling Technology; 1:500), anti-β-tubulin (T7816; Sigma; 1:5000), anti-RFP (5F8-100; Chromotek; 1:1000) and anti-Flag (F1804; Sigma; 1:1000).

### Statistical analysis

Statistical analyses were performed using Prism software (GraphPad Software). *P*-values were obtained using tests specified in the figure legends, with significance set at *P*<0.05. *P<0.05; **P<0.01; ***P<0.001, ****P<0.0001; ns, not significant.

### FRAP imaging

Following transient transfection with Zdk–Flag–mCherry or Zdk–Flag–mCherry–YAP1 with or without TOM20–LOV, cells were imaged in 35 mm MatTek dishes using a Zeiss LSM780 microscope with a blue light LED array mounted above a temperature-controlled stage. Zdk–mCherry constructs were imaged by excitation using a 561 nm laser with emission captured at 567–651 nm. Blue light illumination was provided by a bespoke array of 450 nm-emitting LEDs placed ∼5 cm above the imaging plane of the confocal microscope. Images were captured every 250 ms for analysis of Zdk–FP fusions in the absence of the LOV domain and every 500 ms for analysis in the presence of the LOV domain. Circular regions of interest (ROIs) in the cytoplasm of either 2.6 μm, 4.0 μm or 5.3 μm diameter and typically within 10 μm of the nucleus were selected by the user for photobleaching using 561 nm light at >20× the light intensity used for imaging. In the presence of the LOV domain, the ROIs were additionally positioned over the mitochondria. To ascertain the precise shape and efficiency of the photobleaching, a small number of experiments were conducted using cells that had already been fixed using 4% paraformaldehyde. This process was repeated using a sample of cells for each bleach region radius (sample sizes: *n*=6 for radius 10 pixels, *n*=5 for radius 15 pixels, and *n*=5 for radius 20 pixels). The average intensity profile was then computed for each bleach region radius. These empirically derived intensity profiles were used to simulate photobleaching in subsequent analyses. To derive diffusion and binding rate constant information, the .lsm files containing imaging data were loaded into MATLAB using the Open Microscopy Environment's bio-formats toolbox (https://docs.openmicroscopy.org/bio-formats/6.1.0/users/matlab/index.html). To determine the spatial extent of the cytoplasm for each cell, the mean intensity projection of all frames was calculated to form a single blended image, as a means of noise reduction. The position of the cytoplasm was then inferred from discontinuities in the fluorescence intensity using edge detection. Background intensity was measured in a user-defined reporting region outside of the cell boundary and subtracted from each frame. Photobleaching during the recovery phase, caused by imaging-laser light, was explicitly incorporated into the FRAP model that is summarised below and described in detail in Williamson et al. (2021) (see also ‘Mathematical derivation of the FRAP and optogenetic models’ in the Materials and Methods).

#### Mathematical model

A time-dependent and spatially dependent partial differential equation (PDE) model was fitted to the FRAP data in order to estimate levels of diffusivity and the on and off rates from the mitochondria in lit and unlit states. Let *c*(*x*, *t*) be the concentration of unbleached protein in the cytoplasm and *m*(*x*, *t*) be the concentration of unbleached protein bound to the mitochondria. The PDE is then given by:
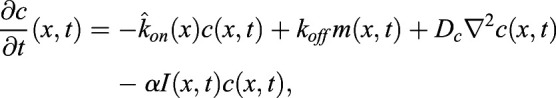

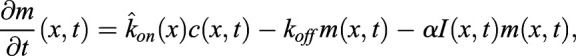
where 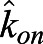 gives the binding rate of unbound protein in the cytoplasm to binding partners on the mitochondrial surface and *k*_*off*_ gives the corresponding dissociation rate, whilst *D*_c_ gives the rate of diffusion of unbound protein in the cytoplasm. The parameter *α* is a measure of the sensitivity of the fluorescent probe to photobleaching, and *I*(*x*, *t*) is the intensity of the laser light used to bleach and image the sample. Boundary conditions are given by:
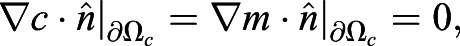
i.e. there is zero flux of protein from the mitochondria or cytoplasm across the nuclear membrane.

#### Model fitting

A numerical simulation of the FRAP experiment was implemented in MATLAB. For each cell, the numerical model was fitted to the data using a quasi-Newton method (the fminunc function of MATLAB) or interior point optimisation (the fmincon function of MATLAB). This process yields estimates of the diffusivity of free protein in the cytoplasm, and the forward and reverse rates of reaction with mitochondrial binding sites. From the reaction rates, the mass fraction of protein which is bound at equilibrium can be calculated.

### FLIP imaging

Cells were plated and cultured overnight in glass-bottomed MatTek dishes. Cells were transfected, and then were imaged 24–48 h after DNA transfection. One hour prior to imaging, the medium was changed to Leibovitz L-15 medium (Thermo Fisher Scientific; 21083027) with 10% serum. The cells were subsequently imaged using a Zeiss LSM880 microscope equipped with an argon laser (Zeiss, Germany) and a 63× objective (Zeiss, α-Plan Apochromat 63×/1.46 NA oil Korr TIRF). For FLIP experiments, bleaching was performed using the laser at 100% capacity with a wavelength corresponding to the fluorophore excitation wavelength at a single square ROI of 8×8 pixels (4.4 mm^2^). All images were 12-bit and 256×256 pixels. Before photobleaching, three measurements of fluorescence were taken. The ROI was then photobleached between every frame for 2 s using maximum laser power. A series of 150 images were taken every 2 s for up to 5 min. For nucFLIP, the bleaching region was placed in the nucleus; for cytoFLIP, the bleaching region was placed in the cytoplasm.

### Optogenetics light activation

Cells were plated at low confluence and cultured overnight in glass-bottomed MatTek dishes. Cells were transfected, and then were imaged 24–48 h after DNA transfection. One hour prior to imaging, the medium was changed to Leibovitz L-15 medium with 10% serum and, depending on the FPs being used, supplemented with one or more of the following: MitoTracker Red (10,000×; M7513; Thermo Fisher Scientific), MitoTracker Deep Red (10,000×; M22426; Thermo Fisher Scientific) and DRAQ5 (5000×; 65-0880-92; Thermo Fisher Scientific). Half an hour prior to imaging, the medium was changed again to Leibovitz L-15 medium with 10% serum without the stains.

If only a single protein is being studied, we recommend using Venus, MitoTracker Red and DRAQ5. If the objective is to study multiple proteins, then we suggest using Venus, mCherry and, depending on the microscope, combinations of MitoTracker Red, MitoTracker Deep Red, DRAQ5 or H2B–mTurquoise excited with very low light.

Cells were selected for imaging based on having effective sequestration of the Zdk–FP to mitochondria and moderate levels of Zdk–FP expression. The cells were subsequently imaged with a Zeiss LSM880 microscope equipped with an argon laser (Zeiss, Germany) and a 40× objective (Zeiss, Plan Apochromat 40×/1.3 NA oil Korr DIC M27) with an environmental chamber set at 37°C. Optogenetic activation was performed using a 458 nm laser line at 1% laser power on the bleaching function with 50 iterations, selecting an area encompassing the whole cell. Before activation, five frames were imaged without the photoactivation/bleaching function. Then, 150 frames of release with photoactivation and 150 frames of recovery without photoactivation were acquired. Frames were acquired ∼4 s apart, as this corresponded to the approximate speed of light recovery of the LOV domain (Strickland et al., 2012, 2010). In order to simultaneously acquire two or three other channels, a beam splitter (458/514/561/633 nm) was used, which allows imaging in cyan, yellow, red and far red wavelengths compatible with light activation at 458 nm. Nuclei were imaged with very low 458 nm laser power, which did not interfere with light activation, with emission captured at 463–486 nm. mCherry fusions were excited with 561 nm laser light and emission typically captured at 580–615 nm, except in the case of the double Venus and mCherry experiments, when a longer 615–633 nm emission range was captured. Venus fusions were excited with 514 nm laser light, and emission typically captured at 519–553 nm, except in the case of the double Venus and mCherry experiments when a shorter 517–535 nm emission range was captured. The double opto-release experiments used a shorter emission range for Venus and longer range for mCherry to avoid erroneous ‘bleed-through’ signal between channels. Mitotracker Red and Mitotracker Far Red were imaged using 561 nm and 633 nm excitation, respectively, and typically 579–624 nm and 714–759 nm emission windows, respectively. DRAQ5 was imaged using 633 nm excitation and a 677–742 nm emission window.

### 3D optogenetics light activation

All the experiments in 3D were performed on a lattice light-sheet microscope (LLSM) in the Advanced Imaging Center at the Howard Hughes Medical Institute, Janelia Research Campus. HaCaT cells were seeded on uncoated coverslips (CS-5R; Warner Instruments) in a 30 µl droplet, with 10,000–30,000 cells/droplet, and left to settle for 1 h before filling the plate well with medium. Cells were transfected as for the 2D condition experiments.

The LLSM system was configured and operated as previously described (Chen et al., 2014). Samples were kept at 37°C and illuminated using a 445 nm laser (Oxxius diode laser; initial laser power 132 mW) at 100% acousto-optic tunable filter transmittance, a 560 nm laser (MPB fibre laser, rated 500 mW) at 100% acousto-optic tunable filter transmittance and a 642 nm laser (MPB fibre laser, rated 500 mW) at 10% acousto-optic tunable filter transmittance. The excitation objective was a Special Optics 0.65 NA lens and the detection objective was a Nikon CFI Apo LWD 25× water dipping, 63× magnification, 1.1 NA. The signal was detected on two Hamamatsu Orca Flash 4.0 v2 sCMOS cameras. To separate the signal between two cameras, for the channel detecting MitoTracker DeepRed we used camera A with a BLPO-647R-25 long band-pass filter. For camera B we used an NF03-t42E-25 Notch filter, a BLP01-458R-25 long-pass filter and an FF01-465/537/623-25 beam splitter. Signal was split with a dichroic mirror (FF640-FDi014) to detect H2B–mTurquoise and mCherry. For the release phase, 80 frames were acquired with the 445 nm laser on, and in the recovery phase, four cycles of 19 frames without the 445 nm laser and one frame with the 445 nm laser were imaged (‘staggered release’). In addition to providing novel information about protein dynamics, staggered release can be used to map the position of the nucleus. The software used to operate the instrument and collect data was LabView (National Instruments). FIJI (https://fiji.sc/) was used for the quantification of fluorescence intensity in different ROIs. FIJI was also used to convert files into .h5 format for subsequent 3D visualisation and the generation of movies using Imaris (Bitplane).

### Immunofluorescence quantification for nuclear-to-cytoplasmic ratios

For quantification of the subcellular localisation of each protein of interest, the nuclear-to-cytoplasmic ratio was calculated manually. For each cell, a single square ROI of 8×8 pixels was placed in the nucleus and cytoplasm. ROIs were always selected in the DAPI channel of immunofluorescence images to avoid bias. The cytoplasmic ROI was then confirmed to be in the correct cell by checking the actin channel. The signal in the nuclear ROI was divided by the signal in the cytoplasmic ROI to derive nuclear-to-cytoplasmic ratio.

### Regional change in cytoplasmic intensity

To calculate a spatial map of the relative change in fluorescence intensity caused by blue light illumination, the average intensity of the five frames before blue light illumination and the average intensity of the five frames after 200 s of blue light illumination were calculated. The latter was then multiplied by 256 and divided by the former, with the resulting image presented in 8 bit. The image was also binned by a factor of two to reduce noise.

### FLIP quantification

For quantification of nuclear import and export using FLIP experiments, the nuclear and cytoplasmic compartments were determined manually in MATLAB using the roipoly command (Image Processing toolbox), and the total intensity of each compartment over time was extracted. Two control ROIs (one in the nucleus and one in the cytoplasm of one control cell) and one ROI outside any cells (to measure the background intensity) were measured. To normalise, first the background intensity was subtracted from the total intensity in each compartment, and the results were then divided by the average intensity of the two control ROIs. A simpler differential equation model than that employed in Ege et al. (2018) was fitted to this dynamic intensity data, including only one-dimensional temporal data rather than spatial data. However, see the ‘Mathematical derivation of the FRAP and optogenetics models’ section of the Materials and Methods for illustration of how such simpler models of import and export can be derived from the more complex set of PDEs. We fitted the normalised total intensities using a system of ODEs given by:
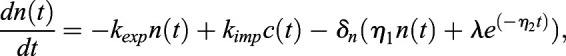

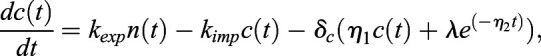

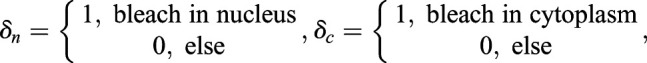
where *n*(*t*) and *c*(*t*) give, respectively, the total nuclear and cytoplasmic intensities at time, *t*. The parameter *k*_*exp*_ gives the export rate from the nucleus to the cytoplasm, and *k*_*imp*_ gives the import rate from the cytoplasm to the nucleus. The final functions correspond to intensity decay due to bleaching, depending on whether the bleaching occurs in the nucleus or cytoplasm. A single decay parameter *η*_1_ tends to underestimate the rapid initial bleaching, so a decaying exponential function was incorporated into the ODEs such that the bleaching is effectively modelled by a double exponential, one with a rapid rate and the other with a slower rate. Initial conditions are given by *n*(0)=*n*_0_ and *c*(0)=*c*_0_. At steady state, prior to bleaching:
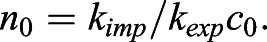
An analytic solution to the ODEs exists; however, we estimated parameters by fitting a numerical solution of the model to the experimental intensity data for each cell. Parameter estimates for *k*_*exp*_,  *k*_*imp*_,  *n*_0_,  *c*_0_,  *η*_1_,  *η*_2_ and *λ* were all required. We could reduce the number of parameters to fit by taking advantage of the steady-state relationship given above to fix the initial nuclear intensity in terms of import and export rate constants and initial cytoplasmic intensity. To optimise parameter fitting, we used the nonlinear model fitting function nlinfit in MATLAB's Statistics and Machine Learning toolbox. Initial guesses for parameters in the nlinfit function were given by the initial cytoplasmic normalised intensity for *c*_0_ and initial guesses of order 10^−3^ [based on population intensity analysis carried out in Ege et al. (2018)] for *k*_*exp*_ and *k*_*imp*_. To generate initial guesses for the bleaching decay rate constants we fitted a double exponential function:
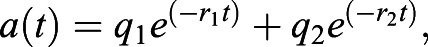
to the total normalised intensity of the entire cell. Initial guesses to this double exponential fit were given as 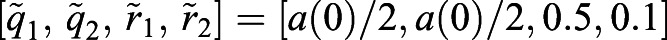. Differentiating this double exponential, *a*(*t*), into the form:
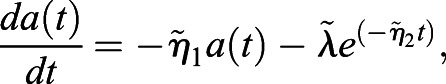
provided initial guesses for *η*_1_, *η*_2_ and *λ*.

### Optogenetic quantification

#### Opto-analyser – a cell compartment segmentation and model fitting app

To segment cellular compartments and extract dynamic intensities of the mitochondria, cytoplasm and nucleus we developed an app in MATLAB. The labelling of the nucleus and mitochondria alongside the FP labelling of the protein of interest allow temporal segmentation of each compartment. Appropriate compartment threshold levels for segmentation differ from frame to frame, and in some frames the signal from a given compartment is weak. Therefore, a moving percentile window takes a percentile projection of intensities over all frames in that window. Thresholding is carried out on these intensity projections in order to segment the compartments. This enables some of the morphological changes that may occur during the image acquisition period to be captured while still being robust to high levels of noise and low levels of signal. Using a percentile projection over all frames in the movie is obviously most robust to high noise and low signal but offers no sensitivity to morphological changes. Decreasing the number of frames in the moving percentile leads to greater sensitivity to dynamic compartment movement within the movie but reduces robustness in segmentation due to the projections being taken from fewer frames in each window. In the analysis carried out here, a moving percentile projection over ∼150 frames was applied, half of the total movie length.

The percentiles and size of moving window are selected by the user in the app. In order to provide appropriate segmentation for the moving projections, the images are manually processed at a number of equidistant points along the movie, known as seeding points, and these seeding points are interpolated to provide image masks and threshold levels for each compartment in each frame. The number of seeding points selected to manually process determines the size of window used for moving percentile projections (the number of seeding points and corresponding window size selection are referred to from hereon as a window profile). For example, for a movie of 100 frames, a window profile with two seeding points has a corresponding moving percentile window 50 frames long. The manually segmented seeding points will occur at frames 25 and 75, being percentile projections of frames 1–50 and 51–100, respectively. Threshold levels and image masks between frames 25 and 75 are interpolated, whilst they are fixed at the manually selected levels for frames 1–25 and 75–100. These interpolated thresholds are then applied frame by frame to the moving percentile intensity projections. Segmentation steps of the nuclear and mitochondrial compartments rely on the nuclear and mitochondrial channels only. Segmentation of the whole cell boundary relies on the generation of a hybrid channel where each frame is the maximum intensity projection of the nuclear, mitochondrial and protein channels in that frame. This makes the segmentation more robust to the heterogeneous and potentially weak intensity of the protein channel. In our analysis, we used a window length equal to half the total number of frames.

The app was developed in MATLAB, using its inbuilt App Designer software, and requires the Image Processing toolbox, the Statistics and Machine Learning toolbox and lsmread from https://github.com/joe-of-all-trades/lsmread. The app and associated documentation are available at https://github.com/RobertPJenkins/opto_analyser. The app incorporates both signal extraction via image segmentation and model fitting to the extracted signals. The app user interface is composed of three main panels. The left-hand panel is for initial user input and error message output (Fig. S2A). The central panel is composed of multiple tabs where the majority of image processing and model fitting takes place, with each tab covering a distinct aspect of image segmentation or model fitting (Fig. S2A). The right-hand panel aids image segmentation by illustrating the signal output and image processing parameter values for each window profile that has been completely or partially processed (Fig. S2A).

Different window profiles (i.e. different numbers of manually segmented seeding frames and moving window lengths) can be analysed to observe the effects of length of moving window on signal robustness and ability to capture compartment motility. For each window profile, the intensity projections of the nuclear, mitochondrial and hybrid channels are determined, and segmentation of each manual seeding point within that window profile is then iteratively carried out (Fig. S2A). Overlaid image masks and output compartment intensity values are updated in real time to aid the user with correct segmentation of a seeding point by allowing the user to determine the most appropriate compartment thresholds not only from the current manually thresholded image, but also from the extracted population intensity profiles and percentile and threshold levels for previously processed seeding points and window profiles. Population intensities of all previously extracted window profiles and seeding points provide real-time information on the effects of length of moving window and segmentation options on the resulting dynamic intensity profiles for each compartment (Fig. S2A).

Once the user has carried out the relevant processing for all seeding points over all window profiles, then threshold levels and image masks are interpolated and applied to each frame of the relevant moving percentile window to extract intensity signals for each compartment. The user then has the option of carrying out model fitting to the extracted signals. Background subtraction and compartment intensity normalisation (to the proportion of total intensity in a cell, see below) are carried out before the ODEs are fitted to extracted signals for all window profiles (Fig. S2A).

#### Background subtraction and bleaching intensity normalisation

Intensity normalisation consists of two stages: (1) background subtraction and (2) normalisation due to bleaching. Background subtraction is carried out for each frame. The mean of a user-defined ROI away from any cell is calculated over time and subtracted from the compartment intensities. A small number of movies were too confluent to enable this step.

The intensities are then normalised to account for laser bleaching. The level of laser bleaching is determined from the behaviour of the total intensity of the cell over time, which appears to differ between release and recovery phases, presumably due to the additional effect of the blue light. Laser bleaching is approximately linear in each phase, and so total intensity is estimated by a bilinear fit to the release and recovery phases to remove noise. The intensity in each compartment is then normalised by dividing by this bilinear total intensity. This results in the units we fit to being the total cellular proportion of the protein of interest in each compartment. We assume that the total cellular proportion of the protein of interest is one for all time (i.e. intensity is conserved). The linear fits in each phase are made on data far from the initial release or initial recovery to reduce the effects of possibly greater levels of noise in these stages.

#### Mathematical model

As in the FLIP analysis, we fitted a system of ODEs (see Materials and Methods section ‘Mathematical derivation of the FRAP and optogenetic models’) to the extracted population intensity data for the mitochondrial, cytoplasmic and nuclear compartments. The ODEs are given by:
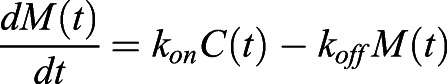

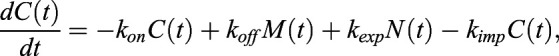

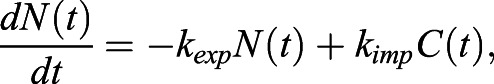
where *M*(*t*), *C*(*t*) and *N*(*t*) are the mitochondrial, cytoplasmic and nuclear intensities, respectively, at time, *t*. The initial conditions are given by *M*(0)=*M*_0_, *C*(0)=*C*_0_ and *N*(0)=*N*_0_. The rate of export from the nucleus to the cytoplasm and rate of import from the cytoplasm to the nucleus are given by *k*_*exp*_ and *k*_*imp*_, respectively. The parameter *k*_*on*_ gives the rate at which unbound protein in the cytoplasm moves through the cytoplasm and binds to molecules on the surface of the mitochondria, and the parameter *k*_*off*_ gives the rate at which protein dissociates from the surface molecules of the mitochondria and enters the cytoplasmic compartment. These values are dependent on whether the system is in the lit or unlit state i.e.:
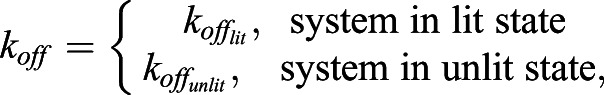

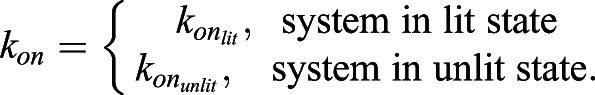


#### Model fitting

The system of ODEs is fitted to the experimental data using MATLAB's nlinfit function from the Statistics and Machine Learning toolbox. The fitting weights of each compartment are normalised such that each compartment contributes an equal amount to the residuals. Rate constants that require fitting are *k*_*exp*_, *k*_*imp*_, 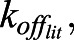 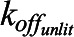 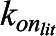 and 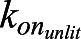. The model is fitted such that the parameters 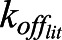 and 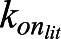
are fitted only during the lit (release) phase and 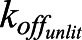 and 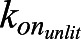
are only fitted at the unlit initial steady-state phase and the recovery phase. In reality, when the cell is being illuminated, it is illuminated only for a fraction of each frame. The fitting of models with oscillatory release and recovery rates was investigated, but it was found that this led to a very ‘lumpy’ parameter space consisting of a large number of pockets of local minima, as described in Pitt and Banga (2019). This was compounded by the oscillations taking place within a single frame, resulting in minimal oscillatory information. As such we fitted a model where the illumination was assumed to be continuously on during the lit phase.

The initial conditions, *M*_0_, *C*_0_ and *N*_0_, need to be treated as fitted parameters rather than fixed at initial experimental levels due to experimental noise. The number of free parameters can be reduced by taking advantage of the steady-state relationships 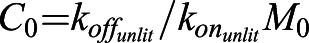 and *N*_0_=*k*_*imp*_/*k*_*exp*_*C*_0_, allowing us to fix the initial nuclear and cytoplasmic intensities in terms of other parameters. As initial guesses for our parameters we take 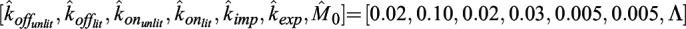, where initial guesses for release and recovery rates are taken from the results of the relevant FRAP experiments and where Λ gives the median mitochondrial intensity over the first five unlit frames prior to release.

#### Model fitting quality assessment

Various models were fitted to the resulting signal, and quality of fit was compared using the Akaike information criterion (AIC). A model with a single recovery rate constant throughout the experiment was compared to a model with different recovery rates based on lit or unlit state. For these models we compared cases where all parameters were free to cases where the non-illuminated release and recovery rates were fixed independently and to cases where the ratio of non-illuminated release and recovery was fixed. Fixed values were determined according to equivalent FRAP median values (Fig. 2). Models with parameters fixed at FRAP levels generally performed poorly, whilst the case of two release and two recovery rates with all parameters free generally had the highest AIC probability, and this was the model selected for parameter fitting. This revealed that when using two free on rates, 77.6% of all fits had an AIC probability >0.9, which compared very favourably with using a single free on rate, which yielded only 12.9% of fits having an AIC probability >0.9. In the two models with all rate constants being free, the initial guesses for release and recovery rate constants were chosen from the equivalent median values of the FRAP data. The resulting parameter fits showed strong agreement with the FRAP data (Figs 1, 2), ruling out the need to consider model fitting approaches where release and recovery rates were bounded within realistic ranges according to FRAP.

### Mathematical derivation of the FRAP and optogenetic models

#### Introduction to the full protein interaction and transport model

To begin, we introduce the basic protein dynamics model from which we derive both the FRAP model and the optogenetics model. The cell is modelled as comprising two spatial compartments: the cytoplasm, Ω_c_, and the nucleus, Ω_*n*_; the boundary of the cytoplasm is denoted by ∂Ω_*c*_, the nuclear membrane by Γ and the cell membrane by 
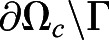
. Let *x*=ℝ^*d*^ denote spatial coordinates and *t*=ℝ_≥0_ denote time. The full system is described by *d*=3; however, for consistency with 2D experimental data we consider only *d*=2.

We assume that a protein of interest undergoes immobilising reaction with binding partner molecules localised to the surface of the mitochondria. By assumption, these reactions are dominant so that all other interactions in the cytoplasm may be neglected. Let *c* denote the concentration of unbound protein in the cytoplasm, *s*_*m*_ the concentration of molecules on the mitochondrial surface that can bind with the protein, *b* the concentration of protein–mitochondrial complexes and *n* the concentration of protein in the nucleus. The governing equations are:(1)
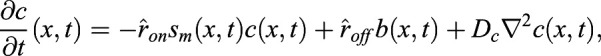
(2)
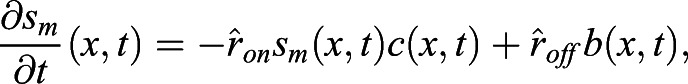
(3)
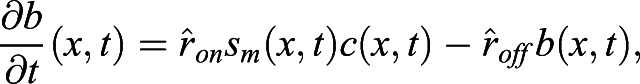
within the cytoplasm (*x*∈Ω_c_), and:(4)
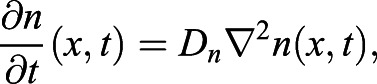
within the nuclear compartment (*x*∈Ω_*n*_). The parameter 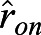 is the rate (per unit concentration) of association between the protein of interest and binding sites on the mitochondria; 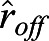 is the rate of dissociation of the protein of interest from the mitochondrial binding sites; *D*_*c*_ and *D*_*n*_ are the diffusivities of the protein of interest in the cytoplasm and nucleus, respectively.

We assume that the flux of protein across the nuclear membrane is linearly proportional to local concentration, so that:(5)
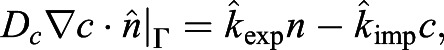
where 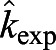 and 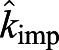 denote export and import rates, respectively. Likewise:(6)
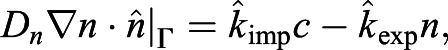
where 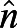 is the outward unit normal to Γ in each case. We also assume no flux of protein across the cell membrane, such that:(7)
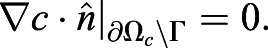
It is useful to introduce the following quantities. The total masses of each type of molecule in the cytoplasm are:(8)
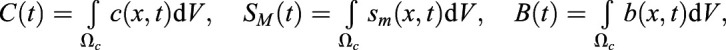
and likewise the total mass of protein in the nucleus is:(9)
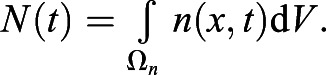
The nuclear and cytoplasmic volumes, |Ω_*c*_| and |Ω_*n*_|, respectively, and the surface area of the nucleus, |Γ|, are defined by:(10)
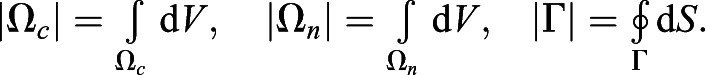


#### Non-dimensionalisation

Here we introduce the dimensionless variables *t*^′^ and *x*^′^:(11)
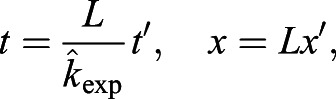
where *L* denotes the ‘characteristic length scale’ of the cell. We may define *L* as the diameter of the cell (i.e. the greatest straight-line distance between any two points contained within the cell). In dimensionless form, the cytoplasmic protein localisation model (Eqns 1–3) is then:(12)
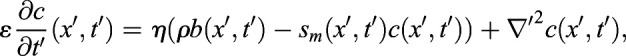
(13)
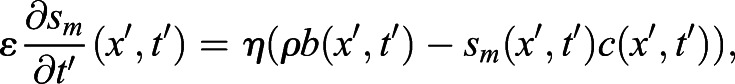
(14)
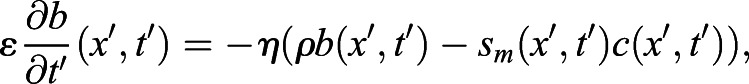
where the dimensionless parameters ε, *η* and *ρ* are defined as:(15)
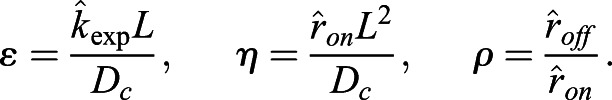
The boundary flux condition (Eqn 5) becomes:(16)
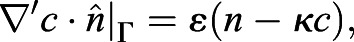
where:(17)
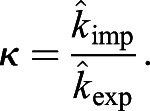
Similarly, for the nuclear-localised protein (Eqn 4):(18)
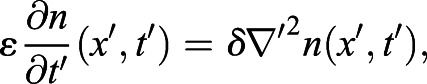
where:(19)
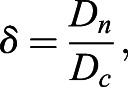
and with boundary condition (Eqn 6) becoming:(20)
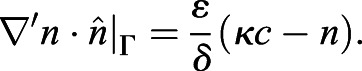


#### Asymptotic limit (fast diffusion, slow translocation)

If diffusion is much more rapid than translocation between compartments, then ε≪1. We may investigate this case in approximation by taking the limit ε→0, so that the system (Eqns 12–14) is reduced to the equilibrium equations:(21)
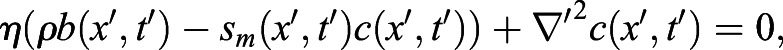
(22)
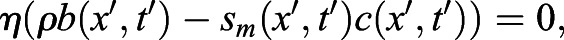
along with the boundary conditions (see Eqn 16):(23)
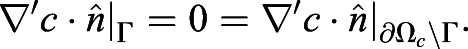
The latter implies that there is no flux of protein across the cytoplasmic boundary:(24)
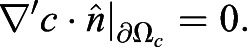
Assuming that the ratio of nuclear to cytoplasmic diffusivity remains finite (i.e. we do not have δ→0 in Eqn 18):(25)
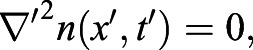
along with the boundary condition (see Eqn 20):(26)
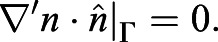


The above requires that free cytoplasmic protein, *c*, is spatially homogeneous at equilibrium. However, this fact is not necessarily intuitively obvious, given that the mitochondrial binding sites are non-homogeneously distributed, and so across space varying numbers of molecules are released and captured per second. Since we rely on the spatial homogeneity of *c* to derive the optogenetics model, we include here a brief proof.

By subtracting the two equilibrium equations (Eqns 21, 22), we see that *c* follows Laplace's equation:(27)
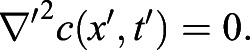
Multiplying the boundary condition (Eqn 24) by *c* gives:(28)
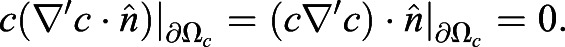
Let *J* be defined as the integral:(29)
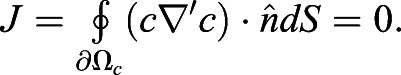
Then, by the divergence theorem:(30)
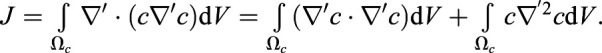
Since *J*=0 and 
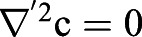
:(31)
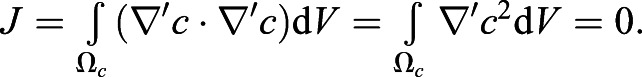


As 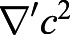 is non-negative, *J*=0 requires 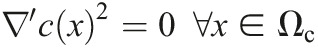 which implies that 
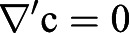
everywhere, or equivalently that *c* is spatially uniform, as required. An almost identical argument may be made to show that *n* is also spatially uniform.

#### Short timescale preamble for the FRAP model

Ideally, FRAP experiments are conducted over a sufficiently short timescale that translocation is negligible and diffusion (and possibly interactions at the mitochondria) may be quantified. In other words, the ideal FRAP experiment is conducted over a short timescale of order ε. To investigate this case, we set *t*^′^=ε*τ*, yielding from Eqn 12:(32)
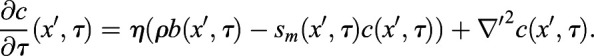


Noting that 
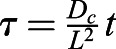
 (see Eqns 11, 15), we can at this stage return to dimensional measurements to obtain the final FRAP model:(33)
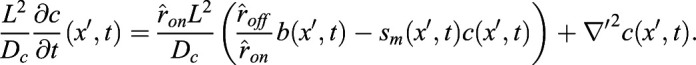
Substituting 
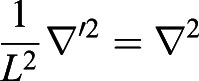
 leaves:(34)
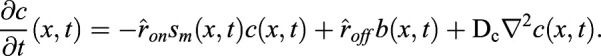
Likewise, for *s*_*m*_ and *b*:(35)
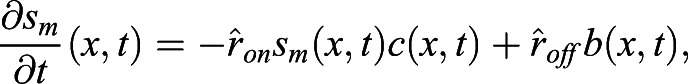
(36)
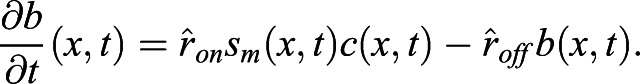


#### FRAP model

We can now derive the FRAP model from the system of Eqns 34–36. We assume that, prior to the beginning of the FRAP experiment, the protein of interest has already reached equilibrium; *c*=*c*_eq_, *s*_*m*_=*s*_*m*eq_ (*x*), *b*=*b*_eq_(*x*), where the concentration of binding sites, *s*_*m*_, and the concentration of bound protein, *b*, are not spatially uniform in general.

Let *c*_*f*_ (*x*, *t*) be the concentration of observable unbleached protein in the cytoplasm [i.e. fluorescent *c* (*x*, *t*)], and *m*_*f*_ (*x*, *t*) be the concentration of observable unbleached protein bound to the mitochondria [i.e. fluorescent *b*(*x*, *t*)]. It directly follows that:(37)
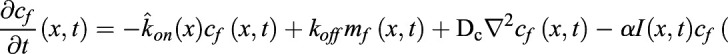
(38)
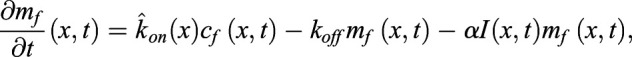
where 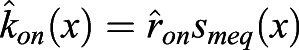 and 
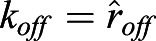
. By assumption, photobleaching neither alters the concentration of the protein of interest in the cytoplasm, nor its chemical properties. Hence the equilibrium state is preserved throughout the FRAP experiment. The parameter *α* is a measure of the sensitivity of the fluorescent probe to photobleaching, and *I*(*x*, *t*) is the intensity of the laser light used to bleach and image the sample (note the assumption that photobleaching is a linear first-order process).

The boundary conditions are:(39)
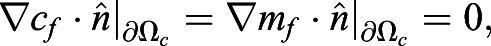
where the condition on *c*_*f*_ follows from the result of the section ‘Asymptotic limit (fast diffusion, slow translocation)’ (see Eqn 24), and the condition of *m*_*f*_ follows directly from the assumptions of the protein translocation model. It is this set of equations that is described in the ‘FRAP imaging’ section of the Materials and Methods with subscripts removed for brevity.

As there is negligible translocation of the protein of interest between nucleus and cytoplasm on the short timescale and the FRAP experiment is conducted in the cytoplasm, there is no need to include the nuclear-localised protein in the model.

#### Long timescale preamble for the optogenetic model

If the cell is observed over a sufficiently long timescale, then the effect of translocation between the nucleus and cytoplasm becomes significant. On this longer timescale, diffusion drives the free protein fractions towards quasi-equilibrium in both the nucleus and cytoplasm so that:(40)
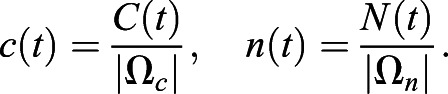
We begin by integrating the initial model (Eqns 1–4), starting with:(41)
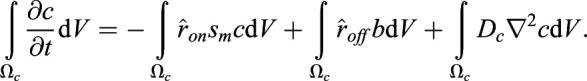
The divergence theorem implies that:(42)
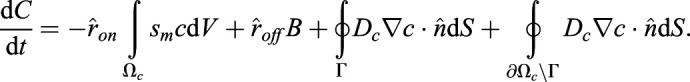
The boundary conditions (Eqn 5) may be used to evaluate the surface integral, together with the homogeneity of *c* and *n* to obtain:(43)
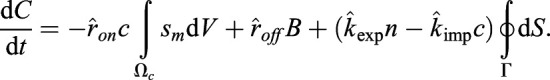
By extension (see Eqn 10):(44)
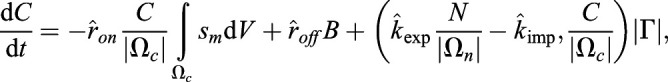
and we finally end up with:(45)
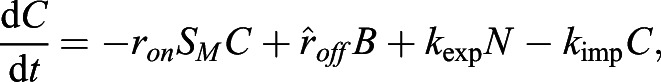
where 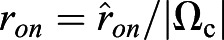, 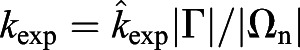 and 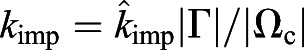. Using similar methods, we find that:(46)
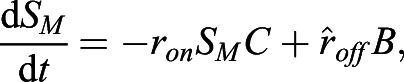
(47)
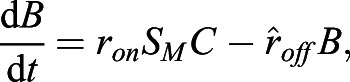
(48)
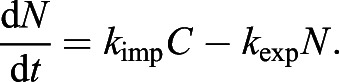


#### Optogenetics model

With fluorescence microscopy, only the fluorescent fractions of *C*, *B* and *N* are observed. Let *C*_*F*_ be the mass of free fluorescent protein in the cytoplasm, *M*_*F*_ be the mass of fluorescent protein bound to the mitochondria and *N*_*F*_ be the mass of free protein in the nucleus. It follows directly from Eqns 45–48 that:(49)
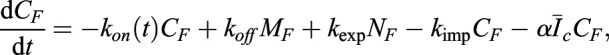
(50)
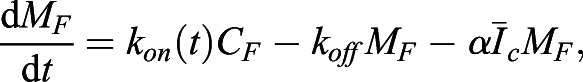
(51)
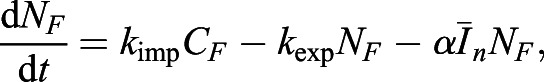
where 
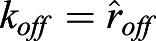
 is the dissociation rate and:(52)
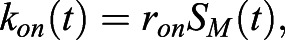
is the association rate. The terms 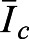 and 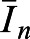 are the average laser light intensity across the cytoplasm and nucleus, respectively:(53)
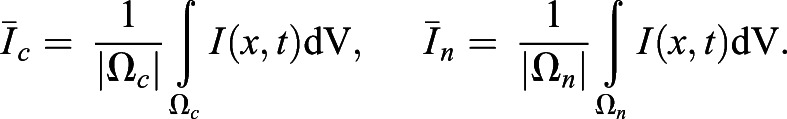


These terms account for the small amount of photobleaching caused by the imaging laser. If the data are normalised to account for fluorescence signal loss during the course of the experiment, then these photobleaching terms are redundant and may be omitted. It is the model of Eqns 49–51 described in the ‘Optogenetic quantification’ section of the Materials and Methods. The subscripts highlighting that these proteins represent the fluorescent fractions have been omitted for brevity, and suitable prior normalisation results in the omission of the average laser light intensity functions.

#### The optogenetic forward reaction rate

Unlike FRAP, illumination in optogenetics alters the chemical properties of the protein of interest and so perturbs the equilibrium. Illumination destabilises the bonds between the protein and the mitochondria, increasing the rate of dissociation, 
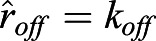
. The increase in free protein in the cytoplasm induces a net transfer of protein from the cytoplasm to the nucleus. Both of these processes increase the total mass of unoccupied mitochondrial binding sites, *S*_*M*_(*t*). In other words, as protein is transported out of the cytoplasm, competition for binding sites is decreased, and so the relative frequency of binding events per unit protein concentration increases.

This reduction in competition for binding sites tends to increase the forward rate of reaction, so we may expect FRAP experiments to show that the association rate is greater in the illuminated equilibrium state than in the unilluminated equilibrium state. Furthermore, in the optogenetics experiments, the cell transitions between these two equilibrium states as illumination intensity is varied. For this reason, the optogenetic forward reaction rate, *k*_*on*_(*t*), is technically a time-dependent function. However, if the mass of binding sites greatly exceeds the mass of protein, then *S*_*M*_(*t*) is approximately constant. Furthermore, if the affinity, *r*_*on*_, is unaffected by illumination, then the forward reaction, *k*_*on*_=*r*_*on*_*S*_*M*_, may be approximated as a constant.

## Supplementary Material

Supplementary information

Reviewer comments
