# Supplementary material for: An optogenetic method for interrogating YAP1 and TAZ nuclear–cytoplasmic shuttling
Source: J Cell Sci. 2021 Jul 9;134(13):jcs253484. doi: 10.1242/jcs.253484 (PMC8313864; doi:10.1242/jcs.253484)
Supplement: Supplementary information [file joces-134-253484-s1.pdf]

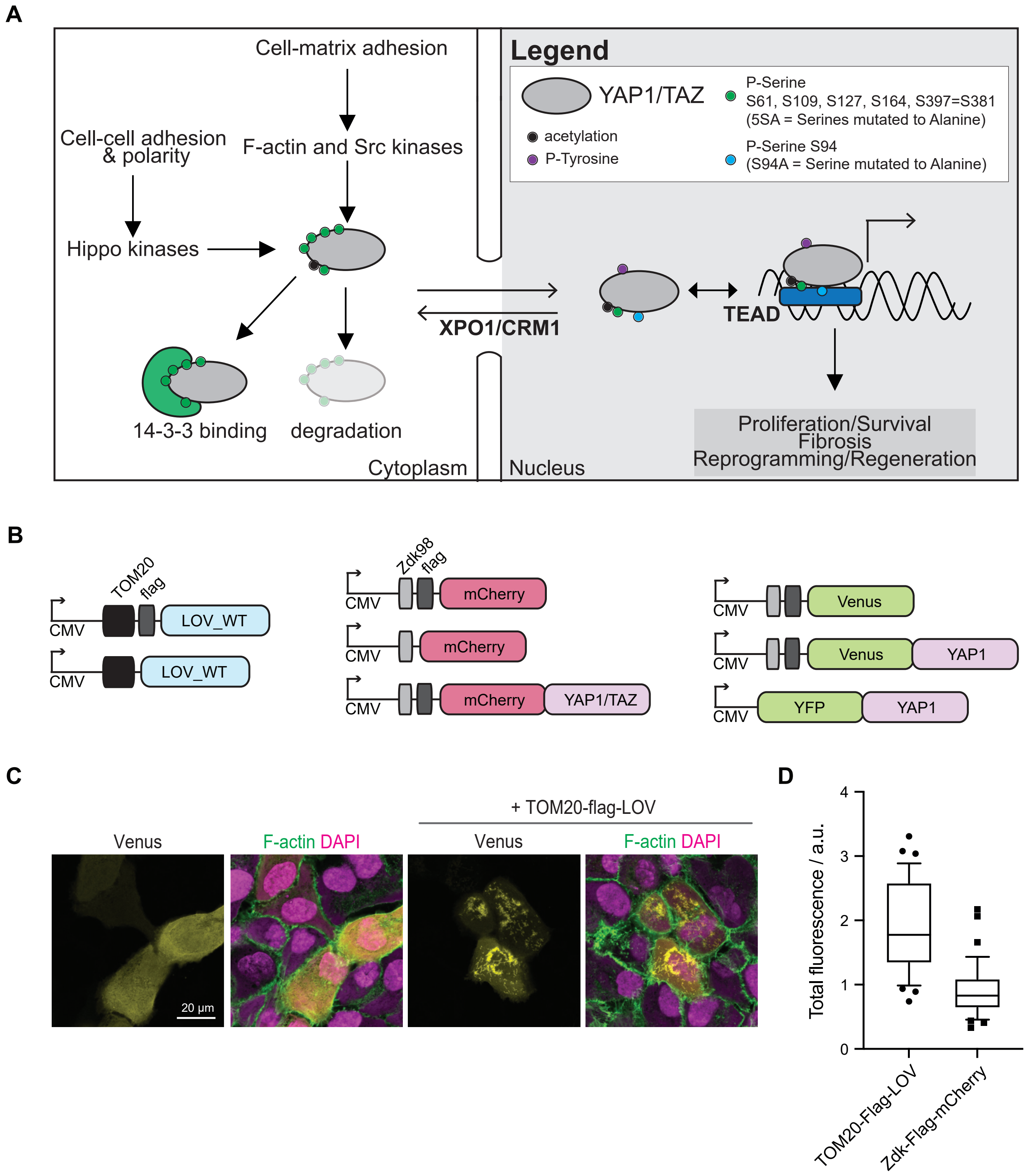


**Fig. S1. Related to Figure 1. Mitochondrial sequestration using TOM20-LOV.**

**(A)** Schematic of main known mechanisms of regulation of YAP1 and TAZ.

**(B)** Schematic of various constructs used in opto-release system. The isoform used for YAP1 is the human isoform 1-2$\gamma$. TOM20, mitochondrial anchor; Flag, flat-tag; LOV2, light-sensitive LOV domain; Zdk98, Zdk sequestering peptide; Venus/mCherry, fluorescent proteins.

**(C)** Representative pictures of HaCaT cells transiently transfected with Zdk-Flag-Venus (yellow) in the presence or absence of TOM20-Flag-LOV. Phalloidin staining to visualise F-actin (green) and DAPI to visualise nuclei (magenta) are also shown.

**(D)** Relative expression levels of sequestering construct TOM20-Flag-LOV compared to sequestered Zdk-Flag-mCherry quantified by anti-Flag-tag staining total fluorescence intensity in transiently transfected HaCaT cells; n>33 cells for each condition.

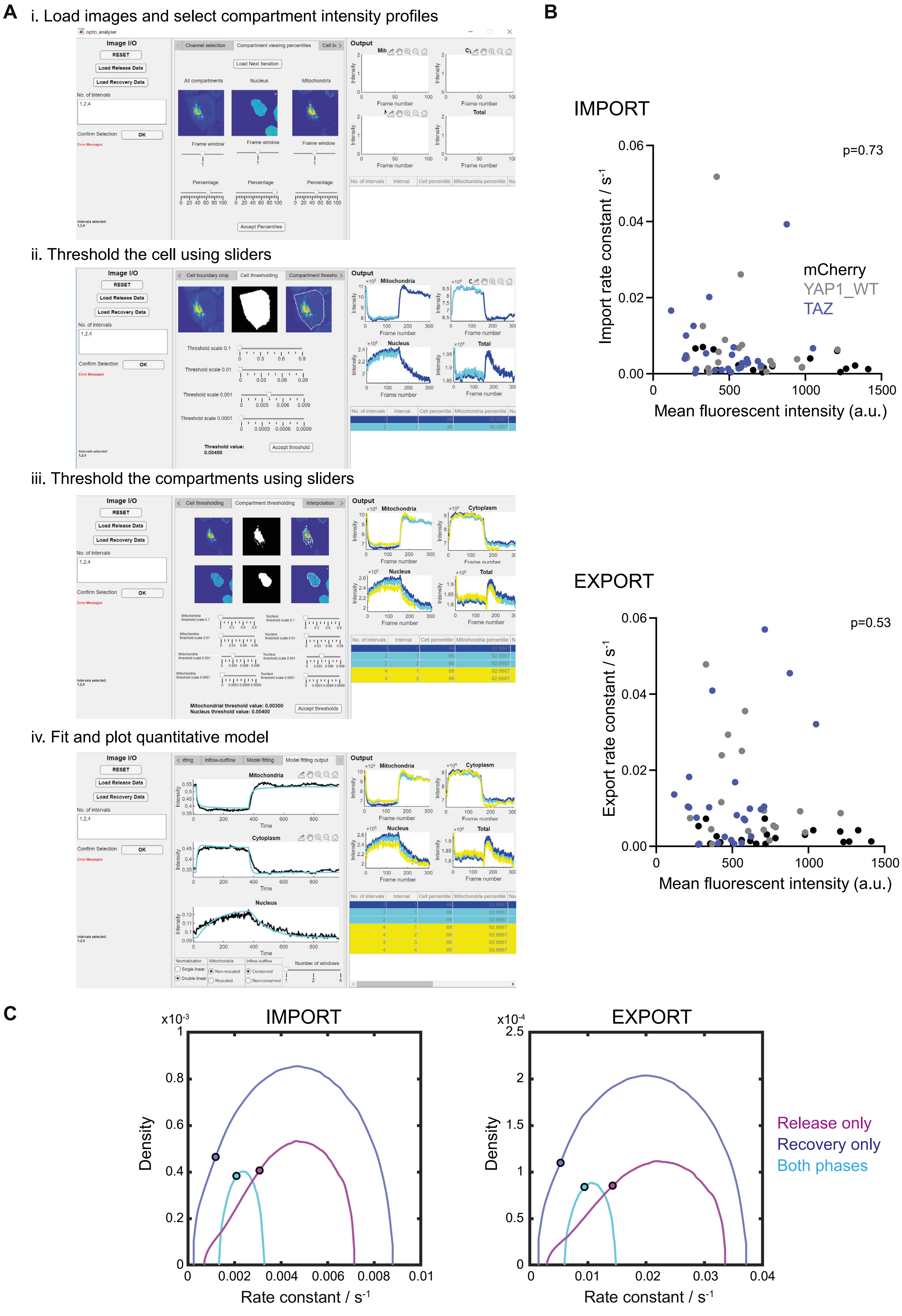


**Fig. S2. Related to Figure 2. MATLAB App for semi-automated cell compartment segmentation and rate constant fitting.**

**(A)** Screenshots demonstrating the MATLAB app; (i) loading data and specifying fluorescence/compartment channels and intensity projections; (ii)(iii) fluorescence intensity thresholding of the cell (ii) and cell compartments (iii) in real time using sliders; (iv) model fitting and rate derivation.

**(B)** Scatter plot showing mean fluorescence intensity of each cell plotted together with numerical value of import or export; p values indicate the likelihood of a significantly non-zero slope (Zdk-Flag-mCh, Zdk-Flag-mCh-YAP1: n=21; Zdk-Flag-mCh-TAZ n=26).

**(C)** Densities of import and export values that produce fits with sum of squares of error at most 10% greater than that of the global optimum when varying import and export simultaneously for a cell expressing a Zdk fusion protein (Zdk-Flag-mCh-YAP1) and subjected to imaging during both blue light illumination and cessation of blue light imaging. Cyan – release and recovery fit, magenta – release only fit and purple –recovery only fit. The dots show the location of optimum fits. All other parameter values were fixed at their global optimums.

Journal of Cell Science • Supplementary information

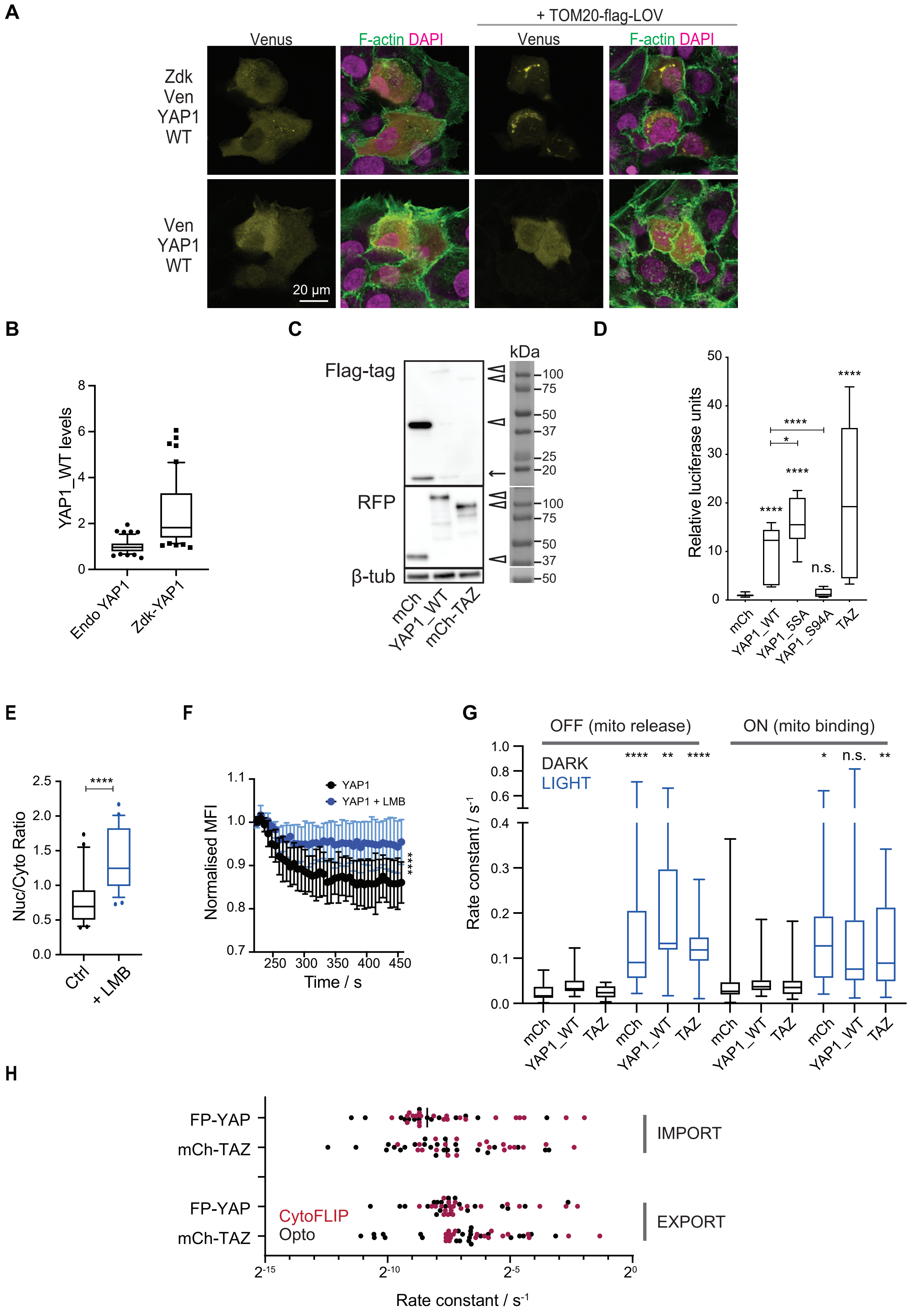


Journal of Cell Science • Supplementary information

**Fig. S3. Related to Figure 3. Functionality of YAP1 and TAZ constructs.** (A) Representative pictures of HaCaT cells transiently transfected with Zdk-Flag-Venus-YAP1_WT or Venus-YAP1_WT (yellow) in the presence or absence of TOM20-Flag-LOV. Phalloidin staining to visualise F-actin (green) and DAPI to visualise nuclei (magenta) are also shown. Note that mitochondrial sequestration only occurs in the presence of both Zdk-tagged and TOM20-Flag-LOV peptides.

**(B)** Boxplot (10-90 percentile, median) of relative Zdk-Flag-mCherry-YAP1_WT and endogenous YAP1 levels quantified by immunofluorescence staining intensity in transiently transfected HaCaT cells; n= 54 cells.

**(C)** Expression of unsequestered Zdk constructs. Western blot showing expression of Opto-release constructs stably expressed in HaCaT cell line. Empty arrowheads point to Opto-release protein (predicted molecular weight: Zdk-Flag-mCh 37.5kDa, Zdk-Flag-mCh-YAP1_WT 96kDa, Zdk-Flag-mCh- TAZ_WT 85kDa). An arrow points to a cleavage product. Anti-RFP antibody recognizes mCherry fluorescent protein. β-tubulin as loading control (42kDa) is also presented.

**(D)** Luciferase assay using TEAD-driven reporter (5xMCAT) for YAP1/TAZ activity in HaCaT cells following transient transfection with Zdk constructs driven by CMV promoters. Boxplot (10&90, median) represents three independent experiments, each with 3 technical replicates. Mann-Whitney U test, statistics above each bar represent comparison to mCH negative control.

**(E)** Boxplot (10&90, median) of nuclear-to-cytoplasmic ratio corresponding to DMSO (Control) and 20nM Leptomycin B (LMB) treated HaCaT cells transiently transfected with Zdk-Flag-mCherry-YAP1_WT; n>20 cells from two independent experiments.

**(F)** Mean ± SD of mean fluorescence intensity (MFI) of HaCaT cells transiently transfected with Zdk-Flag-Venus-YAP1_WT and treated with DMSO (Control) or 20nM Leptomycin B (LMB); n=7 cells from two independent experiments.

**(G)** Boxplot (min/max, median) of Zdk-Flag-mCherry, Zdk-Flag-mCherry-YAP1-WT and Zdk-Flag-mCherry-TAZ mitochondria off and on rate constants in presence and absence blue light illumination; n> 15 cells for each condition. * p<0.05, ** p<0.01, *** p<0.001, **** p<0.0001 (Kruskal-Wallis test with comparison between dark and light for each construct and between constructs in the dark and light).

**(H)** Comparison of import and export rate constants of Zdk-Flag-FP-YAP1_WT and Zdk-Flag-mCh-TAZ derived using opto-release (black dots) and cytoplasmic FLIP (red dots) methodologies. Graph shows individual values and median. Each dot represents one cell.

Journal of Cell Science • Supplementary information

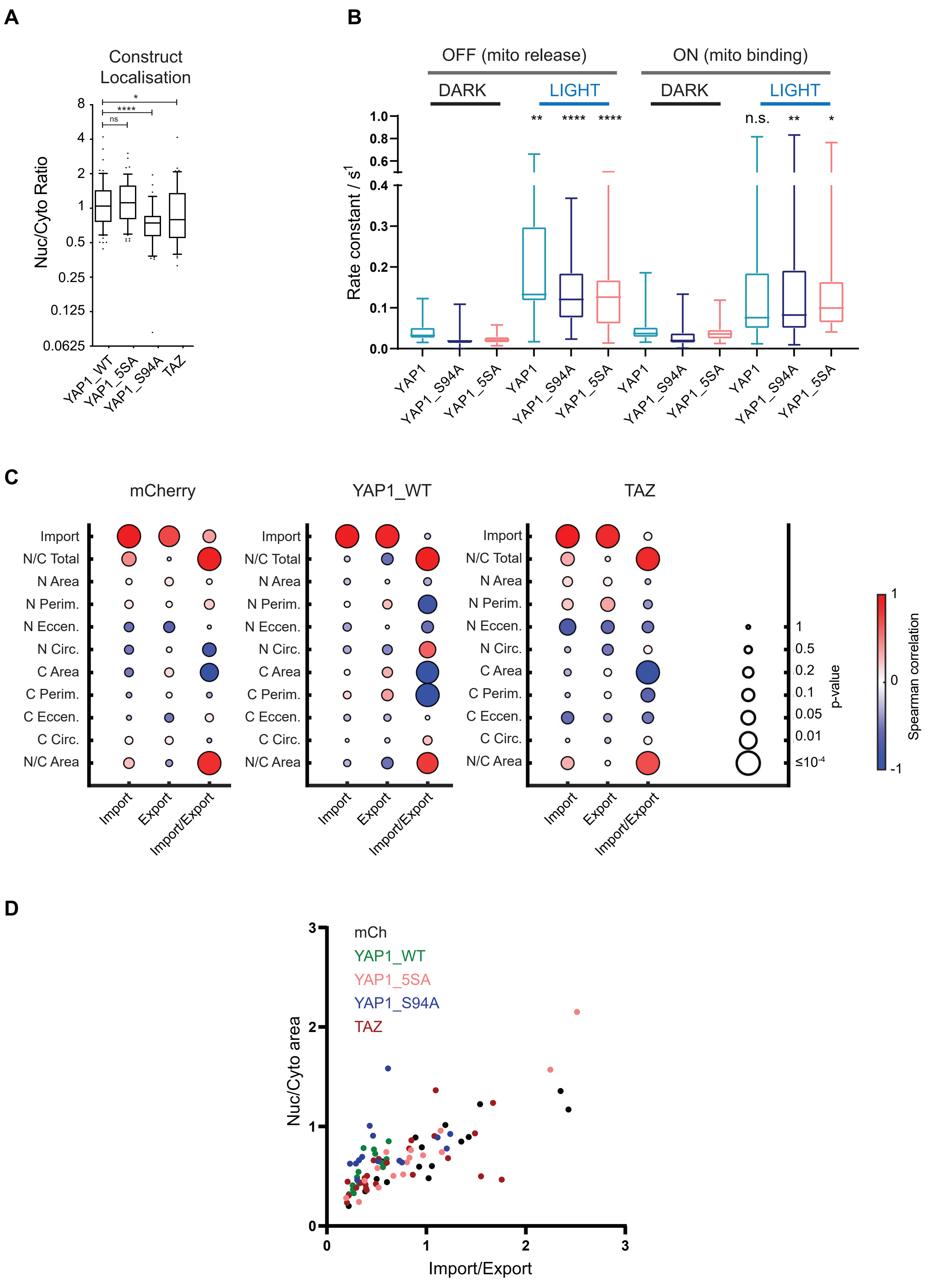


**Fig. S4. Related to Figure 4. YAP1 mutant parameters and correlations with cell morphology metrics.**

**(A)** Boxplot (10&90, median) of nuclear-to-cytoplasmic ratio (log2 scale) corresponding to three experimental repeats of HaCaT cells transiently transfected with CMV-driven Zdk-Flag-mCh constructs (n>29cells; n>20 for Taz, 2 experimental repeats). Mann-Whitney U test.

**(B)** Boxplot (10-90 percentile, median) of Zdk-Flag-mCherry-YAP1_WT, Zdk-Flag-mCherry-YAP1_5SA and Zdk-Flag-mCherry-YAP1_S94A mitochondria off and on rate constants in presence and absence blue light illumination; n> 15 cells for each condition. * p<0.05, ** p<0.01, ***p<0.001, ****p<0.0001 (Kruskal-Wallis test with comparison between dark and light for each construct and between constructs in the dark and light).

**(C)** Import, export and import/export ratio correlations with cell morphology, specified by area, perimeter, circularity and eccentricity (N – nucleus, C – cytoplasm), as well as nuclear-to-cytoplasmic ratio, import and nuclear-to-cytoplasmic area. Plots show Zdk-Flag-mCherry (left), Zdk-Flag-mCh-YAP1_WT (middle), and Zdk-Flag-mCh-TAZ (right). Circle colour reflects Spearman correlation (bright red +1, dark blue -1) and circle size the p-value of the correlation (large, significant; small, non-significant).

**(D)** Plot shows the relationship between Nuc/Cyto area and Import/Export rate constants for the different Zdk-Flag-mCh proteins. Legend indicates which colours refer to which proteins.

Journal of Cell Science • Supplementary information

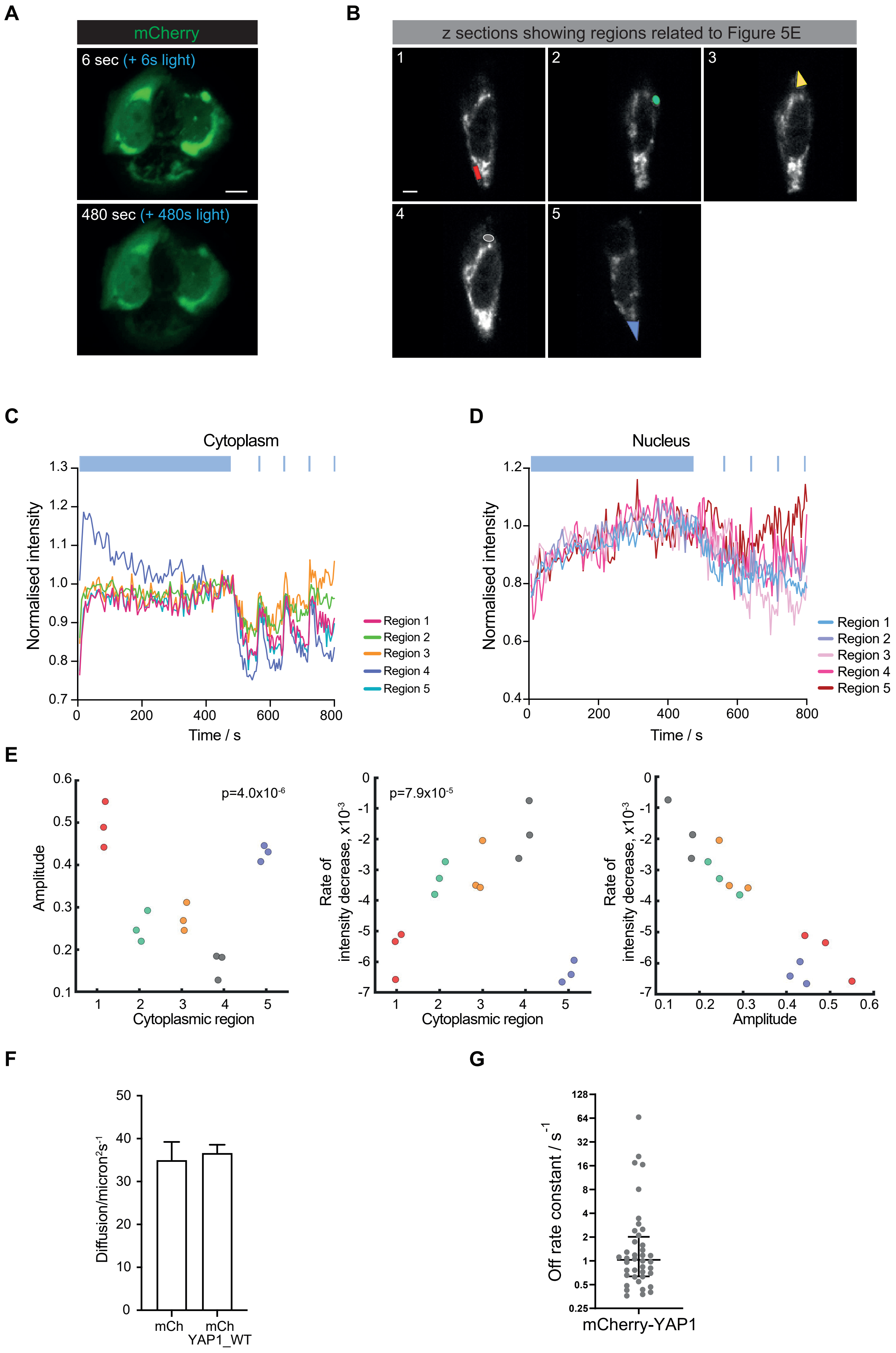


**Fig. S5. Related to Figure 5. Variable YAP1 cytoplasmic dynamics.**

**(A)** the regions shown in the top and bottom panel are different slices of the light-sheet image. Scale bar is 5μm.

**(B)** Numbered regions of the cell analysed for Figure 5E. Scale bar is 5μm.

**(C)** Quantification of intensities corresponding to different cytoplasmic regions during the 3D optogenetic experiment in another HaCaT cell expressing Zdk-Flag-mCherry-YAP1_WT (additional to Figure E).

**(D)** Quantification of intensities corresponding to different nuclear regions during the 3D optogenetic experiment in HaCaT cell expressing Zdk-Flag-mCherry-YAP1_WT.

**(E)** Scatter plots of peak amplitude and gradient of decrease from maximum value of each peak for each cytoplasmic region of data in Figure 6E (left and centre) and scatter plot of amplitude of peaks versus gradient of decrease (right). Peak amplitude approximated by maximum minus minimum signal value. For the gradient of decrease a linear fit was made to all data points after and including the peak value. The gradient provides an approximation to rate of decrease of the peak. Left and middle plots shows region-dependent variation in amplitude and decay of peaks following blue light pulses during the ‘recovery’ phase. Right plots shows inverse correlation between peak amplitude and decay. Statistical tests carried out using one-way ANOVA.

**(F)** Bar graph (median with 95% confidence intervals) of Zdk-Flag-mCh and Zdk-Flag-mCh-YAP1_WT diffusion rates. n=14 cytoplasmic regions for mCh and n=40 cytoplasmic regions for mCh-YAP1.

**(G)** Mitochondrial off rate constants for Zdk-Flag-mCh-YAP1_WT. Each dot represents a different cytoplasmic region (40 regions from 15 cells). Graph shows individual values and median with interquartile range.

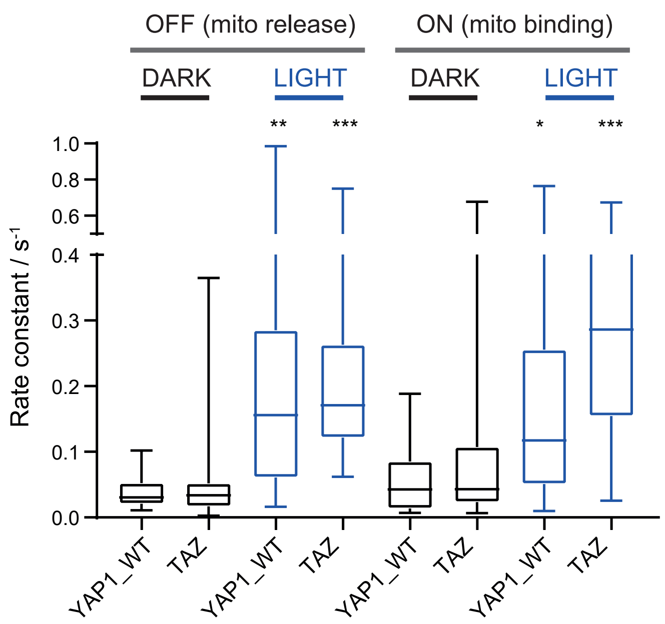


**Fig. 6. Related to Figure 6. Rates of YAP1 and TAZ in the same cell.** Boxplot (10-90 percentile, median) of Zdk-Flag-Venus-YAP1_WT and Zdk-Flag-mCherry-TAZ mitochondria off and on rate constants in presence and absence blue light illumination, measured in the same cell. n=18 cells from three experiments. * $p<0.05$, ** $p<0.01$, *** $p<0.001$ (Kruskal-Wallis test with comparison between dark and light for each construct and between constructs in the dark and light).

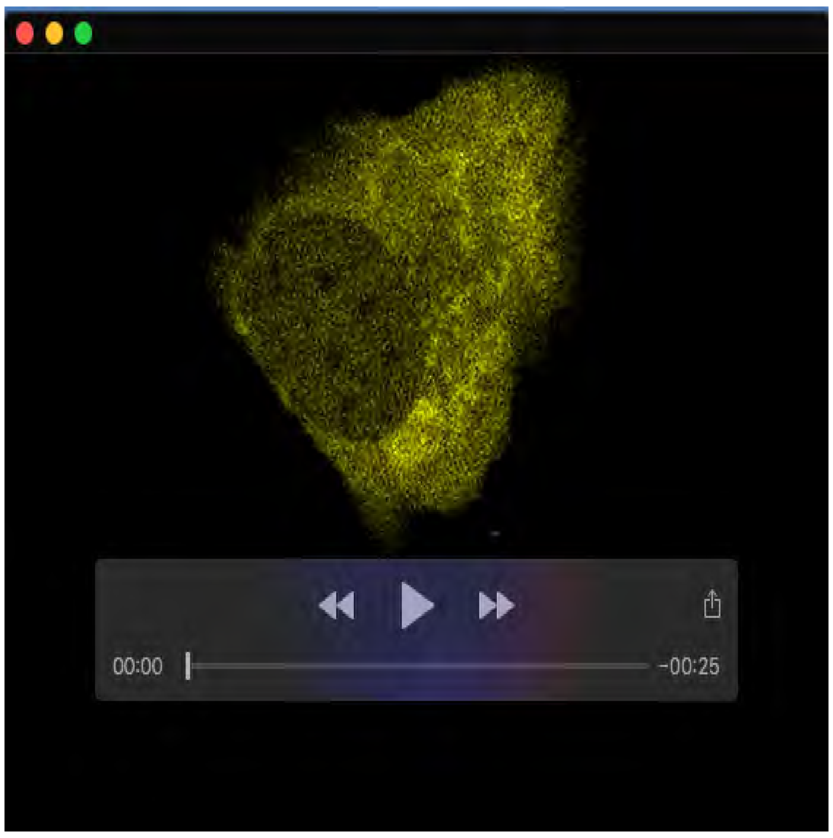


**Movie 1.** Movie shows a confocal section of a HaCaT cell expressing Zdk-mVenus release and recovery experiment. The total duration is 422 seconds.

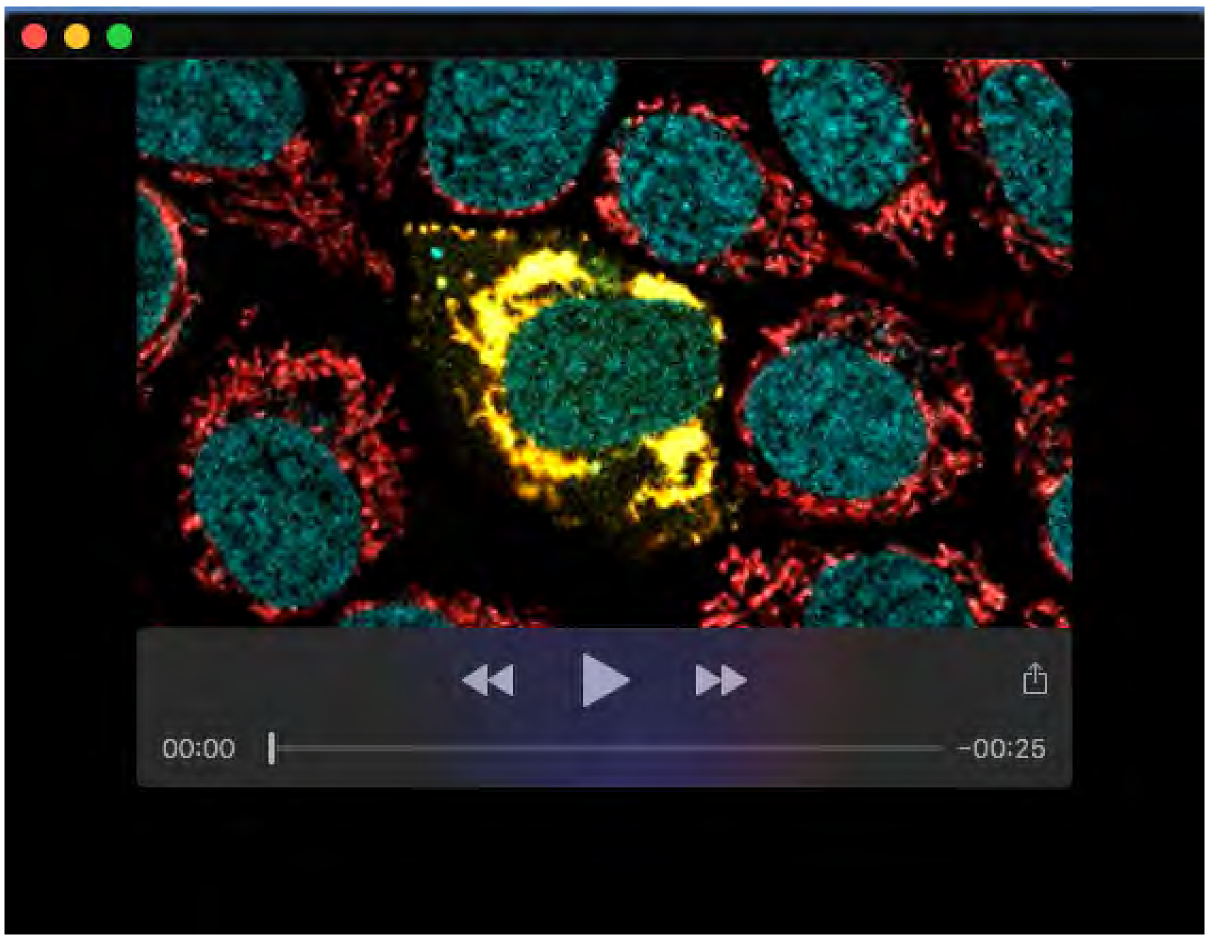


**Movie 2.** Movie shows a confocal section of a HaCaT cell expressing Zdk-mVenus-YAP1 release and recovery experiment. The total duration is 422 seconds.

Journal of Cell Science • Supplementary information

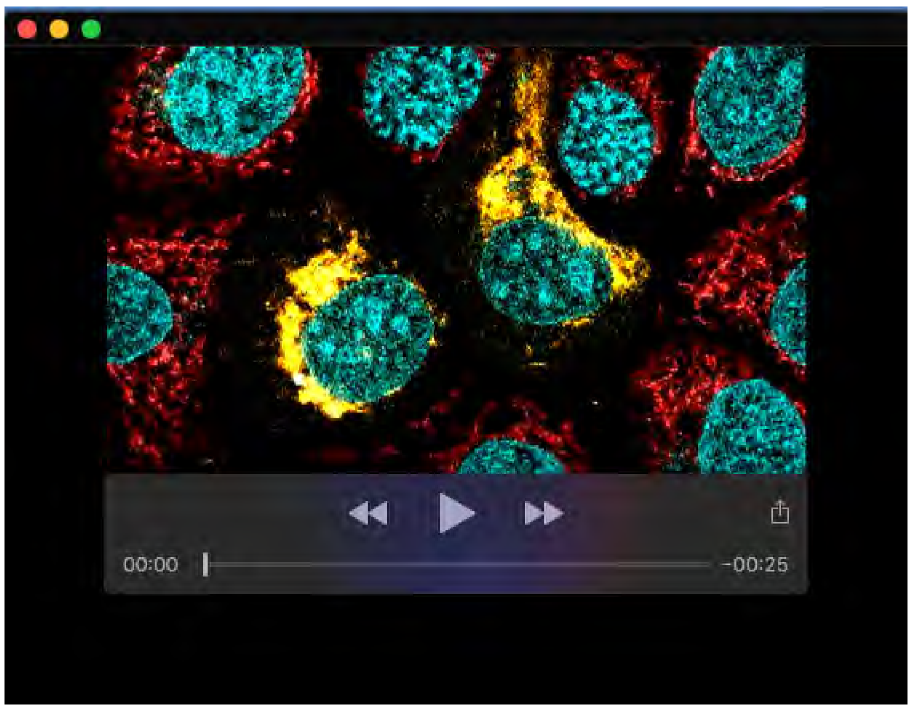


**Movie 3.** Movie shows a confocal section of a HaCaT cell expressing Zdk-mVenus-TAZ1 release and recovery experiment. The total duration is 422 seconds.

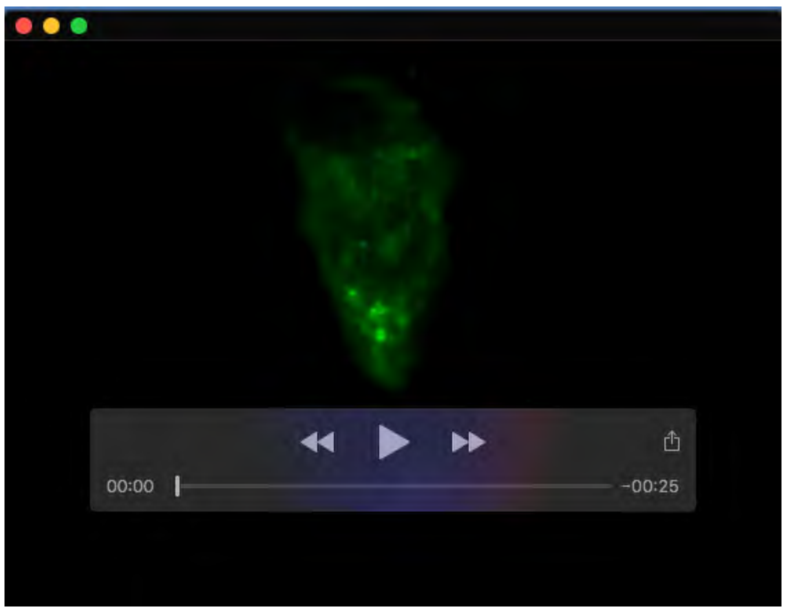


**Movie 4.** Movie shows 3D image of a HaCaT cell expressing Zdk-mCh-YAP1 (in green) release and recovery experiment, including three release 'bursts' during the recovery phase on the lattice lightsheet microscope. The total duration is 800 seconds.

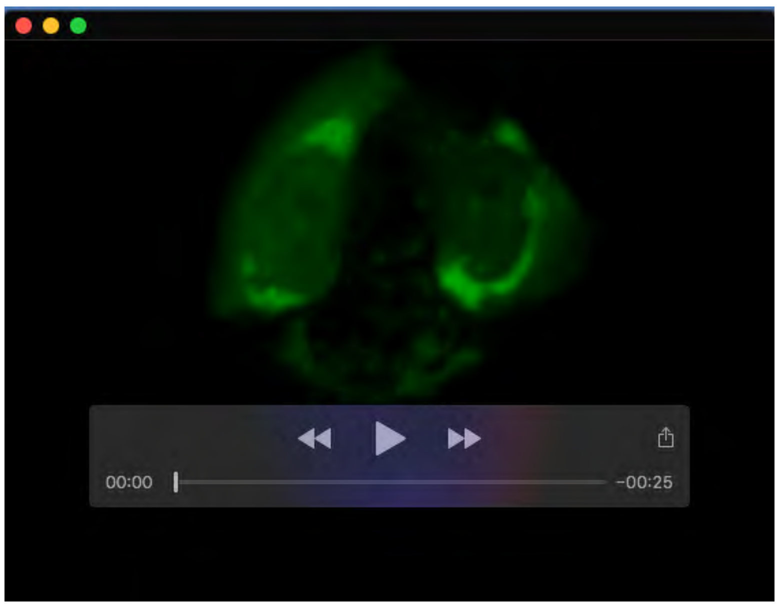


**Movie 5.** Movie shows 3D image of a HaCaT cell expressing Zdk-mCherry (in green) release and recovery experiment, including three release 'bursts' during the recovery phase on the lattice lightsheet microscope. The total duration is 800 seconds.

Journal of Cell Science • Supplementary information

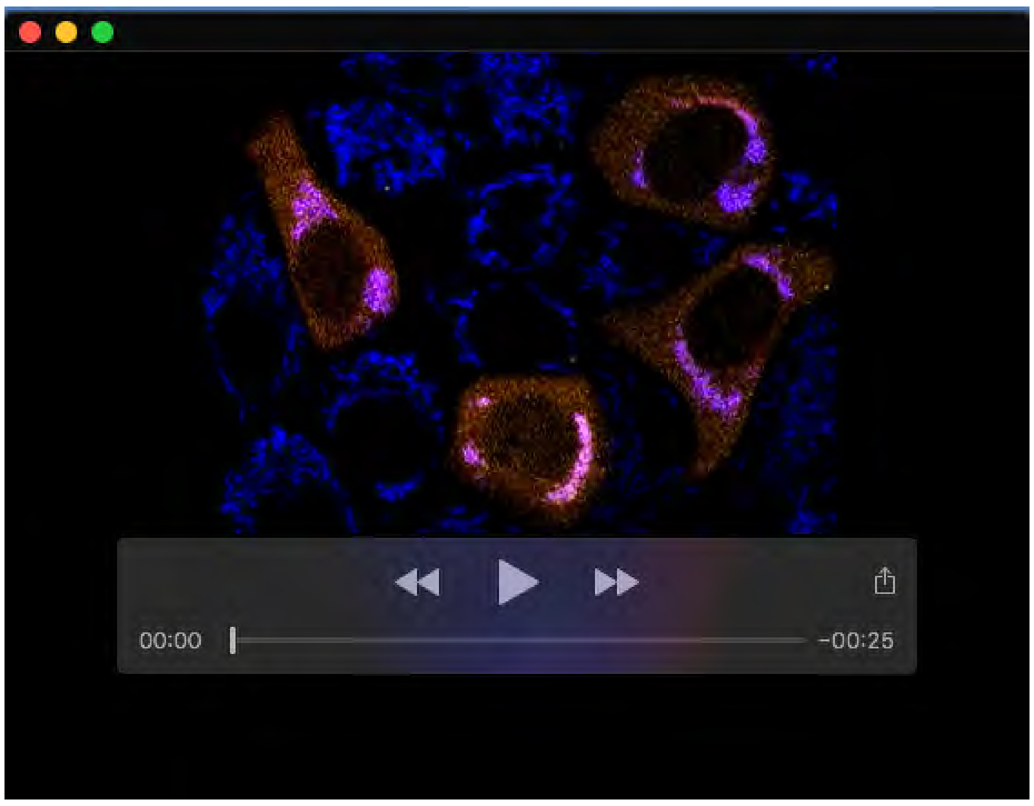


**Movie 6.** Movie shows a confocal section of a HaCaT cell expressing Zdk-mVenus-YAP1 and Zdk-mCh-TAZ release and recovery experiment. The total duration is 466 seconds.

**Table S1**

| Plasmid name | Colours | Promoter | Use | Source |
|---|---|---|---|---|
| pRK5.1-TOM20-flag-LOVwt | none | CMV | optogenetics | cloned |
| pRK5.1-Zdk-flag-mCh | mCherry | CMV | optogenetics | cloned |
| pRK5.1-Zdk-flag-mCh-YAP1_WT | mCherry | CMV | optogenetics | cloned |
| pRK5.1-Zdk-flag-mCh-YAP1_5SA | herry | CMV | optogenetics | cloned |
| pRK5.1-Zdk-flag-mCh-YAP1_S94A | mCherry | CMV | optogenetics | cloned |
| pRK5.1-Zdk-flag-mCh-TAZ | mCherry | CMV | optogenetics | cloned |
| pRK5.1-Zdk-flag-Venus | Venus | CMV | optogenetics | cloned |
| pRK5.1-Zdk-flag-Venus-YAP1_WT | Venus | CMV | optogenetics | cloned |
| pRK5.1-Zdk-flag-Venus-TAZ_WT | Venus | CMV | optogenetics | cloned |
| pRK5.1-Venus-YAP1_WT | Venus | CMV | control | cloned |
| pGL3-49 | luciferase | N/A | luciferase assay | Tapon lab |
| pGL3-5xMCAT-49 | luciferase | N/A | luciferase assay | Tapon lab |
| pcDNA3-Clover | Clover | CMV | filler plasmid for luciferase assay | Addgene #40259 |
| pGL4.75 CMV-Renilla | renilla | N/A | luciferase assay | Promega, #E6931 |
