# Supplementary material for: An optogenetic method for interrogating YAP1 and TAZ nuclear–cytoplasmic shuttling
Source: J Cell Sci. 2021 Jul 9;134(13):jcs253484. doi: 10.1242/jcs.253484 (PMC8313864; doi:10.1242/jcs.253484)
Supplement: Reviewer comments [file joces-134-253484_review_history.pdf]

An optogenetic method for interrogating YAP1 and TAZ nuclear-cytoplasmic shuttling

Anna M. Dowbaj, Robert P. Jenkins, Daniel Williamson, John M. Heddleston, Alessandro Ciccarelli, Todd Fallesen, Klaus M. Hahn, Reuben D. O'Dea, John R. King, Marco Montagner and Erik Sahai
DOI: 10.1242/jcs.253484

Editor: Andrew Ewald

Review timeline

Original submission:	24 August 2020
Editorial decision:	8 October 2020
First revision received:	19 April 2021
Editorial decision:	12 May 2021
Second revision received:	21 May 2021
Accepted:	21 May 2021

Original submission

First decision letter

MS ID#: JOCES/2020/253484

MS TITLE: An optogenetic method for interrogating YAP1 and TAZ nuclear-cytoplasmic shuttling.

AUTHORS: Anna M Dowbaj, Robert P Jenkins, John M Heddleston, Alessandro Ciccarelli, Todd Fallesen, Klaus Hahn, Marco Montagner, and Erik Sahai
ARTICLE TYPE: Tools and Resources

We have now reached a decision on the above manuscript.

To see the reviewers' reports and a copy of this decision letter, please go to: https://submit-jcs.biologists.org and click on the 'Manuscripts with Decisions' queue in the Author Area. (Corresponding author only has access to reviews.)

As you will see, the three reviewers are excited by the concept but raise a number of substantial criticisms about the validation of the new sensor, experimental design, and the presentation of both the text and figures that prevent me from accepting the paper at this stage. They suggest, however, that a revised version might prove acceptable, if you can address their concerns. If you think that you can deal satisfactorily with the criticisms on revision, I would be pleased to see a revised manuscript. We would then return it to the reviewers.

We are aware that you may currently be unable to access the lab to undertake experimental revisions. If it would be helpful, we encourage you to contact us to discuss your revision in greater detail. Please send us a point-by-point response indicating where you are able to address concerns raised (either experimentally or by changes to the text) and where you will not be able to do so within the normal timeframe of a revision. We will then provide further guidance. Please also note that we are happy to extend revision timeframes as necessary.

Please ensure that you clearly highlight all changes made in the revised manuscript. Please avoid using 'Tracked changes' in Word files as these are lost in PDF conversion.

© 2021. Published by The Company of Biologists under the terms of the Creative Commons Attribution License (https://creativecommons.org/licenses/by/4.0/).

I should be grateful if you would also provide a point-by-point response detailing how you have dealt with the points raised by the reviewers in the 'Response to Reviewers' box. Please attend to all of the reviewers' comments. If you do not agree with any of their criticisms or suggestions please explain clearly why this is so.

Reviewer 1

Advance summary and potential significance to field

The manuscript by Dowbaj et al. describes an optogenetic method to investigate nucleo-cytoplasmic shuttling of proteins, and apply it to study YAP/TAZ transcriptional regulators. The tool is based on LOV-TRAP, which is an optogenetic system for light-induced protein dissociation (Wang et al. 2016 Nature Methods). In the manuscript, the tool is utilized to recruit the proteins of interest to mitochondria, followed by light-induced release of the protein to the cytoplasm and then measurement of nucleo-cytoplasmic shuttling rates.
This manuscript is very interesting, and it has several potentially important points. The first is the use of optogenetics as a tool to measure the transport rate of proteins, which is a cool idea. Second, the ability to measure transport rates of two proteins simultaneously from the same cell is exciting, since this cannot be easily achieved with the current photobleaching-based methods. Third, recording the fluorescence fluctuations in the whole cell with the use of lattice-light sheet opens novel possibilities for studies on the intracellular heterogeneity.

However, there are several profound issues, starting with the functionality of the optogenetic tool and lack of controls, that undermine the impact of the manuscript in the present state.

Comments for the author

Major concerns Comparing the release rates of the constructs in Figures 1F (for mCherry) and 2D-E (for mCherry vs. YAT/TAZ) indicates that the release rate is not significantly different between dark and light conditions. This indicates that the tool is not functioning properly, e.g. the protein is not efficiently released upon the imaging conditions utilized here. Also the cell images seem to indicate the same; the increase in cytoplasmic intensity is very small. Why is there any release in dark conditions, and why would this depend on the utilized constructs (mCherry vs. YAP/TAZ in 1D)?

The experimental conditions, especially regarding the transient transfection of the constructs, are not fully explained, and there is no indication whether/how this was optimized. The ratio of the different components of the optogenetic system are bound to be critical for the functioning of the system and possible overexpression artifacts (and the presence of endogenous proteins in the system) should be controlled for, but these are not addressed or discussed. Different expression levels could be a source of the high cell to cell variability.

As mentioned above, the ability to measure the transport rates of two proteins simultaneously in the same cell is very exciting. However, it seems that this system gives different results compared to system measuring only one protein.

In figure 2G, TAZ is imported significantly faster than YAP, when measured separately. In figure 5B, YAP and TAZ import rates do not differ, when measured from the same cell.

Specific points In figure 1B, the cell images could be considerably bigger, since this is the proof-of-principle for the experiment, and it would be nice to easily see the effects. Also, the nuclear intensity appears very low.

It would be good to show the excitation/emission spectra for all the constructs, since the advantage of utilizing specifically these fluorophores is mentioned several times in the manuscript. Also, it is not clear from the materials and methods, how the excitations were done on the confocal, although this is very clearly explained for the lattice light sheet.

© 2021. Published by The Company of Biologists under the terms of the Creative Commons Attribution License (https://creativecommons.org/licenses/by/4.0/).

In 1C (and applies actually also to 2B,C), why does the fluorescence signal on mitochrondria rise above the initial values during the recovery phase? It might be useful to plot also the total cell intensity.

In figures 1F and G, it would be good to spell open the mCherry above the graphs.

Figure legend for 1F is weird.

Figure 3C lacks the r and p values for correlation.

Figure 4 contains a lot of data, and it is slightly difficult to identify the most meaningful data. I suggest to make a much more clearer distinction between those correlations that were statistically significant compared to those that were not. In addition, one parameter that should have been included here is intensity of the utilized construct in order to examine for the possible overexpression problems. It might be interesting to also think about other possible parameters, such as ratio of import and export or sum of import and export (overall shuttling speed) to draw out possible biologically meaningful correlations.

The use of lattice light sheet in Figure 6 is interesting, and especially the use of repeated pulses of release as “technical replicates” within the same cell, is exciting. However, the present analysis of the results do not really add too much to the story, and there is no attempt to study nuclear transport with this method. The images in figure 6B and C could be clearer, and the channels should be shown separately, because the mCherry signal masks the mito signal. How were the regions in 6E chosen? Figure 6F might be clearer, if shown only with the lines.

Discussion line 336: it is stated that the peptide does not interfere with the endogenous activity of YAP1 and TAZ with reference to figure S1E, which is actually a Western blot showing the expected sizes of the proteins. S1F is showing a luciferase assay, which demonstrate that the constructs can activate transcription, but with the presented data, it is too strong statement to say that the peptide does not interfere with endogenous activity.

Reviewer 2

Advance summary and potential significance to field

This manuscript describes the development of a new optogenetic tool for interrogating nuclear/cytoplasmic shuttling as well as a model and software package to analyze the associated data. These tools are then used to study the shuttling of YAP and TAZ. This is an exciting tool and should be used widely by the field. However, there are some missing controls and unjustified assumptions that prevent the manuscript from being suitable for publication at this time. Also, in places the manuscript is poorly written and there are many small errors in the text and figures.

Comments for the author

MAJOR CONCERNS

1. The optogenetic tool is based on LOV-TRAP system and is initially validated with mCherry. A natural control to verify proper functionality would have been to use mCherry with a nuclear localization and/or exportation sequences. Seeing the expected differences in the import and export rates of these constructs would further establish that the system is working as expected and show that accurate rates can be determined with the overall procedure. The authors should either add such controls or justify why they were not completed in the text.

2. Key controls / analyses are missing or at least nor clearly presented in the multicolor experiments. Do YAP-Venus and YAP-mCherry report the same import and export rates with imaged individually? If the YAP-Venus and YAP-mCherry constructs are imaged simultaneously, do they show the same import and export rates? Similarly, does TAZ-mCherry report the same import/export rates when imaged alone as well as imaged with YAP-Venus?
Without these simple controls the efficacy of this experimental set-up cannot be verified.

© 2021. Published by The Company of Biologists under the terms of the Creative Commons Attribution License (https://creativecommons.org/licenses/by/4.0/).

3. A major portion of the work is the development of a model to describe the observed data, however, the role of diffusion is ignored in this model. This omission is confusing as the authors have done this type of modelling before (Ege, Cell Sys, 2018). The justification for ignoring diffusion in this work should be established quantitatively and stated in the text. Additionally, the data acquired the lattice light sheet demonstrating variablility of import/export rates throughout the cell would be most easily explained by local differences in YAP diffusivity.

4. The data obtained with the lattice light sheet seem very preliminary, as the number of measurements seems quite low. Also, it is challenging to interpret these results as presented with a model that does not contain diffusion. Additionally, experiments with mCherry should be included to establish that the observed spatial variation is related to YAP functionality and not physical process, such as molecular crowding. The authors should either substantially increase the quality of this data or consider removing it. Its inclusion does not bolster the main points of the paper, so this reviewer recommends removing it.

5. The “Differential equation to model nuclear import and export” section should be re-written, or the title of this section should be changed to reflect the fact that there is substantially more in this section rather than just the equations. This reviewer suggests the comparison to FLIP be given its own section, as this is key to the validation of the approach.

6. The authors should discuss how the estimates of YAP1 import/export and TAZ import/export compare with previous measurements in the “Application of opto-release methodology to YAP and TAZ” section. The consistency with FLIP demonstrates internal consistency of the study, but consistency with previous measurements should also be established.

7. As transient transfections were used to create the system, large variations in expression levels between cells in the population are likely.
The authors should show data demonstrating that the results are not dependent on the absolute expression levels of the transfected components.

8. The maximal nuclear accumulation of YAP using this system (for example in Fig 2), is quite low throughout the experiments in the manuscript. It can’t be determined if this was due to low levels of YAP nuclear localization or incomplete release of YAP from the mitochondria due to a defect in the optogenetic tool. The authors should perform experiments distinguishing between these two possibilities.

9. The text in the “Import and Export Rates” section is vague and unorganized. It should be rewritten to provide more precise explanation and interpretation of the data. Also, the title needs adjusting. I believe the main point is the import and export rates are correlated for YAP but not other constructs.

10. The development of the semi-automated software is presented as a significant part of the work. Including a supplemental figure that demonstrates the proper functionality of the software on simulated data would provide definitive proof that the code is functioning properly.

11. Figure captions generally lack key details, like the number of cells in each experiment and number of experimental days. More detail should be added to these captions.

12. The availability of the MATLAB code is not stated.

MINOR COMMENTS
1. The time courses in all figures should be converted to time from frame number.
2. Is the mCherry data repeated from Figure #1 to Figure #2? If so this should be stated. Also, how does this effect the statistical comparisons?
3. In Figure #3C is the distribution of YAP_S94A bimodal?
4. In Figure #5, the positions of the various ROI should be shown. Are the 5 regions equidistant from the nucleus?
5. Figure #1 shows YAP1/TAZ in the schematic but all of the data regard mCherry.
6. What is shown in Sup Fig #8 and its relevance to the manuscript is unclear.

© 2021. Published by The Company of Biologists under the terms of the Creative Commons Attribution License (https://creativecommons.org/licenses/by/4.0/).

7. Image size/quality is generally-low throughout the manuscript and ideally larger-size images (in both manuscript footprint and quality) should be included.
8. Page 11, line 276: It might be noted here that YAP does not have a canonical NLS “Importin alpha1 mediates Yorkie nuclear import via an N-terminal Non-canonical nuclear localization signal. J Biol Chem 291, 7926–7937”

Reviewer 3

Advance summary and potential significance to field

see below

Comments for the author

The paper “An optogenetic method for interrogating-…” authored by Dowbaj and colleagues reports on the development and use of an AsLOV-based optogenetic tool to control the cellular localization (including mitochondria, cytoplasm and nucleus) of the YAP transcription factor. Using this approach, the authors quantify, using a MATLAB-based app, the rates of nuclear entry/exit under a variety of conditions. Finally, they combine the optogenetic tool with the use of lighsheet microscopy to measure the dynamics of the transcription factor within the cell in 3D. I think the paper has very interesting and novel elements (such as the use of a YAP optogenetic tool and the capability of its 3D tracking), however the quantification part suffers from multiple fundamental mathematical flaws that unfortunately massively impact the quality of the work. I honestly hope that the points below help the authors with re-analysis.

Major points
1. The model has 2 different rate constants for protein unbinding from the mitochondria, depending on whether blue light is on or off i.e. whether the LOV domain is excited or relaxed. However, the rate constant for protein binding to the mitochondria must also take on 2 different values depending on the LOV domain conformation. Without taking this into account, the model is unphysical and, due to all obtained rate constants being interdependent (since they are fit simultaneously), all rate constants obtained with the model in its current form suffer from this.

2. On lines 756-758, the authors state that relaxation of AsLOV2 domains occurs on the timescale of seconds. Therefore, for illumination to be considered constant, light should be supplied at a rate at least an order of magnitude higher than the relaxation rate, ideally continuously (simply achieved using widefield illumination). If light is supplied to LOV domains at a rate equal to their relaxation rate, between illumination pulses the fraction of stimulated domains will drop exponentially to 0.37=exp(-1)). This is particularly important given the interconnected nature of the measured rate constants.

3. A particularly worrying point is that, after analysis (in Fig. 1F for mCherry and other figures for other constructs) there is no significant difference in the rate constant of mitochondrial release when the light is on (“mito light”) or off (“mito dark”). The former should be many times greater than the latter – as they are, these values would state that stimulation does not work.

4. The rate constants (k’s) found here have inherent dependence on cellular parameters such as cytoplasmic/nuclear volume. This would be clear if the differential equations were derived from first principles. E.g. Timney et al. JCB 215, 57 (2016) use differential equations derived from first principles, so measured rate constants can be transformed into quantities independent of nuclear/cytoplasmic volumes and number of NPCs etc. Only after a transformation such as this can correlations be investigated. A similar transformation needs to be applied to rate constants measured in this study before any correlations between rate constants and cellular parameters, or between import and export rate constants, can be performed fairly.

© 2021. Published by The Company of Biologists under the terms of the Creative Commons Attribution License (https://creativecommons.org/licenses/by/4.0/).

Other major points
Major.
5. The methods of “bleaching intensity normalisation” and “non-conserved intensity correction” are overcomplicated and introduce a troubling number of free parameters into the data processing. If simply c(t)/m(t)/n(t) are the cytoplasmic/mitochondrial/nuclear intensities, normalised by their combined intensity (i.e. whole cell intensity) then photobleaching will automatically be accounted for and c(t)+m(t)+n(t)=1 at all times, thus an outflow-inflow function is not required.

6. Fig. 1B supposedly shows H2B-mTurquoise labelling the nucleus. Can the authors also show this channel individually (not merged), as it has a sparse, speckled appearance? In addition, with reference to lines 760-762, it is hard to see how imaging of H2B-mTurquoise does not interfere with optogenetic activation despite using the same wavelength. Fluorescent imaging typically needs much higher intensity than optogenetic stimulation.

7. There are 2 examples of data being processed inequivalently: lines 160-162, constant thresholding is applied to some cells, dynamic thresholding to others; lines 1016-1020, an inflow- outflow function is applied to some cells, and not others. Making any comparison or assimilation of data that has not been processed in exactly the same manner is difficult.

8. Almost every instance of “rate” throughout the paper, in the text and figures, should be “rate constant” – the model used finds rate constants. This distinction is very important and conceptually crucial.

9. Throughout this work, data has been normalised, but it is never made clear with respect to what. For example, in Fig. 1C, and similar graphs, the vertical axis is called intensity, but it is clearly normalised (I assume to the intensity of the entire cell, and these numbers represent the fraction of intensity that comes from the mitochondria, cytoplasm, and nucleus, but this is not made clear). Since these values are input into quantitative modelling, it is vital that they are clearly explained. In particular, I wonder whether the data has been normalised by the the cellular area (in confocal miscoscopy) or the cellular volume (in lightsheet microscopy).

10. Several statements are not supported by the evidence provided: lines 111-113, neither figure shows information on expected localisation; line 120-122, Fig. S1C does not show enrichment to mitochondria; lines 125-127, Fig. 1B does not show an increase in cytoplasmic fluorescence; lines 228-231, YAP1_5SA has a low import rate as well as a low export rate, so the claim that nuclear persistence is a result of a low export is not justified; lines 247-250, there is no data/figure to support this claim.

First revision

Author response to reviewers' comments

Reviewer 1

The manuscript by Dowbaj et al. describes an optogenetic method to investigate nucleo-cytoplasmic shuttling of proteins, and apply it to study YAP/TAZ transcriptional regulators. The tool is based on LOV-TRAP, which is an optogenetic system for light-induced protein dissociation (Wang et al. 2016, Nature Methods). In the manuscript, the tool is utilized to recruit the proteins of interest to mitochondria, followed by light-induced release of the protein to the cytoplasm and then measurement of nucleo-cytoplasmic shuttling rates. This manuscript is very interesting, and it has several potentially important points. The first is the use of optogenetics as a tool to measure the transport rate of proteins, which is a cool idea. Second, the ability to measure transport rates of two proteins simultaneously from the same cell is exciting, since this cannot be easily achieved with the current photobleaching-based methods. Third, recording the fluorescence

© 2021. Published by The Company of Biologists under the terms of the Creative Commons Attribution License (https://creativecommons.org/licenses/by/4.0/).

fluctuations in the whole cell with the use of lattice-light sheet opens novel possibilities for studies on the intracellular heterogeneity.

However, there are several profound issues, starting with the functionality of the optogenetic tool and lack of controls, that undermine the impact of the manuscript in the present state.

We are pleased that the reviewer finds that our approach is a ‘cool idea’ and the ability to measure the dynamics of two proteins simultaneously ‘exciting’. Nonetheless, we also note his/her concerns and thank him/her for the constructive critique.

Major concerns

Comparing the release rates of the constructs in Figures 1F (for mCherry) and 2D-E (for mCherry vs. YAT/TAZ) indicates that the release rate is not significantly different between dark and light conditions. This indicates that the tool is not functioning properly, e.g. the protein is not efficiently released upon the imaging conditions utilized here. Also the cell images seem to indicate the same; the increase in cytoplasmic intensity is very small. Why is there any release in dark conditions, and why would this depend on the utilized constructs (mCherry vs. YAP/TAZ in 1D)?

The reviewer points out the information originally presented was insufficient to be confident that our tool was working in the intended way. Specifically, he/she queries the small degree of release of the Zdk-tagged protein from the mitochondrial LOV anchor. We agree that the data provided in the original submission was not optimal and have now taken several steps to address this.

1. We have implemented a new orthogonal method to analyse the on and off rate constants of Zdk and LOV interaction. In collaboration with the School of Mathematics at the University of Nottingham, we recently developed an analytical method for simultaneous deriving diffusion and on and off rate constants from confocal FRAP data. The theoretical part of this work is now accepted for publication following peer review in the Journal of Mathematical Biology. Using this approach, we have measured the on and off rate constants for Zdk LOV interactions in both the dark and the light, and Zdk on and off rate constants in the absence of any LOV protein (in this context it would not be expected to have any high affinity binding partners). In the absence of any LOV domain protein, the inferred on and off rate constants are 500 times higher indicating no long-lived interaction with any immobile proteins (Figure 1G). Crucially, there is an increase in the off rate constant in blue light with no change in Zdk- mCherry diffusion (Figure 1H & Figure 1J). These data clearly support that our construct is working. The reviewer may additionally query why the magnitude of the change in off rate constant under blue light that we observe is only 2-4 fold, and not greater. The reason for this is that we deliberately chose a LOV / Zdk combination with a lower dynamic range - the original analysis of the different LOV & Zdk mutants and differences depending on which component (LOV or Zdk) is tethered to mitochondria are reported in Wang et al., Nature Methods 2016. In contexts where one wishes to control a biochemical function with light, a large dynamic range between dark and light is desirable. However, our goal is to release only a small amount of protein so that we don’t overwhelm the normal regulatory mechanisms controlling nuclear import or export, hence our choice of a LOV /Zdk pairing with a lower dynamic range.

2. In addition to the new orthogonal FRAP data, we have also re-analysed and re-plotted the original data (shown in the figure below for the reviewer’s benefit). Although there was no significant difference between the means of the off rate constant in dark and light conditions in the original submission, when the data is plotted as paired measurements, which is entirely appropriate as the off rate constant is measured in the same cell in the dark and light, then light always leads to an increase in off rate constant (top left panels below). Following a very insightful suggestion from reviewer#3, we now allow different on rates in the dark and light. Thus, in the new analysis the magnitude of the increase in off rate constant in the light is increased. It also leads to tighter clustering of the rate constants in the dark. In all cases, blue light shifts the ratio of off and on rate constants in favour of the off rate constant (shown on the right below)

© 2021. Published by The Company of Biologists under the terms of the Creative Commons Attribution License (https://creativecommons.org/licenses/by/4.0/).

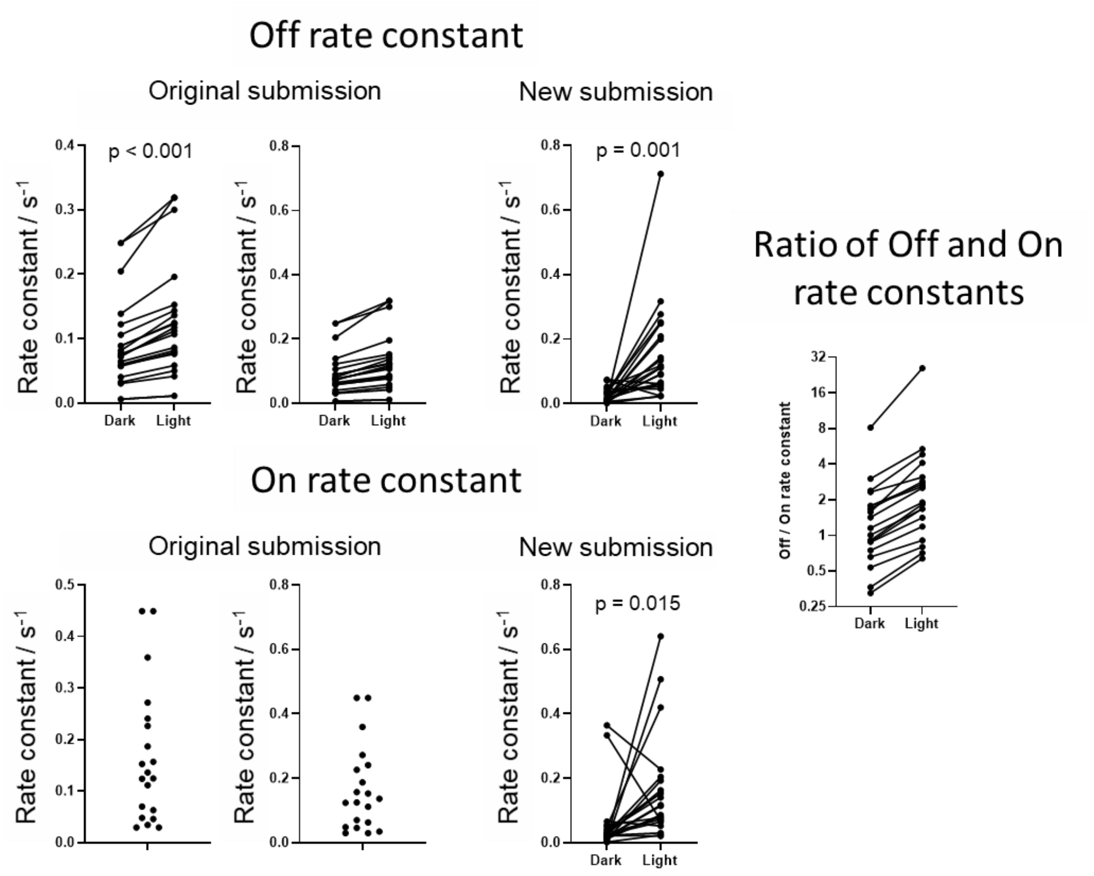

Reviewer Figure 1: Comparison of original mitochondrial binding and unbinding rate constants with new analysis. Upper panels show Off rate constants and lower panels show On rate constants. Left plot shows the data from the original submission, with lines linking the Dark and Light values for each cell. Middle plots show the same data, but with a scale equivalent to that in the new submission. Off and On rate constant plots are reproduced from the new submission (Figure 2). Right hand plot shows the change in ratio between Off and On rate constants for cells in the Dark and Light. Wilcoxon paired non- parametric statistical testing is reported.

The reviewer may also consider the absolute values determined by the two methods. These are reassuringly similar; with FRAP methodology reporting the off-rate constants to be 0.01-0.02s^{-1} in the dark and 0.03-0.075s^{-1} in the light and the opto-release methodology reporting 0.005-0.07s^{-1} in the dark and 0.024-0.9s^{-1} in the light. The main difference is the wider range of values reported using the opto-release methodology. Overall, it is striking how concordant the results are given the differences in methods.

3. We have replaced the images with clearer higher magnification images in Figures 1, 2, and 5.

Regarding the more general question about release in the dark, even high affinity interactions between two proteins are associated with on and off rate constants. It is not the case that, once bound, two proteins that exhibit a high affinity interaction remain bound forever. Hence, there are measureable on and off rates even in the dark. In the new fitting of the data there are no significant differences in the off or on rate constants for mCherry, YAP1, and TAZ (either in the dark or the light-Kruskal-Wallis multiple testing). The increase in off rate constant in the light is consistently highly significant, whereas the increase in the on rate constant is of varying significance. Once again, this confirms that the major effect of blue light illumination is to increase the off rate constant.

The experimental conditions, especially regarding the transient transfection of the constructs, are not fully explained, and there is no indication, whether/how this was optimized. The ratio of the different components of the optogenetic system are bound to be critical for the functioning of the system, and possible overexpression artifacts (and the presence of endogenous proteins in the

© 2021. Published by The Company of Biologists under the terms of the Creative Commons Attribution License (https://creativecommons.org/licenses/by/4.0/).

system) should be controlled for, but these are not addressed or discussed. Different expression levels could be a source of the high cell to cell variability.

The reviewer is correct that the relative ratio of constructs is an important factor. To achieve the maximum number of cells transfected with both constructs we typically transfected 1:1 or 1:2 ratios of TOM-LOV and Zdk-fusion constructs. Additionally, we now explain that cells were selected for low to moderate levels of fluorescent protein expression and effective sequestration on mitochondria in the dark (Methods lines 757 - 758). We have now performed new immuno-fluorescence analysis to address the relative stochiometry of the TOM-LOV and Zdk-fusion proteins in cells that met the criteria for analysis. Briefly, we stained in parallel wells transfected with either Zdk-Flag-mCherry-YAP1 and untagged TOM20-LOV or Zdk-mCherry-YAP1 and Flag-tagged TOM20-LOV. Anti-Flag immuno-staining was then performed. This was followed by identification of cells with levels and sequestration of mCherry consistent with their selection for optogenetic analysis. These cells were then imaged for the intensity of anti-Flag signal, which enabled comparison of the levels of expression of the TOM20-LOV construct and Zdk-mCherry fusion in cells that met the selection criteria for optogenetic analysis. This analysis revealed a 2x fold excess of the TOM20-LOV construct – now shown in Supp. Fig. 1D.

In addition to the analysis above of relative LOV and Zdk expression levels, we also compared the level of Zdk-FP-YAP1 over-expression in cells meeting the criteria for opto-genetic analysis with endogenous YAP1 levels in neighbouring un-transfected cells. These data are now shown in Supp. Fig. 3B and reveal a 2-4 fold level of over-expression. When this is considered with the additional information that roughly 10-15% of the sequestered protein is released by light, we can deduce that our opto-genetic methodology releases an amount of protein equivalent to 20-50% of the endogenous. This level is well-suited for imaging and, crucially, is unlikely to overwhelm the regulatory mechanisms that govern endogenous YAP1 or TAZ localisation.

The reviewer rightly asks whether expression level of the construct bears any relationship to the values that are measured. Supplementary Figure 2B now shows there is no relationship between the mean fluorescent intensity and the import or export rate constants. Similar results were obtained if plots were generated considering the total fluorescent intensity, but these are not shown due to space constraints.

As mentioned above, the ability to measure the transport rates of two proteins simultaneously in the same cell is very exciting. However, it seems that this system gives different results compared to system measuring only one protein. In figure 2G, TAZ is imported significantly faster than YAP, when measured separately. In figure 5B, YAP and TAZ import rates do not differ, when measured from the same cell.

The reviewer raises an important issue about different methodologies yielding the same results. In the original submission, although the rates measured for YAP and TAZ import and export were broadly concordant between the single opto-release, double opto-release, and cyto-FLIP methods there were some small discrepancies (e.g. the difference in YAP vs TAZ import was significant in the single, but not double or cyto-FLIP measurements). As outlined above, we have now re-fitted all the original data with the modelling using starting mitochondrial off and on rate constants determined by FRAP and a variable on rate constant in the lit condition. This has reduced the spread of the data, with some of the outlying values for TAZ being reduced. The result is that the originally reported difference between YAP and TAZ is less and not statistically significant. We had not placed much emphasis on this result in the original submission, using the phrase ‘slightly faster’ to describe the difference between TAZ and YAP import and export rates. We have now removed that claim.

More generally, the reviewer is quite right that the methods should yield the same data. Therefore, we now present overlaid plots of the single, double, and cyto-FLIP import and export measurements for YAP and TAZ (single and cyto-FLIP plot is shown in Supp. Fig. 3H and single and double plot is shown in Figure 6D). For the benefit of the reviewer, all three are overlaid below and reveal good concordance between the different methods.

© 2021. Published by The Company of Biologists under the terms of the Creative Commons Attribution License (https://creativecommons.org/licenses/by/4.0/).

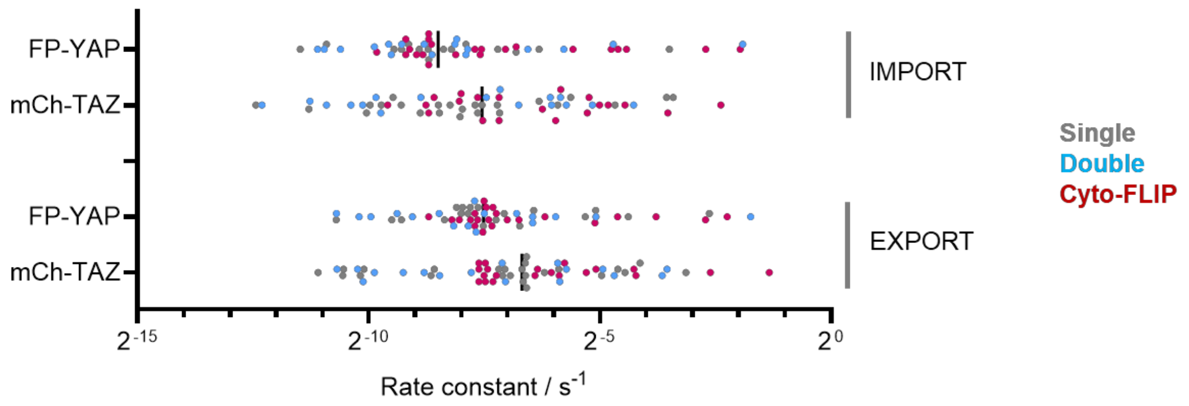

Reviewer Figure 2: Comparison of the import and export rate constants derived using single channel opto-release (grey), double channel opto-release (light blue), and cytoplasmic FLIP (red).

Specific points

In figure 1B, the cell images could be considerably bigger, since this is the proof-of-principle for the experiment, and it would be nice to easily see the effects. Also, the nuclear intensity appears very low.

The reviewer makes a good point, we have replaced the images throughout the manuscript with higher magnification examples. The movies have also been improved. Figure 1B&C now concentrate on mCherry and demonstrating that the system is functioning with quantification of the changes in shown in Figure 1C provided in Figure 1D. Figure 3 now contains the data for YAP1. The relatively low nuclear signal is a reflection that for confluent HaCAT cells, the equilibrium position of both endogenous and exogenous YAP1 is in the cytoplasm and that the Zdk-FP-YAP1 is released into the cytoplasm.

It would be good to show the excitation/emission spectra for all the constructs, since the advantage of utilizing specifically these fluorophores is mentioned several times in the manuscript. Also, it is not clear from the materials and methods, how the excitations were done on the confocal, although this is very clearly explained for the lattice light sheet.

We agree that the excitation and emission information should be provided for the confocal imaging and now do so in the Methods (lines 769-780). We also explain that if the goal is to only image one protein, then Venus excited at 514nm, Mitotracker Red excited at 561nm, and DRAQ5 excited at 633nm is a good combination. If the ultimate goal is to image two proteins simultaneously, then Venus and mCherry are a good pairing.

In 1C (and applies actually also to 2B,C), why does the fluorescence signal on mitochrondria rise above the initial values during the recovery phase? It might be useful to plot also the total cell intensity.

The original plots showed the average of the all the cells and the elevation of the recovery signal above the starting phase was caused by a couple of cells with outlier signals following normalization. In the re-submission we show exemplars for single cells and the improved fitting and normalization methodology described in reviewer #3 point #5 means that these slightly erroneous fits are no longer generated.

In figures 1F and G, it would be good to spell open the mCherry above the graphs.

We have now made sure that we use consistent and clear labelling across all figures. Figure 1 now shows Zdk-Venus images. For space reasons, we still use abbreviated forms of fluorophore names

© 2021. Published by The Company of Biologists under the terms of the Creative Commons Attribution License (https://creativecommons.org/licenses/by/4.0/).

in some places with 'Ven' used to connote Venus and 'mCh' used to connote mCherry.

Figure legend for 1F is weird.

We apologise for this and have re-written all the figure legends. Figure 3C lacks the r and p values for correlation.
We have now added these to Figures 4B (which was previously 3C) and 6E.

Figure 4 contains a lot of data, and it is slightly difficult to identify the most meaningful data. I suggest to make a much more clearer distinction between those correlations that were statistically significant compared to those that were not. In addition, one parameter that should have been included here is intensity of the utilized construct in order to examine for the possible overexpression problems. It might be interesting to also think about other possible parameters, such as ratio of import and export or sum of import and export (overall shuttling speed) to draw out possible biologically meaningful correlations.

We thank the reviewer for these good suggestions. We now provide a plot showing the relationship between intensity and various metrics in Supplementary Figure 2B. The plots showing the correlations have been simplified to focus on possible associations between import and export and morphological features of the cells. This means that trivial correlations, such as between cell area and perimeter, are no longer included. Regarding the statistical significance, we have modified the presentation to make the scaling of the circles vary more dramatically as a function of statistical significance. We are reluctant to make simple binary distinction between significant and non-significant because the choice of cut-off is always somewhat arbitrary. By including the graphical representation of the p values, the reader has the best overview of the relative strengths of different correlations. Finally, we have streamlined the whole section to focus on two observations, the correlation between import and export and the correlation between the import/export ratio and nuclear/cytoplasmic area.

The use of lattice light sheet in Figure 6 is interesting, and especially the use of repeated pulses of release as "technical replicates" within the same cell, is exciting. However, the present analysis of the results do not really add too much to the story, and there is no attempt to study nuclear transport with this method. The images in figure 6B and C could be clearer, and the channels should be shown separately, because the mCherry signal masks the mito signal. How were the regions in 6E chosen? Figure 6F might be clearer, if shown only with the lines.

The reviewer requests greater depth and improved presentation of the light-sheet data. We now present both the merged image showing Zdk-mCh-YAP1, mitochondria, and the nucleus in Figure 5B and only the Zdk-mCh-YAP1 in Figure 5C. We provide a supplementary panel (Supp. Fig. 5B) showing the different cytoplasmic regions that are analysed in Figure 5E. To integrate the light-sheet data more thoroughly, we now additionally use confocal methods to explore the variation in cytoplasmic dynamics in more detail using confocal methods. Finally, the reviewer comments that we do not study the nuclear transport using the light-sheet method. The original purpose of the light-sheet analysis was to study the distribution of the Zdk-fusion construct throughout the whole cell. Our analysis of the data revealed greater variation in the Zdk-fusion protein in the cytoplasm, hence we focused on this.

We have also moved the light-sheet data to be both the 'double opto-release' experiments. This makes in more integrated with the rest of the manuscript.

Discussion line 336: it is stated that the peptide does not interfere with the endogenous activity of YAP1 and TAZ with reference to figure S1E, which is actually a Western blot showing the expected sizes of the proteins. S1F is showing a luciferase assay, which demonstrate that the constructs can activate transcription, but with the presented data, it is too strong statement to say that the peptide does not interfere with endogenous activity.

The reviewer is correct that this is an over-statement based on the data presented. We have modified this to state that the Zdk-mCherry-YAP1 was able to efficiently activate transcription from a TEAD reporter (lines 231-236, data are now in Supp. Fig. 3D). Furthermore, similar to the endogenous YAP1, the Zdk-FP-YAP1 fusion is dependent on TEAD binding and is inhibited by

© 2021. Published by The Company of Biologists under the terms of the Creative Commons Attribution License (https://creativecommons.org/licenses/by/4.0/).

phosphorylation (analysis of mutants in Supp. Fig. 3D).

Reviewer 2

Advance Summary and Potential Significance to Field: This manuscript describes the development of a new optogenetic tool for interrogating nuclear/cytoplasmic shuttling as well as a model and software package to analyze the associated data. These tools are then used to study the shuttling of YAP and TAZ. This is an exciting tool and should be used widely by the field. However, there are some missing controls and unjustified assumptions that prevent the manuscript from being suitable for publication at this time. Also, in places the manuscript is poorly written and there are many small errors in the text and figures.

We are delighted that the reviewer comments that 'this is an exciting tool and should be used widely by the field'. We also note a wide range of thoughtful suggestions to improve the work.

MAJOR CONCERNS

1. The optogenetic tool is based on LOV-TRAP system and is initially validated with mCherry. A natural control to verify proper functionality would have been to use mCherry with a nuclear localization and/or exportation sequences. Seeing the expected differences in the import and export rates of these constructs would further establish that the system is working as expected and show that accurate rates can be determined with the overall procedure. The authors should either add such controls or justify why they were not completed in the text.

The reviewer proposes a logical test for the verification of the system. While we agree that this should show a difference in import and export rates between mCherry with or without an NLS, it would not confirm that the values derived were quantitatively correct. We consider this latter point a priority and therefore pursued verification via the route of comparing our optogenetic method with more conventional FLIP measurements. In the revised version of the manuscript, we have now extended this approach to independently measure the changes in mitochondrial binding rates in the dark and light (overlay plot is shown in Supp. Fig. 3H). The high level of concordance between independent FRAP measurements, in some cases performed by different individuals on different microscopes, provides confidence that our system is providing reliable data.

In addition, we have added new data in Supplementary Figure 3E&F showing the exit of the Zdk-FP- YAP1 from the nucleus is slower in the presence of leptomycin B, which blocks Crm1-mediated nuclear export of protein containing either canonical or non-canonical NES sequences. This observation confirms that the method can report on well-established mechanisms of nuclear export and is consistent with previous reports indicating that YAP1 nuclear export depends on Crm-1 acting on a non-canonical export signal (Ege, Dowbaj, et al., Cell Systems 2018 and references therein).

COVID note – In normal circumstances, we would have sought to generate and test an NLS version of the mCherry construct and directly address the reviewer's comment. However, since receiving the reviewers' comments in October London has had very high COVID rates and three lockdowns of varying severity. This has meant that the lab has only been allowed to function at 25-30% of normal capacity until March and we have had to prioritise experiments very carefully. Following interaction with the editor, we prioritized the experiments showing that the system was responding appropriately to light in terms of mitochondrial binding, diffusion, heterogeneity in cytoplasmic YAP1 dynamics, and the relative levels of LOV, Zdk-YAP1, and endogenous YAP1.

2. Key controls / analyses are missing or at least nor clearly presented in the multicolor experiments. Do YAP-Venus and YAP-mCherry report the same import and export rates with imaged individually? If the YAP-Venus and YAP-mCherry constructs are imaged simultaneously, do they show the same import and export rates? Similarly, does TAZ-mCherry report the same import/export rates when imaged alone as well as imaged with YAP-Venus? Without these simple controls the efficacy of this experimental set-up cannot be verified.

© 2021. Published by The Company of Biologists under the terms of the Creative Commons Attribution License (https://creativecommons.org/licenses/by/4.0/).

The reviewer makes a good point. We now present data in Supplementary Figure 6D that the same rates are measured in both the single and double experiments. For the benefit of the reviewer we present an overlay of single and double opto-release measurements with cytoplasmic FLIP.

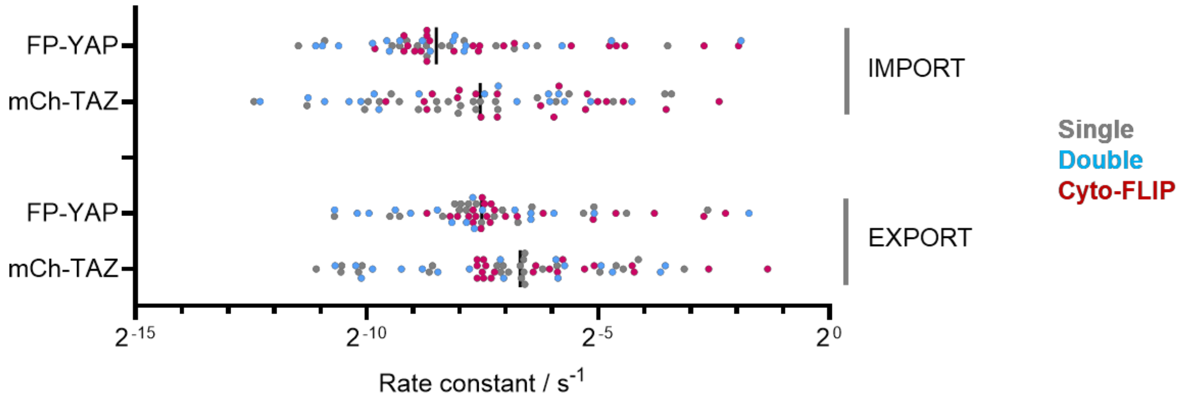

Reviewer Figure 2: Comparison of the import and export rate constants derived using single channel opto-release (grey), double channel opto-release (light blue), and cytoplasmic FLIP (red).

3. A major portion of the work is the development of a model to describe the observed data, however, the role of diffusion is ignored in this model. This omission is confusing as the authors have done this type of modelling before (Ege, Cell Sys, 2018). The justification for ignoring diffusion in this work should be established quantitatively and stated in the text. Additionally, the data acquired the lattice light sheet demonstrating variablility of import/export rates throughout the cell would be most easily explained by local differences in YAP diffusivity.

The reviewer is correct that diffusion could be an important factor. To address this we have implemented a method that directly measures diffusion and the on and off rates of binding to an immobile partner. The measured diffusion rates are 20-40μm^{2}s^{-1} (Figure 1F, 1J, & Supp. Fig. 5F)/ These rates are sufficiently fast relative to nuclear import and export that it will have only minimal impact of the rate constants that we measure. A mathematical justification of this is now provided in the supplementary information. Also pertinent to this point, we see limited variation in diffusion across different regions of cells and between different cells. In contrast, the measured rates of YAP1 binding to an immobile partner show much greater variability. These new data generated using a novel analysis method applied to conventional FRAP data are presented in Figure 1 with the mathematical basis of the analysis explained in a paper accepted at the Journal of Mathematical Biology. Together, these data provide support to the light-sheet data. Moreover, they suggest that the regional differences in YAP1 behaviour represent the variable distribution in an unknown binding partner, not diffusion.

4. The data obtained with the lattice light sheet seem very preliminary, as the number of measurements seems quite low. Also, it is challenging to interpret these results as presented with a model that does not contain diffusion. Additionally, experiments with mCherry should be included to establish that the observed spatial variation is related to YAP functionality and not physical process, such as molecular crowding. The authors should either substantially increase the quality of this data or consider removing it. Its inclusion does not bolster the main points of the paper, so this reviewer recommends removing it.

The reviewer raises valid points regarding the light-sheet data. We have now performed additional experiments using confocal methodology, both FRAP and optogenetic, to study the cytoplasmic dynamics of YAP1. As now presented in Supp. Fig. 5F, we have now measured diffusion of YAP1 and find that it shows low variation. In contrast, we observe rather high levels of variation in the inferred proportion of YAP1 bound to an immobile partner (Supp. Fig. 5G). We additionally show that Venus and mCherry are not subject to such clear differences in their dynamics (Figure 5G & 6C). Together, these analyses support the findings presented based on the light-sheet data and make it more integrated. We are keen to keep the light-sheet data in the manuscript as they

© 2021. Published by The Company of Biologists under the terms of the Creative Commons Attribution License (https://creativecommons.org/licenses/by/4.0/).

demonstrate the applicability of the method in 3D, whereas FRAP and FLIP methodology are not well suited to 3D imaging.

COVID note – The pandemic precluded trying to travel to Janelia Farm to run further light-sheet experiments.

5. The “Differential equation to model nuclear import and export” section should be re-written, or the title of this section should be changed to reflect the fact that there is substantially more in this section rather than just the equations. This reviewer suggests the comparison to FLIP be given its own section, as this is key to the validation of the approach.

The reviewer makes a good point and we have re-titled the section (lines 176-177).

6. The authors should discuss how the estimates of YAP1 import/export and TAZ import/export compare with previous measurements in the “Application of opto-release methodology to YAP and TAZ” section. The consistency with FLIP demonstrates internal consistency of the study, but consistency with previous measurements should also be established.

The reviewer raises a good point. The nuclear import and export rate constants that we measure in this study of epithelial HaCaT cells are mostly in the range 0.0025-0.015s^{-1}. This is slightly slower than those that we measured in fibroblastic cells, which are in the range 0.01-0.075s^{-1} (Ege, Dowbaj et al., Cell Systems 2018). This is now mentioned in lines 415-417.

7. As transient transfections were used to create the system, large variations in expression levels between cells in the population are likely. The authors should show data demonstrating that the results are not dependent on the absolute expression levels of the transfected components.

This is a good point. We have now plotted the relationship between metrics and expression level – shown in Supplementary Figure 2B. Moreover, we have undertaken new analysis to determine the level of YAP1 over-expression. We stained cells transiently transfected with Zdk-FP-YAP1 with an antibody that recognises both endogenous and exogenous YAP1. The exogenous Zdk-FP-YAP was also stained for the Flag epitope tag, thereby enabling quantification of the YAP1 levels in transfected vs untransfected cells. These measurements reveal that the exogenous construct is over-expressed between 2-5 fold (Supp. Fig. 3B). When combined with the measurements of the proportion of Zdk- FP released from the mitochondria (~10%) this leads us to the conclusion that we are releasing between 20-50% of the endogenous YAP1 levels. This modest level of release compared to the endogenous further suggests that our measurements are unlikely to be artefactual due to high expression levels.

8. The maximal nuclear accumulation of YAP using this system (for example in Fig 2), is quite low throughout the experiments in the manuscript. It can’t be determined if this was due to low levels of YAP nuclear localization or incomplete release of YAP from the mitochondria due to a defect in the optogenetic tool. The authors should perform experiments distinguishing between these two possibilities.

The reviewer asks a good question. We are confident that the low levels of nuclear accumulation observed are due to the real behaviour of YAP1, not a defect in release from the mitochondria. Indeed, we designed the system so that only a relatively small amount of protein would be released (also discussed in relation to reviewer #1 point#1). We show below for the benefit of the reviewer that YAP1 is largely cytoplasmic in confluent island of HaCaT cells. Moreover, the optogenetic measurements of YAP1 import and export are in agreement with those measured using FLIP (shown in Supp. Fig. 3H), which does not rely on the release of the FP-YAP1 protein from sequestration.

© 2021. Published by The Company of Biologists under the terms of the Creative Commons Attribution License (https://creativecommons.org/licenses/by/4.0/).

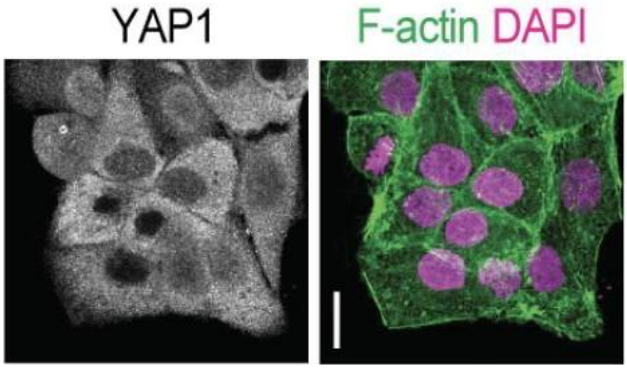

Reviewer Figure 3: Images show endogenous YAP1 localisation (grayscale) and F-actin and DAPI staining (green and magenta, respectively) in HaCaT cells. Scale bar is 25µm.

9. The text in the "Import and Export Rates" section is vague and unorganized. It should be rewritten to provide more precise explanation and interpretation of the data. Also, the title needs adjusting. I believe the main point is the import and export rates are correlated for YAP but not other constructs.

We apologise for the lack of clear structure in this section and have now re-written and re-titled it.

10. The development of the semi-automated software is presented as a significant part of the work. Including a supplemental figure that demonstrates the proper functionality of the software on simulated data would provide definitive proof that the code is functioning properly.

We thank the reviewer for this good suggestion. Supplementary Figure 2 explains how the app functions and in the Figure below we show how the app handles simulated data. The top left panel (labelled 'Simulated data') shows modelling of the protein levels in the different cellular compartments based on the system of ODEs that we use in the manuscript. The panels to the right show exemplars of the simulated data with varying levels of noise artificially added, with the 'experimental noise' being based on the residuals of all fits in our YAP1_WT data. Panel B in the Figure below shows the distribution of import and export rate constants inferred by our app with increasing levels of noise added to the simulated data. When no noise is added (standard deviation = 0) then the import and export rate constants are correctly inferred giving a value of 1. As noise is added the inferred rate constants start to differ slightly from those used in the simulation. However, even with added noise of 0.08, the vast majority of values returned by the app are between 0.7 and 1.4 of the value used in the simulation. The dashed line at 0.023 indicates the level of noise typically found in our data. Thus, we are confident that the model is capable of accurately determining import and export rate constants. If the reviewer and editor think it is appropriate, we can add this analysis to Supplementary Figure 2. In addition to this approach, in the revised manuscript we provide independent FRAP measurements on the mitochondrial off and on rate constants (Figure 1H&I) and confirm that the opto-release inferred nuclear import and export measurements are consistent with FLIP data (Supp. Fig. 3H). Thus, there is orthogonal data to support the validity of the measurements derived using the app.

© 2021. Published by The Company of Biologists under the terms of the Creative Commons Attribution License (https://creativecommons.org/licenses/by/4.0/).

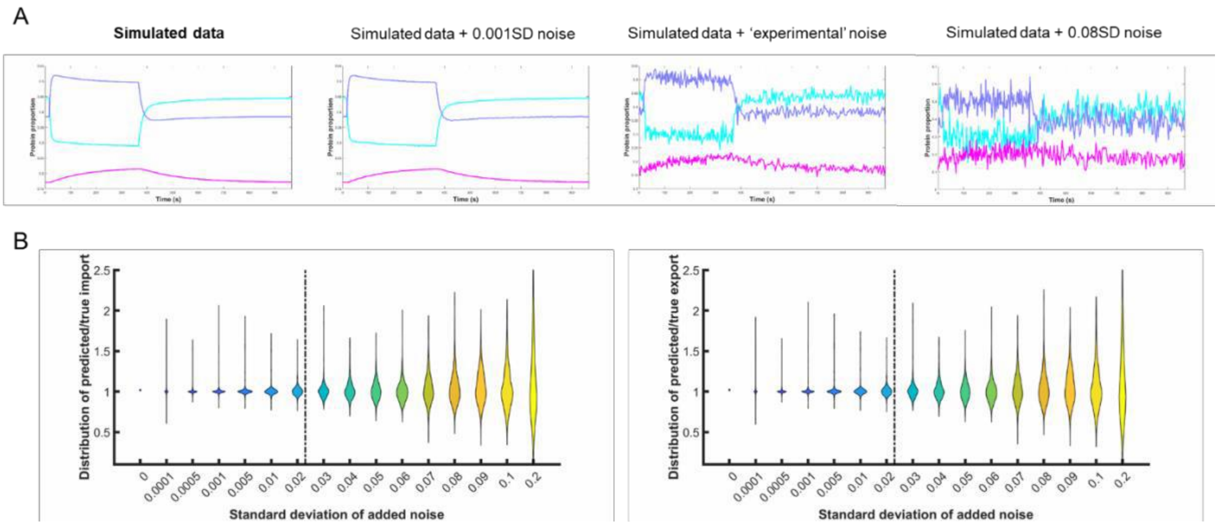

Reviewer Figure 4: Analysis of simulated data. A) Left panel shows the simulated levels of protein in the nucleus (magenta), cytoplasm (lilac), and mitochondria (cyan) based on the ODE system described in Figure 2. The other panels in (A) show the simulated data with increasing levels of noise added. B) 500 simulations for each noise level where analysed using the model-fitting part of the app and the inferred import and export rate constants were divided by the value used in the simulation. The plots show the distribution of inferred import (left panel) and export (right panel) values in simulations with increasing noise. The vertical line indicates the level of noise consistent with the residuals from the fitting of our YAP1_WT data, which provides a good indication of the noise levels in our data.

11. Figure captions generally lack key details, like the number of cells in each experiment and number of experimental days. More detail should be added to these captions.

We have added much greater detail to the figure legends.

12. The availability of the MATLAB code is not stated.

The code is available on github via the following link:
https://github.com/RobertPJenkins/opto_analyser
We have not publicised its availability yet, but will do so in the publication.

MINOR COMMENTS

1. The time courses in all figures should be converted to time from frame number.

We agree with this suggestion. The frame rates are not always the same for every experiment as they depend on the microscope used and sometimes on the size of the opto-release area. Therefore, stating the time is more informative and this is what we now do throughout the manuscript.

2. Is the mCherry data repeated from Figure #1 to Figure #2? If so, this should be stated. Also, how does this effect the statistical comparisons?

The reviewer is correct, we now make that clear in the figure legend for the new Figure 3D (line 565). The statistical comparisons are not affected.

3. In Figure #3C is the distribution of YAP_S94A bimodal?
In the revised manuscript, we have now re-fitted all the data and the YAP_S94A data no longer suggests a bi-model distribution.

© 2021. Published by The Company of Biologists under the terms of the Creative Commons Attribution License (https://creativecommons.org/licenses/by/4.0/).

4. In Figure #5, the positions of the various ROI should be shown. Are the 5 regions equidistant from the nucleus?

We have now generated a figure with the location of the ROIs indicated (Supp. Fig. 5B). The reviewer's question regarding relative distance from the nucleus is a good one. We do not observe any clear relationship between the difference YAP1 behaviours and distance from the nucleus. If there was a simple relationship with distance from the nucleus, then this would be visible in the new 'maps' of the heterogeneity in YAP1 increase in Figure 5G.

5. Figure #1 shows YAP1/TAZ in the schematic but all of the data regard mCherry.

We have now changed Figure 1A to be more generic and it refers to a 'protein of interest'. In response to this and other comments (such as Reviewer #2 comment #7), we have now generated new high- resolution exemplar images for all the figures. Figure 1 now shows an exemplar using Zdk-Venus. The choice of Venus, rather than mCherry, was informed by the desire to simultaneously use Mitotracker Red and DRAQ5.

6. What is shown in Sup Fig #8 and its relevance to the manuscript is unclear.
We have now extensively re-organised the presentation of the light-sheet data and added new analysis of confocal data regarding the different YAP1 behaviours in the cytoplasm. These changes should have addressed the issue with the previous Supplementary Figure 8.

7. Image size/quality is generally-low throughout the manuscript and ideally larger-size images (in both manuscript footprint and quality) should be included.

We have now replaced the images in Figures 1, 2, and 5 with images of higher quality and also given them more space on the page.

8. Page 11, line 276: It might be noted here that YAP does not have a canonical NLS "Importin alpha1 mediates Yorkie nuclear import via an N-terminal Non-canonical nuclear localization signal. J Biol Chem 291, 7926–7937"

This is a good point that we now discuss, with appropriate citation of the paper (lines 424-425).

Reviewer 3 Advance Summary and Potential Significance to Field:
The paper "An optogenetic method for interrogating-…" authored by Dowbaj and colleagues reports on the development and use of an AsLOV-based optogenetic tool to control the cellular localization (including mitochondria, cytoplasm and nucleus) of the YAP transcription factor. Using this approach, the authors quantify, using a MATLAB-based app, the rates of nuclear entry/exit under a variety of conditions. Finally, they combine the optogenetic tool with the use of lighsheet microscopy to measure the dynamics of the transcription factor within the cell in 3D. I think the paper has very interesting and novel elements (such as the use of a YAP optogenetic tool and the capability of its 3D tracking), however the quantification part suffers from multiple fundamental mathematical flaws that unfortunately massively impact the quality of the work. I honestly hope that the points below help the authors with re-analysis.

We were gratified that the reviewer found the manuscript to contain 'very interesting and novel elements'. We also note the significant concerns around some of the mathematical aspects and genuinely thank the reviewer for several suggestions that proved very useful in improving the manuscript.

Reviewer 3 Comments for the Author:
Major points
1. The model has 2 different rate constants for protein unbinding from the mitochondria, depending on whether blue light is on or off i.e. whether the LOV domain is excited or relaxed. However, the rate constant for protein binding to the mitochondria must also take on 2 different values depending on the LOV domain conformation. Without taking this into account, the model is unphysical and, due to all obtained rate constants being interdependent (since they are fit simultaneously), all rate constants obtained with the model in its current form suffer from this.

© 2021. Published by The Company of Biologists under the terms of the Creative Commons Attribution License (https://creativecommons.org/licenses/by/4.0/).

The reviewer makes a good point about allowing for the mitochondrial binding rate constant to vary depending upon blue light illumination. We had initially been reluctant to do this as it increases the degrees of freedom; however, the reviewer's point is correct and we now allow for the mitochondrial binding rate to be different in the lit condition (Figure 2A). The model fitting is now initiated with rates for mitochondrial binding and unbinding starting at the values measured using FRAP methodology (Figure 1H&I). This yields rate constants that are in good agreement with the FRAP measurements (Figure 2C&D). For the benefit of the reviewer, we show in the Reviewer Figure 1 how the old data relates to that in the revised version. The new method also reveals that the mitochondrial binding rate is faster during blue light illumination. However, this is more than compensated for by the faster unbinding rate. This is demonstrated by the ratio of LOV-Zdk binding to unbinding rate constants dropping for all cells when illuminated (right hand panel in the figure below and represented in a different way in Figure 2E).

We additionally explored the benefit of permitting two on rates using Akaike Information Criterion model probabilities. This revealed that when using two free on rates 77.6% of all fits had an AIC probability >0.9, which compared very favourably with using a single free on rate which yielded only 12.9% of fits having an AIC probability >0.9. We thank the reviewer for his/her thoughtful suggestion and we believe that its incorporation has strengthened the manuscript.

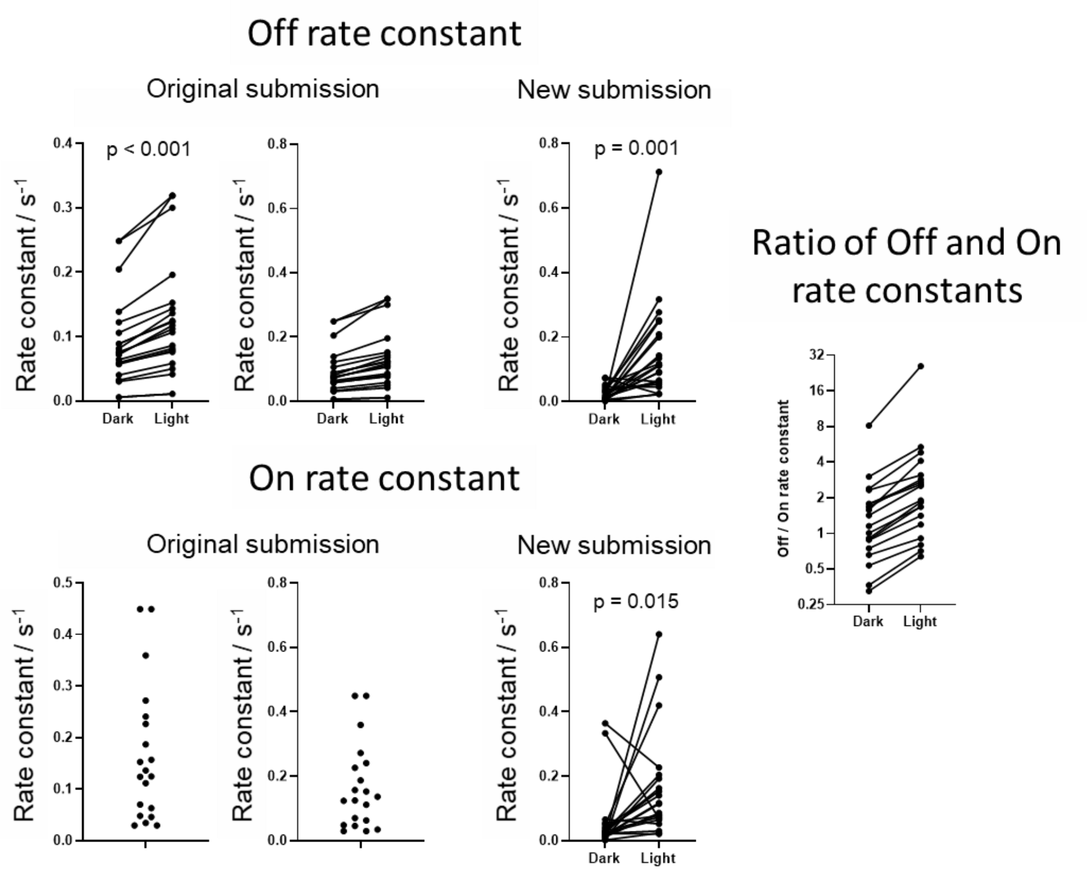

Reviewer Figure 1(reproduced from page 2 for ease of reading): Comparison of original mitochondrial binding and unbinding rate constants with new analysis. Upper panels show Off rate constants and lower panels show On rate constants. Left plot shows the data from the original submission, with lines linking the Dark and Light values for each cell. Middle plots show the same data, but with a scale equivalent to that in the new submission. Off and On rate constant plots are reproduced from the new submission (Figure 2). Right hand plot shows the change in ratio between Off and On rate constants for cells in the Dark and Light. Wilcoxon paired non-parametric statistical testing is reported.

© 2021. Published by The Company of Biologists under the terms of the Creative Commons Attribution License (https://creativecommons.org/licenses/by/4.0/).

2. On lines 756-758, the authors state that relaxation of AsLOV2 domains occurs on the timescale of seconds. Therefore, for illumination to be considered constant, light should be supplied at a rate at least an order of magnitude higher than the relaxation rate, ideally continuously (simply achieved using widefield illumination). If light is supplied to LOV domains at a rate equal to their relaxation rate, between illumination pulses the fraction of stimulated domains will drop exponentially to 0.37=exp(- 1)). This is particularly important given the interconnected nature of the measured rate constants.

The reviewer is correct that there are indeed many pertinent things occurring that impinge upon the release and re-binding of the Zdk-fusion to the LOV domain anchored on mitochondria. This includes the rate at which light induces the conformational change of LOV from its high Zdk affinity state to its lower Zdk affinity state and the reverse relaxation in the dark, which the reviewer raises in his/her comment. In addition, there is the complexity of the illumination regime, which is discontinuous at two levels - the blue light is off during the image acquisition and during the opto-release illumination it is raster scanned across the region of interest - the rather severe challenges of fitting such a complex oscillating regime are well articulated by Pitt and Banga (BMC Bioinformatics 2019). On top of this, there are potential complications caused by rapidly changing mitochondrial shape. Thus, the question about the validity of reducing this to a single binding/unbinding rate constant is a good one. The importance of this matter prompted us to implement an entirely distinct method to measure on and off rate constants in the dark and the light. Crucially, this method used uniform LED illumination to trigger release, which is in line with the reviewer's suggestion. It also accounted for diffusion. Reassuringly, the results of this analysis are concordant with the measurements using our opto-release modelling (Figure 1H&I and Figure 2C&D). With FRAP methodology reporting the off-rate constants to be 0.01-0.02s^{-1} in the dark and 0.03-0.075s^{-1} in the light and the opto-release methodology reporting 0.005-0.07s^{-1} in the dark and 0.024-0.9s^{-1} in the light. The main difference is the wider range of values reported using the opto-release methodology. Overall, these analyses provide orthogonal validation of our approach.

The reviewer suggests widefield illumination, which has a logic to it. However, when we considered the practicalities of this we ran into several issues. These included the lack of optical sectioning, which is a problem when working with epithelial cells with significant amounts of cytoplasm above and below the nucleus, and the challenges of capturing all the different channels simultaneously using filters and dichroics. Sequential capture would be a possibility, but this would lead to discontinuous blue light illumination, which is exactly the problem that the reviewer poses. For our new FRAP analysis, we removed the condenser from an inverted confocal manually positioned a blue LED array on top of the stage. The LEDs were then manually controlled. While this approach was successful for the FRAP analysis, without major engineering it is not possible to integrate the control of an external LED array with the manufacturer's software for confocal microscopes. In the absence of such integration, toggling the blue light on and off during confocal acquisition in a precise manner is not possible. Thus, to conclude this long response, we provide evidence supporting the validity of our approach and would like to highlight that our methodology is designed to be implemented on regular confocal microscopes without bespoke modification.

3. A particularly worrying point is that, after analysis (in Fig. 1F for mCherry and other figures for other constructs) there is no significant difference in the rate constant of mitochondrial release when the light is on ("mito light") or off ("mito dark"). The former should be many times greater than the latter as they are, these values would state that stimulation does not work.

The reviewer raises an important point (similar to that made by reviewer#1 point#1) that we address in several ways.

1. We have implemented a new orthogonal new FRAP data to analyse the difference in binding and unbinding of LOV to Zdk in the dark and light (described in the response to the point above).
2. We also realised that the presentation of data in the original Figure 1F was sub-optimal. It should have been plotted as paired measurements, which is entirely appropriate as the on and off rate are measured in the same cell in the dark and light. As shown in response to reviewer #3 point #1, when plotted and analysed in this manner there is a highly

© 2021. Published by The Company of Biologists under the terms of the Creative Commons Attribution License (https://creativecommons.org/licenses/by/4.0/).

significant increase in off rate is seen in the dark.

3. Thanks to the reviewer's great suggestion, in our new methodology for fitting both the on and off rate constants can vary between the light and the dark states. These are now plotted in Supp. Fig. 3G, 4B, & 6. In all cases, there is a highly significant increase in the off rate constant under blue light. The on rate constant also increases, but this is less dramatic and not always statistically significant. Figure 2E shows that the overall effect of the changes in both off and on rate constants under blue light always favours the release of the Zdk peptide. The reviewer may perhaps expect a bigger difference in the off-rate constant between dark and light; however, we selected the Zdk2 / LOV domain pairing for stability not dynamic range. The incomplete release of the Zdk2 / LOV domain pairing is shown in the original Wang et al., Nature Methods 2016 publication. The images in this paper suggest that it takes a couple of seconds for the dissociation to occur, which is only marginally faster than the timing predicted based on the rate constants that we measure. This work also reports that optimal dissociation is achieved when the Zdk is anchored to the mitochondria, but it in this work we swap the Zdk peptide onto the protein of interest. This is to avoid adding the larger LOV domain to the protein of interest.

4. The rate constants (k's) found here have inherent dependence on cellular parameters such as cytoplasmic/nuclear volume. This would be clear if the differential equations were derived from first principles. E.g. Timney et al. JCB 215, 57 (2016) use differential equations derived from first principles, so measured rate constants can be transformed into quantities independent of nuclear/cytoplasmic volumes and number of NPCs etc. Only after a transformation such as this can correlations be investigated. A similar transformation needs to be applied to rate constants measured in this study before any correlations between rate constants and cellular parameters, or between import and export rate constants, can be performed fairly.

The reviewer is correct that there are a multitude of factors that will influence the rate constants that we derive experimentally. As he/she states, these have been investigated in elegant detail by Timney et al. Our goal is this work is to develop a set of molecular tools that can be used to interrogate the transit of two proteins simultaneously between cellular compartments, to provide a simple analytical tool to derive rate constants relating to nuclear entry and exit, and to demonstrate the utility of the method using YAP1, YAP1 mutants, and TAZ. Our goal is not to study the details of transit through the nuclear pore complex in great detail. While we agree that there would be value and interest in applying the methods of Timney et al., using our tools, this is not our goal in this report and would require the acquisition of considerable additional information about nuclear surface area, the volume of the cytoplasm that is inaccessible to YAP1 due to the presence of other organelles, the number and occupancy of YAP1 binding sites on chromatin in the nucleus, and so on. This sort of analysis would constitute an entire manuscript in its own right.

We agree with the reviewer that it is important to discriminate which of the correlations that we report in Figure 4 are trivial, such as between area and perimeter, and which are unexpected and potentially interesting. In the revised manuscript we have made several changes in our presentation of the correlations.

1. We have moved them to the supplementary figures (Supp. Fig. 4) to reflect that the analysis of the correlations is not the primary objective of this manuscript.
2. Re-plotted them such that trivial correlations between metrics (such as area and perimeter) are no longer shown.
3. Concentrated our focus on the correlation of import and export, which is repeatedly observed and confirmed in multiple experiments. We believe that this is of interest because of the wide range of import and export values. This does not follow from first principles and likely reflects that differences in either the functionality or integrity of the nuclear envelope and its pore complexes between cells. Sources of such variation could include transient rupture of the nuclear envelope, which has been reported by the Piel, Lammerding, and Petronzcki groups (to highlight a few), or differential levels of mechanical stress of the nucleus, which was reported by the Roca-Cusachs group.
4. The other correlations that are evident involve the import/export ratio and the Nuc/Cyto area and Nuc/Cyto concentration. The construction of the ODE system dictates that the import/export ratio will equal the Nuc/Cyto total protein ratio. The latter is the product

© 2021. Published by The Company of Biologists under the terms of the Creative Commons Attribution License (https://creativecommons.org/licenses/by/4.0/).

of the concentration and area. Thus, at one level the correlation is entirely trivial and simply a reflection that our experimental data is fitting our model well. However, the model and system of ODEs does not indicate whether change in the Nuc/Cyto total protein are driven by changes in concentration or changes in the relative areas of the compartments. Our analysis reveals that the Nuc/Cyto area ratio correlates with the import/export ratio, but does not correlate with the Nuc/Cyto concentration. These experimental measurements, which are independent of the model fitting, indicate that cells are able to maintain relatively stable concentrations of proteins in the nucleus and cytoplasm even if the relative size of the compartments varies. This is not something that is predictable from our system of ODEs. Therefore, we believe that this is of biological interest. Clearly, a further investigation of this homeostatic mechanism would be of interest, but is beyond the scope of this work.

Other major points
Major.
5. The methods of “bleaching intensity normalisation” and “non-conserved intensity correction” are overcomplicated and introduce a troubling number of free parameters into the data processing. If simply c(t)/m(t)/n(t) are the cytoplasmic/mitochondrial/nuclear intensities, normalised by their combined intensity (i.e. whole cell intensity) then photobleaching will automatically be accounted for and c(t)+m(t)+n(t)=1 at all times, thus an outflow-inflow function is not required.

The reviewer raises the issue of correcting for bleaching and other variations in the total signal during analysis. We have now completely overhauled the normalization and re-analysed all the original data. Of particular note, we have done the following:

1. Removed the outflow-inflow function as the reviewer suggested.
2. Clarified the normalization process. In brief, we normalize to the combined intensity of the cytoplasm, nucleus, and mitochondria. To account for photobleaching and to smooth out noise in the total intensity value, we fit two functions; one that describes the bleaching during the blue light illumination and the other describes bleaching in the absence of blue light illumination. These functions are used to generate a ‘smoothed’ value of the total intensity at each time point and this is used for the normalization.
3. We have analysed how robust the metrics that we are inferring are to different normalization methods. For the benefit of the reviewer, we present below the export and mito off values for Zdk-mCherry and Zdk-mCherry-YAP1 using a range of different normalization methods. This shows that the signal extraction was robust to different segmentation and normalisation methods with minor differences in distribution of each of the above metrics. Visual inspection suggested that using a moving percentile window size of half the total movie length alongside bi-linear normalisation may lead to marginally superior results. The bi-linear normalization reflects the possibility that there might be a subtle increase in photobleaching as a result of blue light illumination. The inflow-outflow function or resizing the mitochondria (to possibly take account of Zdk binding to only the surface of mitochondria, not the whole area that is measured) made very little difference to the model fit. As the reviewer suggests, we did not implement them because of the cost of adding extra degrees of freedom to the model fitting. Nonetheless, the Figure below indicates that the use of slightly different methods in the original submission would not have yielded wildly different results.

© 2021. Published by The Company of Biologists under the terms of the Creative Commons Attribution License (https://creativecommons.org/licenses/by/4.0/).

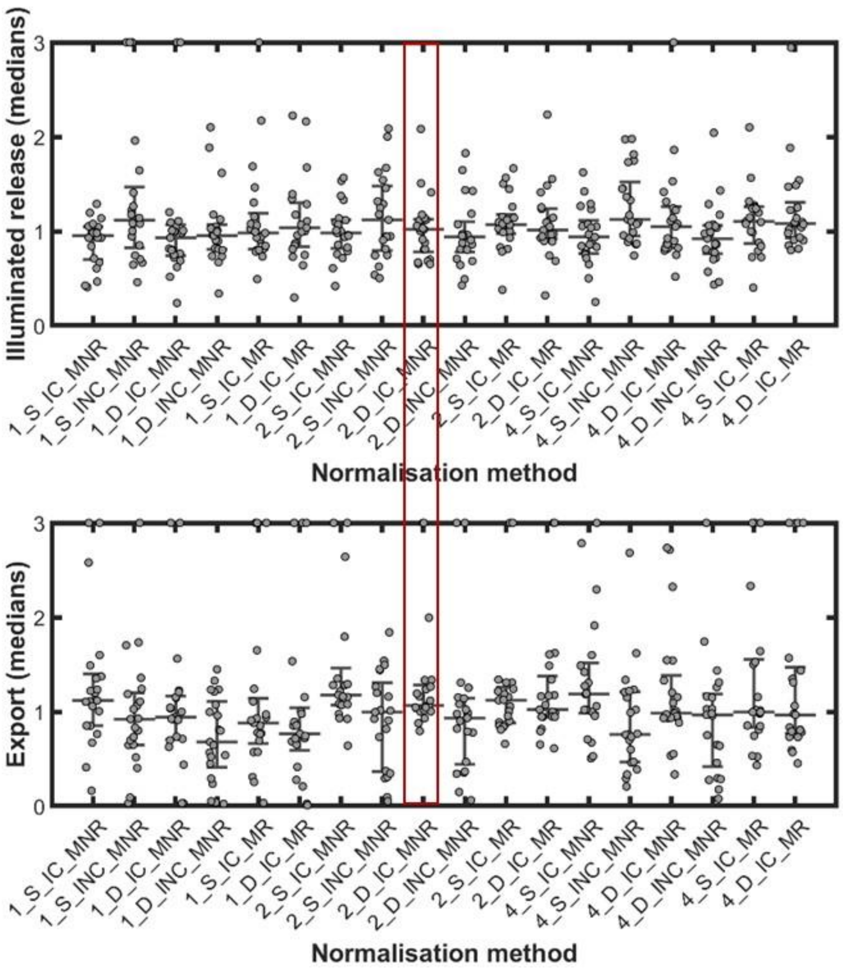

Reviewer Figure 4. Plots show the normalised (to the median of all points) off rate constants when illuminated and export rate constants for Zdk-mCherry-YAP1 when subject to different normalisation methods. The labelling is as follows:

- *1,2, or 4 refers to the number of spatial anchor points for segmentation*
- *S or D refers to whether photobleaching was fitted at a single rate for the whole experiment (S) or with different rates for the blue light and dark phases (D)*
- *IC or INC refers intensity conservation (IC) or non-conservation (INC – inflow/outflow function)*
- *MR or MNR refers to mitochondrial rescaling (MR) or signal kept at extracted levels (MNR)*

The red rectangle indicates the method that we now employ throughout the manuscript.

6. Fig. 1B supposedly shows H2B-mTurquoise labelling the nucleus. Can the authors also show this channel individually (not merged), as it has a sparse, speckled appearance? In addition, with reference to lines 760-762, it is hard to see how imaging of H2B-mTurquoise does not interfere with optogenetic activation despite using the same wavelength. Fluorescent imaging typically needs much higher intensity than optogenetic stimulation.

The reviewer is correct that mTurquoise excitation could interfere with the optogenetic release. To achieve the imaging of the nucleus, mitochondria, and two different Zdk-tagged fluorophores requires four channels and we solved this by using mTurquoise with very low excitation and high gain. This level of illumination did not interfere with the intentional opto-release mechanism. However, the result of very low illumination and high gain for H2B-mTurquoise is low quality images – hence the speckled appearance that the reviewer comments up. In the methods, we explain that nuclear segmentation was based upon integrating the nuclear images over time to generate a smaller number of higher quality images that were used for image segmentation.
In addition to the explanation provided above, we have also replaced the images referred to with

© 2021. Published by The Company of Biologists under the terms of the Creative Commons Attribution License (https://creativecommons.org/licenses/by/4.0/).

higher quality images. In the case of single Zdk-fusion experiments, we now show images that use the near infra-red fluorophore DRAQ5 to label the DNA in the nucleus. Finally, we have expanded the text regarding the choice of fluorophores and articulate that if only a single protein is to be tracked then Venus/Mitotracker Red/DRAQ5 is probably the optimal choice (lines 753-756).

7. There are 2 examples of data being processed inequivalently: lines 160-162, constant thresholding is applied to some cells, dynamic thresholding to others; lines 1016-1020, an inflowoutflow function is applied to some cells, and not others. Making any comparison or assimilation of data that has not been processed in exactly the same manner is difficult.

As articulated in response to point#5, we have now repeated all the analysis using consistent fitting methods. Reassuringly, all the main conclusions of the analysis presented in the initial submission still stand.

8. Almost every instance of "rate" throughout the paper, in the text and figures, should be "rate constant" – the model used finds rate constants. This distinction is very important and conceptually crucial.

The reviewer makes a correct point and we now refer to rate constants.

9. Throughout this work, data has been normalised, but it is never made clear with respect to what. For example, in Fig. 1C, and similar graphs, the vertical axis is called intensity, but it is clearly normalised (I assume to the intensity of the entire cell, and these numbers represent the fraction of intensity that comes from the mitochondria, cytoplasm, and nucleus, but this is not made clear). Since these values are input into quantitative modelling, it is vital that they are clearly explained. In particular, I wonder whether the data has been normalised by the the cellular area (in confocal miscoscopy) or the cellular volume (in lightsheet microscopy).

We have replaced the plots for all the confocal analysis. They now show the normalised total fluorescent intensity for different compartments. For the light-sheet data, the mean fluorescent intensity is shown for the regions of interest normalised for photobleaching at each individual timepoint. This is appropriate because are primarily seeking to compare different regions of the same cell, and not seeking to fit import and export rate constants.

10. Several statements are not supported by the evidence provided: lines 111-113, neither figure shows information on expected localisation; line 120-122, Fig. S1C does not show enrichment to mitochondria; lines 125-127, Fig. 1B does not show an increase in cytoplasmic fluorescence; lines 228- 231, YAP1_5SA has a low import rate as well as a low export rate, so the claim that nuclear persistence is a result of a low export is not justified; lines 247-250, there is no data/figure to support this claim.

We thank the reviewer for the careful reading of the work and have clarified the issue he/she raised. In particular, we have done the following:

- Replaced all the images in Figure 1 and Supp. Fig. 1 with better ones
- Plotted the increase in cytoplasmic fluorescence for the example now shown in Figure 1C
- We now say that the 'most prominent effect' of the 5SA mutations in reduced nuclear export, which is an accurate reflection of the data and does not make a specific assertion about YAP_5SA nuclear import. For clarification, although the median is lower that for wild-type YAP1, the reduction is only on the edge of being statistically significant (p=0.0544). More generally, the reviewer is completely correct that the nuclear import/export rate constant ratios are not sufficient to explain the variation in nuclear/cytoplasmic distribution. We now discuss this explicitly for YAP1_WT and YAP1_S94A, and speculate that this is likely due to a lack of sequestration in long-lived interactions with partners in the nucleus (lines 430-433).
- We have now re-written the section on correlations between metrics. The original claim was based on the figure summarising the Pearson correlation metrics and their statistical significance. In the re-submission, this claim is supported by the lowest right circles in the plots in Supp. Fig. 4.

© 2021. Published by The Company of Biologists under the terms of the Creative Commons Attribution License (https://creativecommons.org/licenses/by/4.0/).

Second decision letter

MS ID#: JOCES/2020/253484

MS TITLE: An optogenetic method for interrogating YAP1 and TAZ nuclear-cytoplasmic shuttling.

AUTHORS: Anna M Dowbaj, Robert P Jenkins, Daniel Williamson, John M Heddleston, Alessandro Ciccarelli, Todd Fallesen, Klaus Hahn, Reuben Odea, John King, Marco Montagner, and Erik Sahai
ARTICLE TYPE: Tools and Resources

We have now reached a decision on the above manuscript.

To see the reviewers' reports and a copy of this decision letter, please go to: https://submit-jcs.biologists.org and click on the 'Manuscripts with Decisions' queue in the Author Area. (Corresponding author only has access to reviews.)

As you will see, one of the reviewers was unavailable, one is completely supportive, and one is 98% supportive and asks that you attend to two small details to improve clarity for readers. I hope that you will be able to carry these out because I would like to be able to accept your paper, depending on further comments from reviewers. If you disagree with either point you can instead rebut and explain why your current approach is preferable.

We are aware that you may be experiencing disruption to the normal running of your lab that makes experimental revisions challenging If it would be helpful, we encourage you to contact us to discuss your revision in greater detail. Please send us a point-by-point response indicating where you are able to address concerns raised (either experimentally or by changes to the text) and where you will not be able to do so within the normal timeframe of a revision. We will then provide further guidance. Please also note that we are happy to extend revision timeframes as necessary.

Please ensure that you clearly highlight all changes made in the revised manuscript. Please avoid using 'Tracked changes' in Word files as these are lost in PDF conversion.

I should be grateful if you would also provide a point-by-point response detailing how you have dealt with the points raised by the reviewers in the 'Response to Reviewers' box. Please attend to all of the reviewers' comments. If you do not agree with any of their criticisms or suggestions please explain clearly why this is so.

Reviewer 1

Advance summary and potential significance to field

This manuscript describes a novel approach to study protein dynamics, and especially nucleo-cytoplasmic shuttling of proteins, with the help of optogenetics.

Comments for the author

The authors have addressed my main concerns in the revised version of their manuscript.

Reviewer 2

Advance summary and potential significance to field

SUMMARY The manuscript is significantly improved, and, in the opinion of this reviewer, almost most ready for publication.

Comments for the author

MAJOR CONCERNS None. The authors should be commended for a complete and thorough response. MINOR CONCERNS Here are some minor suggestions that may help clarify some aspects of data presentation.

© 2021. Published by The Company of Biologists under the terms of the Creative Commons Attribution License (https://creativecommons.org/licenses/by/4.0/).

1. The authors have done an excellent job justifying and explaining why only a small fraction of the YAP signal changes with release. However, the accumulation of YAP in the nucleus upon release is still hard to see in the images. In Fig 1B and 1C adding an inset (perhaps in the bottom right corner of the images) displaying the nuclei at different brightness scale would enable visualization of increased intensity in nucleus. Currently the changes in the images are dominated by the larger variations in the mitochondria.
2. There is large variability in the data. To accommodate this, the authors have broken the axis in Fig 2f. Perhaps use a log-scale axis would be a more quantitative means to display the data.

Second revision

Author response to reviewers' comments

Reviewer 1 Advance Summary and Potential Significance to Field:
This manuscript describes a novel approach to study protein dynamics, and especially nucleo-cytoplasmic shuttling of proteins, with the help of optogenetics.

Reviewer 1 Comments for the Author:
The authors have addressed my main concerns in the revised version of their manuscript.

We are delighted that the reviewer is happy with the revised manuscript.

Reviewer 2 Advance Summary and Potential Significance to Field:
The manuscript is significantly improved, and, in the opinion of this reviewer, almost most ready for publication.

MAJOR CONCERNS
None. The authors should be commended for a complete and thorough response. We are gratified that the reviewer finds our response complete and thorough.

MINOR CONCERNS
Here are some minor suggestions that may help clarify some aspects of data presentation.

4. The authors have done an excellent job justifying and explaining why only a small fraction of the YAP signal changes with release. However, the accumulation of YAP in the nucleus upon release is still hard to see in the images. In Fig 1B and 1C adding an inset (perhaps in the bottom right corner of the images) displaying the nuclei at different brightness scale would enable visualization of increased intensity in nucleus. Currently the changes in the images are dominated by the larger variations in the mitochondria.

We understand the reviewer's point of view and explored changing the brightness and contrast; however, this resulted in images that looked excessively manipulated and seems contrary to the JCS advice on image and blot presentation. Instead, we have added a sentence to the text (lines 127-128) explaining why there is only a small change in the nuclear intensity and highlighted that it can be robustly quantified, as is shown in Figure 1D.

5. There is large variability in the data. To accommodate this, the authors have broken the axis in Fig 2f. Perhaps use a log-scale axis would be a more quantitative means to display the data.

We thank the reviewer for this suggestion and have done has he/she suggested.

© 2021. Published by The Company of Biologists under the terms of the Creative Commons Attribution License (https://creativecommons.org/licenses/by/4.0/).

Third decision letter

MS ID#: JOCES/2020/253484

MS TITLE: An optogenetic method for interrogating YAP1 and TAZ nuclear-cytoplasmic shuttling.

AUTHORS: Anna M Dowbaj, Robert P Jenkins, Daniel Williamson, John M Heddleston, Alessandro Ciccarelli, Todd Fallesen, Klaus Hahn, Reuben Odea, John King, Marco Montagner, and Erik Sahai
ARTICLE TYPE: Tools and Resources

I am happy to tell you that your manuscript has been accepted for publication in Journal of Cell Science, pending standard ethics checks. I reviewed the changes myself and so there are no reviews to return.

© 2021. Published by The Company of Biologists under the terms of the Creative Commons Attribution License (https://creativecommons.org/licenses/by/4.0/).
